# Ultrafast directional hot carrier transfer in bilayer perovskite heterojunctions

**DOI:** 10.1038/s41467-026-76790-z

**Published:** 2026-09-07

**Authors:** Shangpu Liu, Changhao Gao, Mohammad Gholipoor, Tonghan Zhao, Andrii Shcherbakov, Zhiwei Lei, Pirmin Tischler, Yiqin Xie, Nils W. Rosemann, Uli Lemmer, Yang Li, Felix Deschler, Ulrich W. Paetzold

**Affiliations:** 1 Institute of Microstructure Technology, Karlsruhe Institute of Technology, Hermann-von-Helmholtz-Platz 1, 76344 Eggenstein-Leopoldshafen, Germany; 2 Light Technology Institute, Karlsruhe Institute of Technology, Engesserstraße 13, 76131 Karlsruhe, Germany; 3 Physikalisch-Chemisches Institut, Universität Heidelberg, Im Neuenheimer Feld 229, 69120 Heidelberg, Germany; 4 College of Materials Science and Engineering & Engineering Research Center of Alternative Energy Materials & Devices of Ministry of Education, Sichuan University, Chengdu, 610065 China; 5 Physics Department and HK Institute of Quantum Science & Technology, The University of Hong Kong, Pokfulam road, Hong Kong, China

**Keywords:** Materials science, Optics and photonics

## Abstract

Hot carrier dynamics in semiconductors govern unique mechanisms in high-performance devices with potential to exceed thermodynamic limits of optoelectronic device efficiency. Here, we report ultrafast directional transfer of hot carriers within laminated bilayer CsPbBr_3_/FAPbBr_3_ perovskite heterojunctions. Using ultrafast spectroscopy, we show that hot carriers generated in the FAPbBr_3_ layer are directed to the CsPbBr_3_ layer within picoseconds. This process is driven by differences in cooling pathways that create density gradients for directional hot carrier transfer. Such ultrafast hot carrier inter-junction transfer boosts the carrier density in CsPbBr_3_ layer to the level of population inversion. By fabricating perovskite heterojunction vertical-cavity surface-emitting lasers, we achieve hot-carrier laser devices with threshold down to 1.4 μJ cm^−2^ under nanosecond pulsed excitation, five times lower than that of individual perovskite layers. Our findings establish hot carrier transfer in hybrid perovskites as a promising strategy for high-performance hot-carrier lasers, ultrafast laser diodes, and photodetectors.

## Abstract (web summary)

Hot carriers could raise semiconductor optoelectronic efficiency beyond thermodynamic limits, but controlling their rapid cooling and transport is difficult. Liu et al. directed carriers across a laminated perovskite junction within picoseconds and built low-threshold hot-carrier lasers.

## Introduction

The investigation of hot carrier dynamics in semiconductor heterojunctions has become a central focus in developing next-generation high-performance optoelectronic devices^1,2^. Hot carriers—electrons and holes with excess energy above the band edge—can be transferred across interfaces before thermalization, offering opportunities to surpass the conventional limits of device performance^3–6^. For instance, researchers have demonstrated that hot electron transfer driven by Auger recombination or energy level alignment can facilitate carrier funnelling in various material systems, including quantum wells and two-dimensional (2D) materials^3,7–9^, leading to enhanced device efficiencies. However, most applications of hot carriers are reported in microstructures, such as 2D materials and nanostructures^5,10,11^, with few studies on bulk materials, which significantly limits their practical implementation. The main challenges for hot carrier extraction and application are that hot carriers tend to thermalize rapidly within picosecond timescales, which limits their transport lengths and hinders efficient transfer across interfaces before losing their excess energy. Thus, tailoring interfaces that can effectively facilitate hot carrier transfer without significant recombination or energy loss remains important.

Metal halide perovskite materials have been proven suitable for studying hot carrier dynamics because their electronic structure and weak carrier-phonon coupling allow for prolonged carrier lifetimes and efficient energy transfer, making it possible to observe and harness hot carriers before thermalization^12–14^. Significant progress has been made in the fabrication of perovskite planar heterojunctions with controlled interfaces and high structural quality. Various techniques—including solution processing, lamination, vapor-assisted growth, and thermal annealing—have been employed to create layered perovskite structures with minimal defects and well-defined band alignments^15–20^. Such well-engineered heterostructures are crucial for controlling hot carrier migration and interfacial charge transfer, as they enable efficient energy funnelling and charge separation.

In this work, we investigate hot carrier transfer mechanisms in bilayer three-dimensional perovskite heterojunctions composed of CsPbBr_3_ and FAPbBr_3_. We focus on driving ultrafast hot carrier migration from low-bandgap FAPbBr_3_ layers to high-bandgap CsPbBr_3_ within a few picoseconds by using intrinsic differences in hot carrier cooling pathways—specifically, the hot phonon bottleneck effect in hybrid FAPbBr_3_ and the rapid relaxation in inorganic CsPbBr_3_. Using state-of-the-art transient absorption spectroscopy complemented by comprehensive structural characterization, we confirm the formation of high-quality bilayer heterostructures that facilitate this rapid charge transfer. Notably, we demonstrate that hot-carrier transfer significantly enhances optical gain and reduces ASE and lasing thresholds, highlighting a promising strategy to improve the efficiency of perovskite optoelectronic devices. Our findings open new pathways for exploiting hot-carrier dynamics in perovskite heterostructure systems to realize low-threshold lasers and other high-performance hot-carrier applications.

## Results

### Carrier transfer mechanisms in semiconductor heterojunctions

**Fig. 1:**
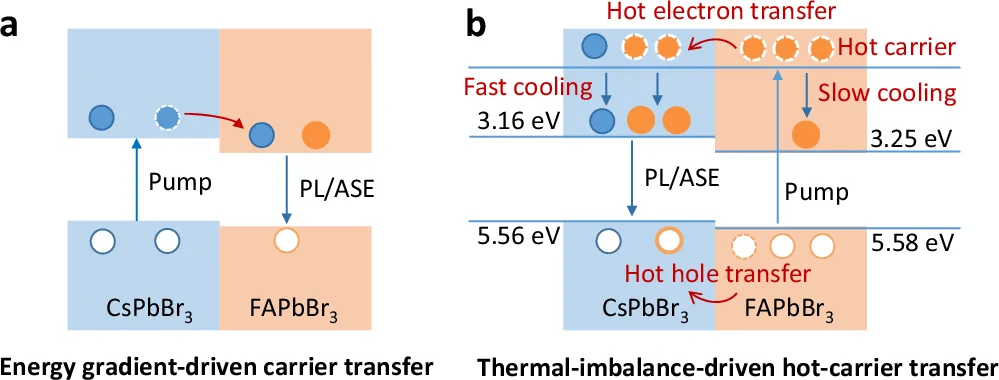
Carrier transfer mechanisms in semiconductor heterojunctions. **a** Schematic illustration of conventional carrier transfer between high and low band gap semiconductors. Red arrows highlight the flow of cooled carriers across the interface, driven by the band energy gradient. **b** Hot carrier transfer from a low band gap (FAPbBr_3_) semiconductor to a high band gap semiconductor (CsPbBr_3_). Under high-energy pumping conditions, hot carriers are generated in both high- and low-band-gap semiconductors, and different hot-carrier cooling rates influence transfer dynamics, directing hot carriers into materials with faster cooling pathways.

Understanding the charge carrier transfer mechanism in semiconductor heterojunctions is crucial for optimizing their electronic and optoelectronic performance, enabling the development of more efficient devices such as solar cells, light-emitting diodes, and lasers^21–23^. Thus, our work starts with a mechanistic discussion of hot carrier transfer in heterojunctions, which serves as the basis for subsequent experimental verification. In most heterojunctions, hot carrier cooling is faster ( < 1 ps) than transfer across the junction, resulting in cooled electrons (holes) migrating from higher CBM (conduction band minimum; or VBM, valence band maximum) to lower CBM (VBM) energy bands (Fig. 1a). The carriers accumulate in the low band gap materials, resulting in stronger PL signals or ASE. However, to fully harness their potential for improving device performance^2,3,10^, it is essential to utilize hot carriers—electrons and holes with excess energy— before thermalization losses.

We hypothesize the hot carrier transfer mechanisms in semiconductor heterojunctions. With excess-energy pumping, both low and high-bandgap semiconductors are excited, resulting in hot carrier generation on both sides (Fig. 1b). Directed hot carrier transfer is then driven by differences in hot carrier cooling rates (thermal imbalance), which are intrinsically related to the materials’ properties. Rapid hot carrier cooling reduces the hot carrier population, whereas slow cooling—such as the hot phonon bottleneck effect^1,11,14^—leads to accumulation of hot carriers within the material. This population imbalance can drive hot carrier transfer from the slow-cooling material to the fast-cooling one, and then hot carriers further cool down at the fast-cooling materials side, thereby enhancing their carrier population at the CB minimum. Notably, the hot carrier transfer can induce substantial population inversion in the excited states, which has been widely reported in III-V semiconductor laser^24–26^. Thus, when combined with bandgap transitions, this hot carrier transfer mechanism effectively creates a three-level system that enhances optical gain and lowers lasing thresholds in heterostructures of slow- and fast-cooling materials. This innovative approach leverages hot carrier dynamics to optimize carrier population distributions and emission processes, offering a promising pathway for the development of hot-carrier-based optical devices and lasing systems with improved efficiencies.

### Formation of bilayer bulk three-dimensional perovskite heterojunctions

**Fig. 2:**
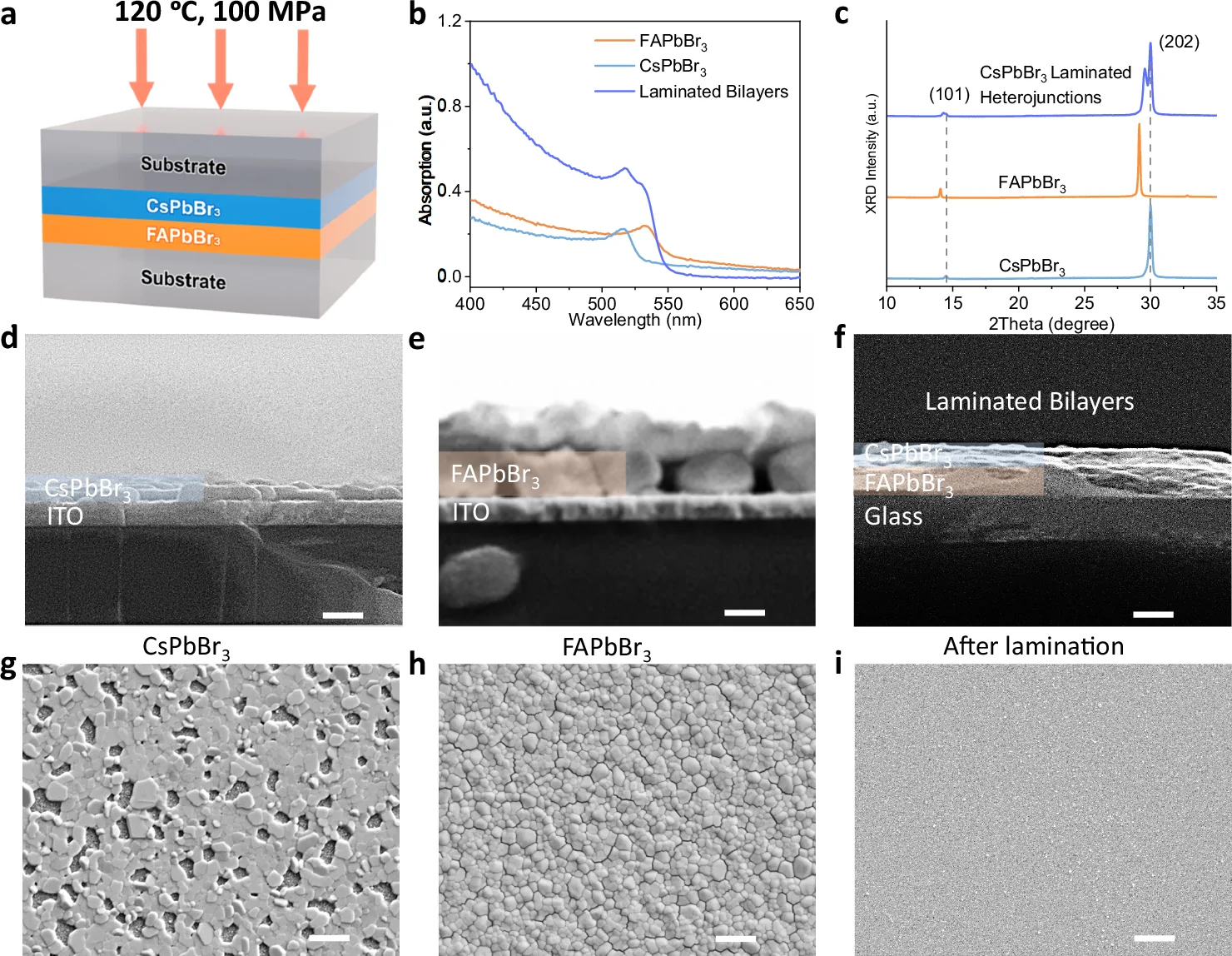
Formation of bilayer bulk three-dimensional perovskite heterojunctions. **a** Schematic illustration of the lamination process used to fabricate bilayer perovskite heterojunctions. **b** Absorption spectra of CsPbBr_3_, FAPbBr_3_, and laminated bilayer films. **c** X-ray diffraction (XRD) patterns of CsPbBr_3_, FAPbBr_3_, and CsPbBr_3_ films after lamination processes. **d–f** Cross-sectional SEM images obtained from CsPbBr_3_ (**d**), FAPbBr_3_ (**e**), and laminated bilayer films (**f**). **g–i** Top-view SEM images of CsPbBr_3_ (**g**), FAPbBr_3_ (**h**), and CsPbBr_3_ films after the lamination process (**i**). Scale bars, 200 nm μm; a.u., arbitrary units.

To experimentally realize directed hot carrier transfer dynamics, we designed and fabricated bulk three-dimensional perovskite heterojunctions, containing CsPbBr_3_ and FAPbBr_3_. Ultraviolet photoelectron spectroscopy (UPS) measurements were employed to determine the band positions of CsPbBr_3_ and FAPbBr_3_ (Supplementary Fig. 1). The results show that the laminated CsPbBr_3_/FAPbBr_3_ heterojunction forms a type II band alignment, characterized by both the VBM and the CBM of CsPbBr_3_ being higher in energy than those of FAPbBr_3_—specifically, by 0.02 eV for VBM and 0.09 eV for CBM (Fig. 1b). This type II band alignment is suitable for the investigation of the hot carrier transfer dynamics. To fabricate high-quality bilayer three-dimensional perovskite heterojunctions, we employed a hot-embossing technique to laminate FAPbBr_3_ onto CsPbBr_3_ thin films (Fig. 2a). Both CsPbBr_3_ and FAPbBr_3_ thin films were initially prepared via spin coating (see details in Method part). To ensure proper heterojunction formation, the lamination process was conducted under elevated temperature and pressure (120 °C, 100 MPa for 5 mins), with the films positioned face-to-face during pressing. Absorption spectra (Fig. 2b) indicate that the band transition positions of the laminated bilayer films closely overlap with those of the FAPbBr_3_ (533 nm) and CsPbBr_3_ films (516 nm). In Fig. 2c, we compare the X-ray diffraction (XRD) patterns of films before and after the lamination process. CsPbBr_3_ films both show a dominant XRD peak at ~30°, which corresponds to the (202) facet. After lamination, we carefully separate the two films physically. A new side peak appears in CsPbBr_3_ films at ~29°, which we attribute to the signal of the residual FAPbBr_3_ on top of CsPbBr_3_ after delamination. These results demonstrate that the lamination process maintains the optical and structural integrity of both constituent films, without the formation of a mixed organic cation phase.

The films exhibit strong differences in morphology and an uninterrupted junction after the lamination process due to recrystallization^27–30^. Figure 2d–f show the cross-sectional structures of CsPbBr_3_, FAPbBr_3_, and laminated bilayer films, which confirm the formation of bilayer perovskite heterojunction structures. Notably, prior to lamination, both CsPbBr_3_ and FAPbBr_3_ exhibit only partial coating of the substrate. The lamination process applies elevated temperature (120 °C) and high pressure (100 MPa) for 5 min. Under these conditions, the perovskite layer undergoes pressure‑induced recrystallization and grain boundary fusion. The combined heat and pressure promote atomic rearrangement, grain rotation, and coalescence, resulting in a much smoother, denser film with grain sizes below 10 nm resolution (Fig. 2g–i). Similar recrystallization phenomena have been reported in laminated perovskite films by several groups^27–30^. Control experiments reveal that hot pressing a CsPbBr_3_ film against a silicon wafer (i.e., without a second perovskite layer) improves its surface roughness, but the smoothing effect is substantially weaker than that observed after lamination with a FAPbBr_3_ layer (Supplementary Fig. 2). Additionally, lamination of thinner films produces a less pronounced morphological change. These observations indicate that the dramatic transformation is driven by recrystallization at the interface when two thick perovskite films are brought into intimate contact under heat and pressure. Importantly, this recrystallization does not degrade their structure and optical properties; as shown in Fig. 2b and c, the absorption spectrum and XRD of perovskite films are unchanged after lamination. Confocal PL mapping indicates that a small amount of FAPbBr_3_ remains on top of CsPbBr_3_ after delamination (Supplementary Fig. 3), which may help in passivating surface traps. Overall, the lamination process preserves the optical and structural properties of both films and further enhances their morphological quality, resulting in flatter and smoother interfaces.

### Hot carrier transfer driven by cooling rate gradients

**Fig. 3:**
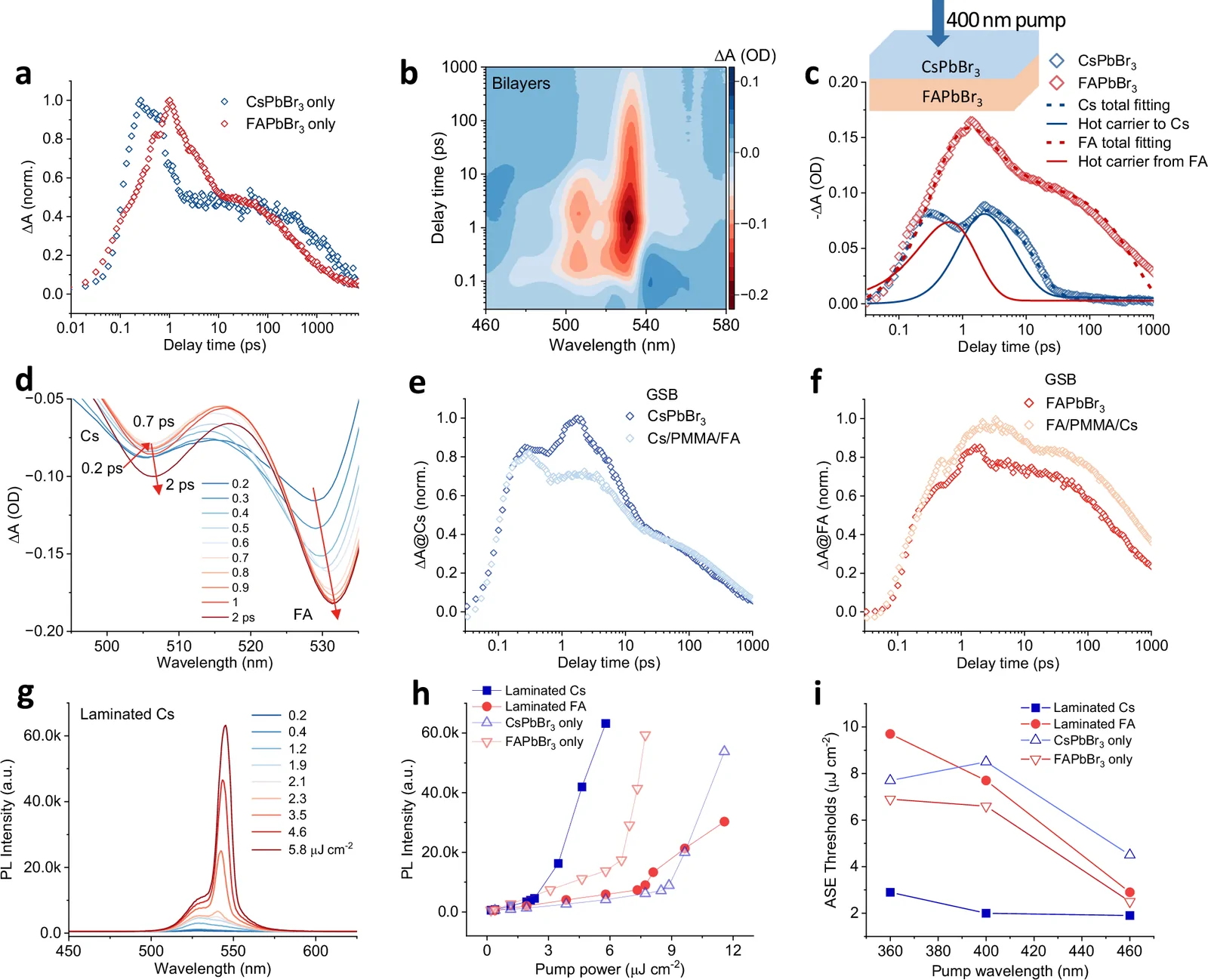
Hot carrier transfer driven by cooling rate gradients. **a** Comparison of TA kinetics between single layers of CsPbBr_3_ and FAPbBr_3_ (400 nm pump, ~25 µJ cm^−2^, signals were collected at their GSB positions). **b–d** TA color maps (**b**), kinetics (**c**), and spectra (**d**) of laminated bilayer thin films. The pump laser (400 nm, ~75 µJ cm^−2^) was directed at CsPbBr_3_ side. Global fitting was used to extract the hot carrier transfer process in total TA signals (see details in the Methods part). The dash line and solid line in (**c**) represent the fitting of total TA signals and the hot carrier transfer process, respectively. **e, f** Comparison of TA kinetics between laminated bilayer thin films and films with the same lamination conditions but with a PMMA block layer, which is designed to act as a blocking layer for the carrier transfer between CsPbBr_3_ and FAPbBr_3_. The TA kinetics were obtained from CsPbBr_3_ (**e**) and FAPbBr_3_ (**f**) side with a pump fluence of ~25 µJ cm^−2^, respectively. **g** Evolution of emission spectra of laminated bilayer thin films with different pump fluences. The pump laser was directed at CsPbBr_3_ side (400 nm, 270 fs pulse, and 5kHz repetition rate). **h** PL intensity as a function of the pump fluence. The data were collected from laminated bilayer films with laser impinging on CsPbBr_3_ sides (Laminated Cs), at FAPbBr_3_ sides (Laminated FA), pure CsPbBr_3_, and FAPbBr_3_ films. a.u., arbitrary units. **i**, Amplified spontaneous emission (ASE) thresholds as a function of the pump wavelength.

We first explore hot carrier dynamics under high-energy excitation in our structures, where hot carriers are generated on both sides of the heterojunction. Using 400 nm excitation in pure CsPbBr_3_ films first, we observe that the carrier decay occurs rapidly within the first picosecond, and then stabilizes until hundreds of picoseconds, consistent with fast hot carrier cooling processes (Fig. 3a and Supplementary Fig. 4). In contrast, in pure FAPbBr_3_ films, the carrier signal exhibits a gradual rise over the initial picosecond followed by a rapid decay within approximately 10 ps. We attribute this behavior to the hot phonon bottleneck effect (phonon reabsorption), which can slow down the carrier cooling process^31,32^. Importantly, normalized TA spectra of FAPbBr_3_ display a continuous red shift up to 50 ps (Supplementary Fig. 5), whereas no significant peak shift is observed in CsPbBr_3_ films. This observation confirms their distinct hot carrier cooling dynamics. We attribute these differences to the presence of organic cations in FAPbBr_3_, which induce strong phonon vibrations within the lattice, while the all-inorganic CsPbBr_3_ exhibits a more rigid lattice and comparatively weaker phonon interactions^33–35^, resulting in carrier relaxation which is less affected by phonon bottlenecks.

We next extend our investigation to laminated heterojunctions to elucidate how interfacial carrier-transfer processes influence hot-carrier dynamics in these architectures. From UPS measurements, we know the energy barrier for electron transfer from FAPbBr_3_ to CsPbBr_3_ is 0.09 eV, while there is almost no barrier for hole transfer from FAPbBr_3_ to CsPbBr_3_ (Fig. 1b). Thus, for hot carriers generated under high-energy excitation (e.g., 400 nm, 3.10 eV), the excess energy ( ~ 0.77 eV above the FAPbBr_3_ CBM) is sufficient to overcome the 0.09 eV electron transfer barrier, enabling efficient directional hot-carrier transfer. Using 400 nm excitation on laminated bilayers with the laser incident from the CsPbBr_3_ side, we observe two ground-state bleach (GSB) signals corresponding to the band transitions of CsPbBr_3_ and FAPbBr_3_ (Fig. 3b). In the FAPbBr_3_ layer, a slow hot carrier cooling process persists, followed by a rapid decay of the hot carrier signal from approximately 1 to 8 ps (Fig. 3c). In contrast, the CsPbBr_3_ GSB exhibits an initial decay within the first picosecond, followed by an increase in signal intensity up to around 2 ps. As proposed in our earlier discussion of the hot carrier transfer mechanism (Fig. 1b), when hot carriers are generated on both sides of the heterojunction, the differing cooling rates drive hot carriers toward the material with the faster cooling rate, such as CsPbBr_3_. We globally fitted the TA kinetics at the FAPbBr_3_ and CsPbBr_3_ GSB positions using a sequential kinetic model to extract the hot‑carrier transfer dynamics (see Methods for details). In Fig. 3c, the red and blue solid lines represent hot carriers transferred out of FAPbBr_3_ and those injected into CsPbBr_3_, respectively. Critically, the blue component constitutes the dominant fraction of the total carriers present in CsPbBr_3_, confirming that hot‑carrier transfer is the primary source of population in the CsPbBr_3_. At early times, hot carriers initially accumulate in the FAPbBr_3_ phase due to the hot‑phonon bottleneck effect, reaching a maximum at ~0.6 ps, whereas hot carriers in CsPbBr_3_ cool very rapidly ( < 0.2 ps). This population imbalance between these hot carrier levels in both materials drives directional hot‑carrier transfer from FAPbBr_3_ to CsPbBr_3_, resulting in a pronounced hot‑carrier population in CsPbBr_3_ that persists until ~2 ps and subsequently decays over approximately 10 ps. The fitted hot‑carrier transfer time constant and carrier decay rates are summarized in Supplementary Table 2. Notably, the hot‑carrier population remains detectable in CsPbBr_3_ for ~9.8 ps, which is considerably longer than the transfer out of FAPbBr_3_ (~1.2 ps). This difference may be attributed to the finite transport time of hot carriers across the heterointerface. Supporting this, the TA spectra of the laminated bilayers display a gradually redshifted and rising FAPbBr_3_ GSB signal (Fig. 3d). Notably, unlike isolated CsPbBr_3_, the laminated CsPbBr_3_ GSB signals initially undergo a continuous red shift (0.2–0.7 ps) accompanied by a decrease in TA intensity, followed by an increase in signal amplitude from 0.7 to 2 ps along with further redshifting. These observations further confirm the occurrence of hot carrier transfer into CsPbBr_3_ within the heterostructure. We also find extended PL lifetimes in laminated CsPbBr_3_ from time-resolved PL spectra, consistent with the carrier transfer into CsPbBr_3_ side (Supplementary Fig. 6). Besides, as depicted in Fig. 1b, due to type II band alignment, in principle, the hole generated in FAPbBr_3_ may also jump to CsPbBr_3_ VB maximum, further increasing their carrier density. However, the conduction band offset (ΔCBM = 0.09 eV) in our CsPbBr_3_/FAPbBr_3_ heterojunction is substantially larger than the valence band offset (ΔVBM = 0.02 eV). Similar perovskite heterostructures have been identified, through both theoretical calculations and experimental measurements, as possessing a quasi‑type‑II alignment^36–38^. A quasi‑type‑II alignment is characterized by partial spatial separation between electron and hole wavefunctions, meaning that significant wavefunction overlap exists. Consequently, electron transfer across the interface is much more favorable than hole transfer. Although CsPbBr_3_ has a high CBM compared to that of FAPbBr_3_, we did not observe the conventional carrier transfer from CsPbBr_3_ to FAPbBr_3_. Global fitting of the hot‑carrier transfer dynamics reveals that both carrier cooling and hot‑carrier injection into CsPbBr_3_ are extremely fast (the transfer time constant is ~9.8 ps, while initial cooling occurs in <0.2 ps in CsPbBr_3_). This enables efficient accumulation of carriers in the CsPbBr3 phase before any significant decay can occur. The TA decay kinetics further show that the carrier lifetime in CsPbBr_3_ is only tens of ps (Supplementary Table 2), which is much shorter than the hundreds of picoseconds observed in FAPbBr_3_. Moreover, most conventional carrier transport across the bilayer heterostructure is reported to occur on timescales longer than 100 ps^39–41^. Any carriers that reach the conduction band edge of CsPbBr_3_ recombine radiatively to contribute to optical gain before they can transfer back to FAPbBr_3_. Thus, the hot‑carrier (both electron and hole) transfer from slower-cooling FAPbBr_3_ to fast-cooling CsPbBr_3_ dominates while other processes (hole transfer from FAPbBr_3_ to CsPbBr_3_ and electron transfer from CsPbBr_3_ to FAPbBr_3_) are negligible under our experimental conditions (Fig. 1b).

To further confirm the hot carrier transfer process within perovskite heterojunctions, we compared the TA kinetics of the laminated sample with a control sample under identical lamination conditions but incorporating an additional spin-coated poly(methyl methacrylate) (PMMA) interlayer between the CsPbBr_3_ and FAPbBr_3_ layers. After inserting the PMMA block layer, the GSB kinetics for both CsPbBr_3_ and FAPbBr_3_ (Fig. 3e, f) layers are close to those of the individual layers shown in Fig. 3a. These results further confirm that hot carrier transfer occurs within the laminated bilayers and that the transfer is hindered when an intervening layer prevents direct interfacial interaction. Importantly, the hot‑carrier transfer process persists for approximately 10 ps, which is fully consistent with our global fitting results (Fig. 3c).

Based on the hot carrier transfer mechanism discussed above, the planar FAPbBr_3_/CsPbBr_3_ heterojunctions are expected to mimic an ideal three-level system. High-energy photoexcitation generates hot carriers in FAPbBr_3_ (level 3), which exhibits a relatively slow cooling rate. These hot carriers are then rapidly funneled into the CsPbBr_3_ side within a few picoseconds, and subsequently cool to the band-edge excited state of CsPbBr_3_ (level 2) on a sub-picosecond timescale. Finally, the carriers radiatively relax to the CsPbBr_3_ ground state (level 1), emitting light (Fig. 1b). Such a three-level configuration would lead to an enhancement of optical gain in CsPbBr_3_.

Figure 3g presents the fluence-dependent PL spectra of laminated bilayer films. As the pump fluence increases (excitation at 400 nm, 260 fs pulse duration, 5 kHz repetition rate), pronounced ASE signals emerge in the laminated samples. We further compare the ASE thresholds obtained from various configurations (Fig. 3h): laminated bilayer films with excitation at the CsPbBr_3_ side (laminated Cs), at the FAPbBr_3_ side (laminated FA), as well as pure CsPbBr_3_ and FAPbBr_3_ films. The results reveal that the ASE threshold for laminated Cs samples ( ~ 2 µJ cm^−2^) is significantly lower than that of isolated CsPbBr_3_ (~9 µJ cm^−2^). Conversely, laminated FA samples exhibit an increased ASE threshold ( ~ 8 µJ cm^−2^), compared to ~7 µJ cm^−2^ for isolated FAPbBr_3_. Additionally, under continuous-wave (CW) excitation, FAPbBr_3_ films display approximately ten times higher photoluminescence quantum yield (PLQY) than CsPbBr_3_ films (Supplementary Fig. 7). These findings further confirm the occurrence of hot carrier transfer from FAPbBr_3_ to CsPbBr_3_ within the laminated structures, which markedly enhances the carrier density at CsPbBr_3_ excited states and facilitates their population inversion and optical gain, thereby contributing to the observed large reduction in ASE threshold, consistent with our simulation results (Supplementary Fig. 8, see details in Supplementary Methods). We further extracted the net optical gain coefficients from our transient absorption data (Supplementary Table 1), which is a core parameter for evaluating the performance of gain media (see details in Supplementary Method part). The results clearly show that the laminated heterojunction achieves a higher net gain coefficient (712 cm^−1^) despite requiring a much lower threshold carrier density (7.3 × 10^17 ^cm^−3^) compared to pure CsPbBr_3_ (255 cm^−1^, 2.6 × 10^18 ^cm^−3^). These results quantitatively confirm that hot‑carrier transfer creates a more efficient multi‑level system, enabling population inversion at lower carrier density with higher optical gain.

To further elucidate the role of hot carrier transfer, we investigate the ASE thresholds of laminated samples by varying the pump wavelength. When comparing higher energy excitations at 360 nm and 400 nm to lower energy excitation at 460 nm, both pure CsPbBr_3_ and FAPbBr_3_ films exhibit a decrease in their ASE thresholds at 460 nm (Fig. 3i). These findings are consistent with previous studies reporting lower thresholds under near-resonant pumping conditions due to accelerated carrier cooling and reduced energy loss to heat^42–44^. In our work, however, we deliberately extract hot carriers from the slow‑cooling material (FAPbBr_3_) and transfer them to the fast‑cooling material (CsPbBr_3_) to further enhance the carrier population in CsPbBr_3_. Consequently, as the pump energy decreases – and thus the population of hot carriers diminishes – the ASE threshold reduction induced by hot‑carrier transfer from pure CsPbBr_3_ to the planar heterojunction becomes less pronounced: the reduction factor is 4.5 at 400 nm but only ~2.5 at 460 nm. Furthermore, for the laminated FAPbBr_3_ samples, we observe a dramatic decrease in the ASE threshold (from ~10 to ~3 µJ cm^−2^) when the pump wavelength is increased from 360 nm to 460 nm. In contrast, the threshold of laminated CsPbBr_3_ samples shows only a weak dependence on pump wavelength (varying from ~3 to ~2 µJ cm^−2^). This behavior is fully consistent with our proposed hot-carrier transfer mechanism. As the pump wavelength increases, two competing effects come into play: an increased carrier cooling rate, which tends to lower the threshold in both CsPbBr_3_ and FAPbBr_3_, and a decreased proportion of transferred hot carriers, which tends to raise (lower) the threshold in CsPbBr_3_ (FAPbBr_3_). The interplay of these effects results in a weak wavelength dependence for the laminated CsPbBr_3_ samples, where the two effects partially cancel. In contrast, the laminated FAPbBr_3_ samples exhibit a much stronger threshold reduction as wavelength increases, driven by the combined effects of an accelerated cooling rate and reduced carrier transfer. This result supports the conclusion that hot-carrier transfer is the primary mechanism responsible for increasing the carrier population and lowering the ASE threshold in perovskite heterojunctions.

Recent studies have suggested that defect states could also form a three‑level system, which might facilitate population inversion^45–48^. In our work, however, the heterojunction itself inherently provides a multi‑level structure. The three‑level laser picture we propose is grounded in the band alignment and hot carrier transfer in the CsPbBr_3_/FAPbBr_3_ heterojunction, not in the presence of defect states. Consequently, defects are not required to explain the observed population inversion or the reduction in lasing threshold. To further rule out possible contributions from defect states, we compared the excited‑state carrier dynamics before and after the lamination process. In both TA and time‑resolved photoluminescence (TRPL) spectra, we observed blue‑shifted signals on the high‑energy tail at early times after lamination (Supplementary Figs. 9 and 10), indicating the contribution of hot carriers transferred into the CsPbBr_3_ side^49,50^. At later times (beyond 1 ns), both TA and TRPL spectra overlapped well on the low‑energy tail, confirming that no additional trap states were introduced and that existing trap states were not strongly passivated by lamination.

Notably, under equivalent low carrier generation densities (Supplementary Fig. 9c), the CsPbBr_3_ GSB signals before and after lamination exhibited similarly rapid decays over the first few picoseconds – a feature typically attributed to trap‑mediated recombination^51,52^ – indicating comparable trap densities. Moreover, at both low and high carrier generation densities, the CsPbBr_3_ GSB decay kinetics remained nearly identical before and after lamination, further demonstrating that the defect density is essentially unchanged by the lamination process. Consistently, after lamination, the CsPbBr_3_ PL decay showed a prolonged lifetime during the initial 4 ns (Supplementary Fig. 10c and f), which we attribute to fast hot‑carrier transfer from the FAPbBr_3_ layer. Beyond 4 ns, the decay kinetics before and after lamination became almost overlapped, confirming that the density of long‑lived trap states remains unaffected^53^. Further, the PLQY of CsPbBr_3_ films remained nearly unchanged after lamination compared to their pre‑lamination values (Supplementary Fig. 7). Taken together, all these results demonstrate that the lamination process does not significantly alter the defect density in CsPbBr_3_. Therefore, the dominant mechanism reducing the ASE threshold in laminated heterojunctions is hot‑carrier transfer driven by cooling‑rate gradients, rather than defect‑mediated processes.

We further investigated the origin of the ASE signal to rule out contributions from reabsorption or mixed emission from the FAPbBr_3_ layer. Under high‑fluence excitation (Supplementary Fig. 11a), the laminated heterojunction exhibits two distinct ASE peaks: one at ~540 nm originating from CsPbBr_3_ and another at ~556 nm from FAPbBr_3_. The FAPbBr_3_ gain peak is well separated from the CsPbBr_3_ gain region, and the absorption edge of FAPbBr_3_ lies at ~530 nm. Hence, the FAPbBr_3_ layer absorbs only weakly at 540 nm – the wavelength at which the heterojunction ASE is observed. To quantify the contribution of each layer, we compared the fluence‑dependent PL intensity at 540 nm for the laminated heterojunction and a pure FAPbBr_3_ film (Supplementary Fig. 11b). The single‑layer FAPbBr_3_ film shows a linear increase in signal intensity up to 100 µJ cm^−2^, with no threshold behaviour. After subtracting the FAPbBr_3_ contribution at 540 nm from the heterojunction signal, the remaining intensity exhibits a clear transition from linear to superlinear growth (slope factor ∼3) at a threshold fluence identical to that of the original data. This confirms that the ASE at 540 nm arises from carriers transferred into CsPbBr_3_, not from direct emission or optical gain from the FAPbBr_3_ layer.

### Hot carrier transfer facilitates lasing in perovskites

**Fig. 4:**
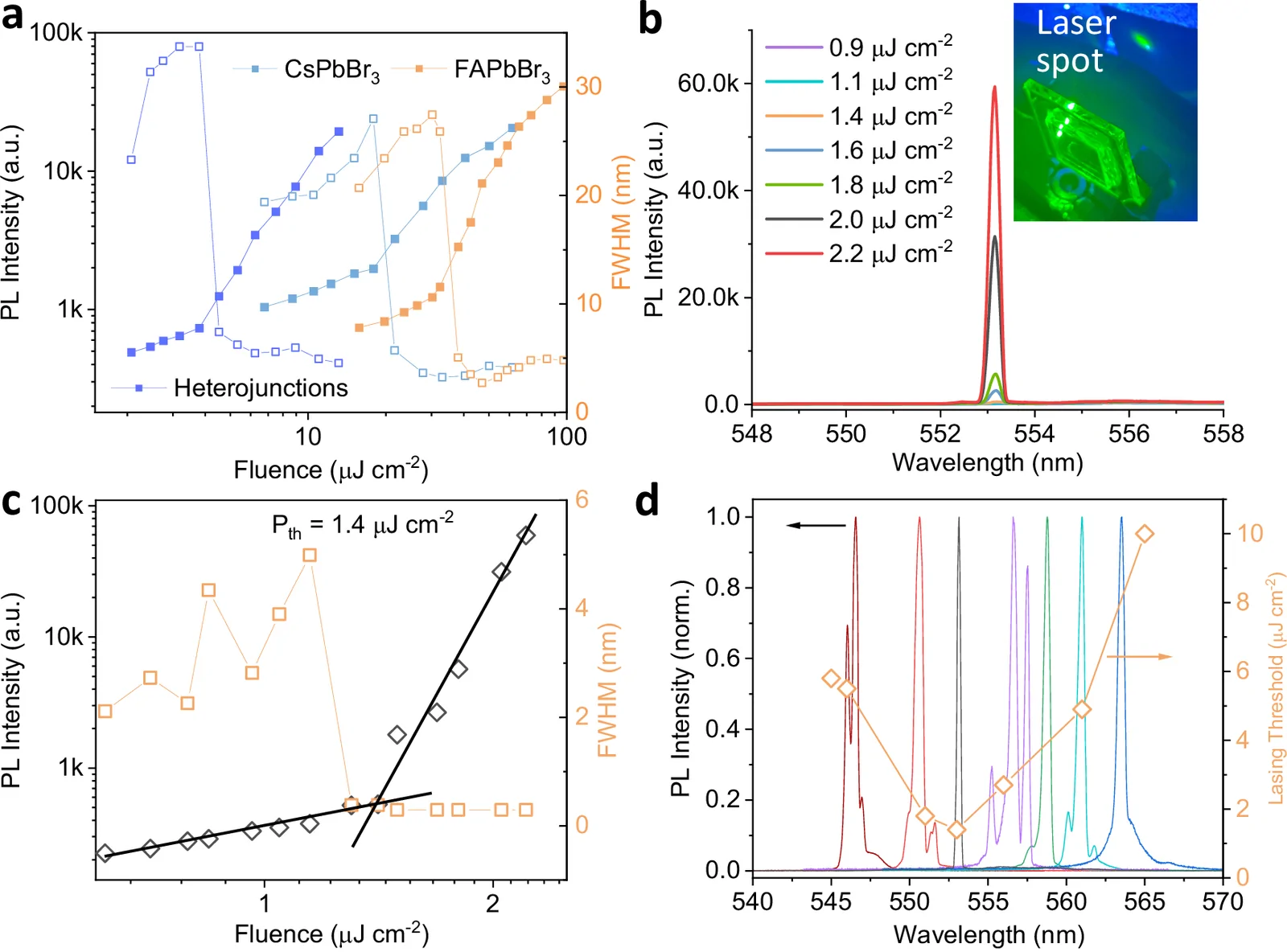
Nanosecond pulsed lasing with decreased thresholds. **a** Comparison of ASE thresholds between laminated bilayer films, pure layers of CsPbBr_3_ and FAPbBr_3_ (355 nm, 1 ns pulsed laser, 1 kHz repetition rate). **b** Evolution of laser emission from laminated bilayer thin films. The inset shows the image of the perovskite VCSEL emission (green spot) under a 355 nm optical pump. **c** PL intensity and FWHM as a function of pump energy density. **d** Lasing spectra and corresponding thresholds obtained from perovskite VCSELs with different MgF_2_ spacer thicknesses. a.u., arbitrary units.

Based on the results above, we exploit the hot carrier transfer mechanism—an approach that significantly reduces the ASE threshold—to advance perovskite lasing structures. Using 355 nm nanosecond pulsed excitation (1 ns pulse width, 1 kHz repetition rate) laminated CsPbBr_3_ samples exhibit a substantially lower ASE threshold ( ~ 4 µJ cm^−2^) compared to individual samples (Fig. 4a). Statistical analysis across multiple devices confirms the reproducibility of this threshold reduction (Supplementary Fig. 12). We further compared the ASE thresholds of laminated CsPbBr_3_/FAPbBr_3_ heterojunction and a control sample with a PMMA interlayer inserted between the two perovskite layers. Both samples underwent the same lamination process; however, the PMMA layer blocks hot‑carrier transfer, thereby disabling the hot-transfer pathway. Consequently, the control sample exhibited no ASE threshold reduction relative to single‑layer CsPbBr_3_ (Supplementary Fig. 12), confirming that the threshold reduction in the laminated heterojunction is primarily attributable to hot‑carrier transfer.

To realize laser emission, we fabricated laminated vertical cavity surface-emitting laser (VCSEL) structures by replacing the substrate with distributed Bragg reflectors (DBR) tuned to the PL emission wavelength region. After the same lamination process, the perovskite heterojunction was sandwiched between two DBR mirrors, forming a high-quality optical cavity with a Q-factor of ~2700.

Figure 4b and c illustrate the evolution of PL intensity and full width at half maximum (FWHM) with increasing pump fluence. Notably, the FWHM narrows from 5 nm to 0.2 nm, and the PL intensity exhibits a superlinear increase—rising from a slope of 1 to 10—once the pump fluence exceeds 1.4 µJ cm^−2^, indicating the onset of lasing. Besides, after this laser threshold, the emitted laser signals become more collimated, with a converged angular distribution (Supplementary Fig. 13). Further tuning of the laser emission was achieved by adjusting the MgF_2_ spacer thickness within the cavity, resulting in color-tunable laser output from 545 to 565 nm (Fig. 4d). The laser nature of the emission is conclusively confirmed by direct coherence measurements. Michelson interferometer reveals a coherence time of ∼2.7 ps above threshold (Supplementary Fig. 14). Together with super-linear power dependence, spectral narrowing to 0.2 nm, thickness dependent lasing output, and a well‑collimated beam with a converged angular distribution, these data unambiguously demonstrate that the laminated heterojunction VCSEL operates as a true laser ^54,55^.

Importantly, the lasing threshold of the laminated heterojunctions ( ~ 1.4 µJ cm^−2^) is considerably lower than that of pure CsPbBr_3_ (~5 µJ cm^−2^) and FAPbBr_3_ VCSELs ( ~ 4 µJ cm^−2^, Supplementary Fig. 15), consistent with the observed trends in ASE thresholds. With a lasing threshold of 1.4µJ cm^−2^ under nanosecond pumping, our laminated heterojunction VCSEL achieves one of the lowest values reported for nanosecond-pumped perovskite VCSELs (Supplementary Table 3). However, continuous-wave operation, essential for practical applications, remains to be demonstrated. This limitation may be addressed by future work on thermal management or low-temperature operation. These findings demonstrate that utilizing hot carrier transfer mechanisms can promote the carrier population inversion at excited states and significantly reduce the lasing threshold in perovskite devices. This approach offers a promising strategy for enhancing a wide range of optoelectronic applications by maximizing the utilization of all generated carriers within semiconductor materials.

### Charge transfer in perovskite heterojunctions with resonant pumping

**Fig. 5:**
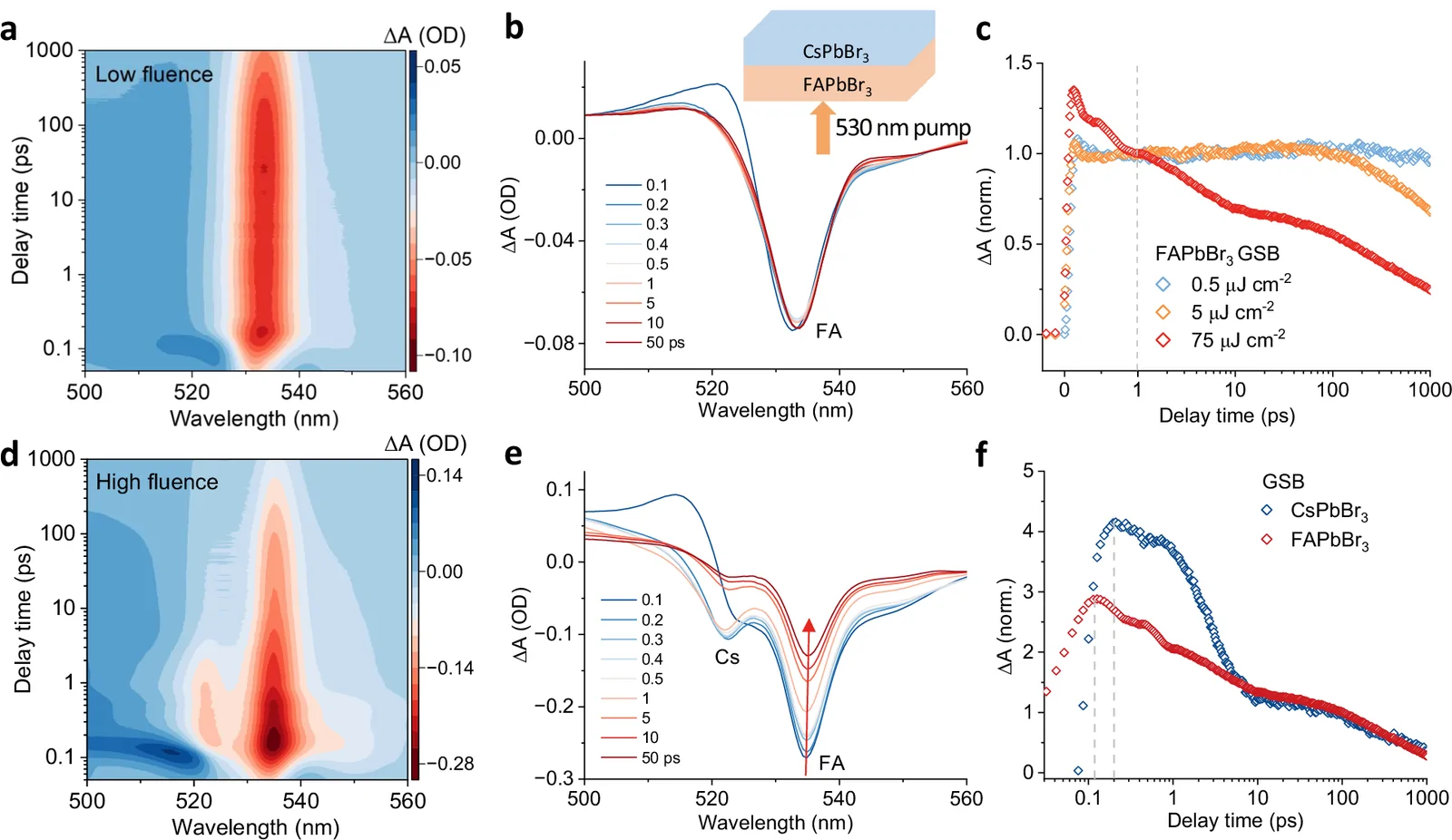
Hot carrier transfer in bilayer 3D perovskite heterojunctions. **a, b** Transient absorption (TA) spectroscopy color maps (**a**), and spectra (**b**) of laminated bilayer thin films. TA measurements were performed with pumping at FAPbBr_3_ side (530 nm, ~5 µJ cm^−2^). **c** Fluence-dependent TA kinetics obtained from FAPbBr_3_ films. **d–f** TA spectroscopy color maps (**d**), spectra (**e**), and kinetics (**f**) of laminated bilayer thin films, resonantly pumped at 530 nm with a fluence of ~75 µJ cm^−2^. The presence of CsPbBr_3_ ground state bleaching (GSB) signals under high pumping fluences proves ultrafast carrier transfer from FAPbBr_3_ to CsPbBr_3_ layers.

Having demonstrated hot carrier transfer across our high-quality bilayer perovskite heterojunctions, we next investigate hot carrier transfer mechanisms from the low bandgap semiconductor (FAPbBr_3_) to the high bandgap semiconductor (CsPbBr_3_) under resonant pumping conditions. Using a pump at 530 nm we selectively excite FAPbBr_3_, while CsPbBr_3_, with a bandgap absorption edge at approximately 516 nm, remains in the ground state. At a relatively low excitation fluence ( ~ 5 µJ cm^−2^), we only observe GSB signals at 533 nm (Fig. 5a, b), which we attribute to the band transitions of FAPbBr_3_. No signals from CsPbBr_3_ are detected. Under the quasi-type-II band alignment, a small number of holes may transfer to the CsPbBr_3_ side; however, in the absence of hot-electron transfer, these holes cannot recombine with electrons in CsPbBr_3_ and therefore do not produce a detectable GSB signal. Figure 5c presents the fluence-dependent TA kinetics obtained at the FAPbBr_3_ GSB wavelength. At high fluences, a rapid carrier recombination process is observed within the first picosecond, which we relate to Auger recombination^55–58^.

To enhance hot carrier generation from Auger or phase space filling effects, we increase the pump fluence (Fig. 5d, e). In contrast to low-fluence excitation, we observe two distinct GSB signals at 522 nm and 533 nm, which we attribute to excited carriers in CsPbBr_3_ and FAPbBr_3_ phases, respectively (Supplementary Fig. 3). Given that CsPbBr_3_ exhibits a higher bandgap than FAPbBr_3_ (Supplementary Fig. 1), and no GSB signal of CsPbBr_3_ is observed under low-fluence excitation, the emergence of CsPbBr_3_ signals at high excitation fluences provides strong evidence for hot carrier transfer from FAPbBr_3_ to CsPbBr_3_. To exclude possible contributions from nonlinear effects such as up‑conversion or two‑photon absorption, we performed wavelength‑ and fluence‑dependent transient absorption (TA) measurements on control single‑layer CsPbBr_3_ films (Supplementary Fig. 16; full mapping data are provided in Supplementary Figs. 17–22). When the pump wavelength was increased from 525 nm to 545 nm, the TA GSB signals exhibited a gradual blue shift, and the threshold fluence for the appearance of a detectable GSB increased from 7.5 µJ cm^−2^ to 150 µJ cm^−2^. This behaviour is characteristic of an anti‑Stokes up‑conversion process. Critically, even at an extremely high pump fluence of 1500 µJ cm^−2^ on the single‑layer CsPbBr_3_ film at 530 nm, the GSB signal remained below 0.012 – an order of magnitude lower than the GSB intensity observed in the laminated heterojunction under much lower excitation (Fig. 5d, e). Further, the blue‑shifted signals in the control experiment confirm the anti‑Stokes nature of the up‑conversion process, whereas in the heterojunction (Fig. 5d, e) the CsPbBr_3_ GSB appears at its expected spectral position ( ~ 520 nm) without any blue shift. This unambiguously demonstrates that the CsPbBr_3_ GSB in the heterojunction originates from carrier transfer from the FAPbBr_3_ layer, and that the carrier density transferred into CsPbBr_3_ is much higher than the carrier density achievable by direct excitation of CsPbBr_3_ alone. We further compare the GSB signals of CsPbBr_3_ and FAPbBr_3_ in laminated bilayers (Fig. 5f). The TA signal of CsPbBr_3_ exhibits a pronounced increase within the first picosecond, whereas the TA signal of FAPbBr_3_ decays rapidly. Then the two TA kinetics overlap with each other after 10 ps. These results provide a strong indication that carrier transfer from FAPbBr_3_ to CsPbBr_3_ occurs rapidly over the first ps, followed by rapid hot carrier cooling in CsPbBr_3_, to eventually recombine, completing the process within about 10 ps. Consequently, also for resonant pumping, hot carriers transfer from low bandgap to high bandgap perovskite materials in our heterojunctions, further broadening their potential for harvesting hot carriers in applications.

## Discussion

In summary, our work demonstrates ultrafast directional hot carrier transfer in bilayer perovskite heterojunctions, highlighting the critical role of differing hot carrier cooling rates in driving hot carrier migration. We identify a hot-carrier bottleneck in the hybrid FAPbBr₃ layer, characterized by a slow cooling rate, that leads to hot-carrier accumulation. Conversely, hot carriers in the all-inorganic CsPbBr₃ layer are rapidly cooled to the CBM within approximately 1 ps. This imbalance in hot carrier densities creates a driving force for transfer from the FAPbBr₃ to the CsPbBr₃ layer. By harnessing hot-carrier transfer in these heterojunctions, we demonstrate an enhancement in the population inversion of the excited states, resulting in a fivefold reduction in ASE and laser threshold compared to isolated perovskite materials. Our findings lay the groundwork for designing next-generation optoelectronic devices that leverage hot carrier dynamics to achieve higher efficiencies, improved charge extraction, and novel functionalities across applications such as photovoltaics, light-emitting devices, and lasers.

## Methods

### Materials

n-Butylammonium bromide (BABr), Dimethyl sulfoxide (DMSO), chlorobenzene (CB), and 18-Crown-6 were purchased from Sigma Aldrich. CsPbBr_3_, and CsBr were purchased from TCI. Formamidinium bromide (FABr), Methylammonium bromide (MABr), and PbBr_2_ were ordered from Greatcell Solar Materials. All chemicals were used without further purification.

### Fabrication of bilayer bulk 3D perovskite heterojunctions

The glass substrates were cut to sizes of 9 mm × 9 mm and 16 mm × 16 mm, which were then cleaned with acetone and isopropanol. After O_2_ plasma, 30 nm MgF_2_ was thermally deposited on top of the substrates.

As a general synthesis procedure of CsPbBr_3_ (90 nm) thin films, 0.2391 g CsPbBr_3_, 0.431 g BABr, or 0.3 g Crown was separately dissolved in 1 mL DMSO, which was used as stock solutions. 1 mL CsPbBr_3_ stock solution was then mixed with 57 µL BABr and 16.7 uL Crown solutions. The obtained mixture (25 uL) was spin-coated on 9 × 9 mm substrates, which have been coated with MgF_2_ (1000 rpm for 20 s and 4000 rpm for 40 s). The film was then annealed at 150 °C for 10 min.

For FAPbBr_3_ (200 nm) thin films, 0.294 g PbBr_2_, 0.1 g FABr, 0.009 g MABr, and 0.0086 g CsBr were first dissolved in 1 mL DMSO. Then the mixed solution (50 µL) was spin-coated on 16 × 16 mm substrates (1000 rpm for 20 s and 4000 rpm for 40 s). 10 s before the end of spin coating process, 150 µL CB was dropped on top of films, which were followed by 120 °C annealing for 10 min. All the film fabrications were performed in a N_2_-filled glovebox.

To fabricate laminated bilayer bulk perovskite heterojunctions, CsPbBr_3_ and FAPbBr_3_ films were placed face-to-face in a hot embossing machine. Then the substrates were heated up to 120 °C in an N_2_ atmosphere. A 10,000 N force was applied on top of substrates ( ~ 120 MPa) for 5 min. After that, the substrates were cooled down to 50 °C with the pressure released. To distribute the force homogeneously onto the substrates during lamination, two aluminum foils were placed between the substrates and embossing plates (top and bottom). After lamination, UV glue was used to seal the lamination samples along the edges for long-term optical measurements.

### Fabrication of bilayer perovskite heterojunction lasers

Commercial DBR mirrors (Knight optical, 552FDN50) were used here for VCSEL fabrication. After O_2_ plasma cleaning, different thicknesses of MgF_2_ (from 10 nm to 60 nm) were thermally deposited on top of DBR mirrors to control the cavity thickness. CsPbBr_3_ films and FAPbBr_3_ films were separately deposited on 9 × 9 mm and 16 × 16 mm DBR mirrors, which were then followed by the same laminated process (120 °C, 100 MPa) as the glass substrates. The final assembled bilayer heterojunction laser devices were also sealed by UV glue before optical measurements.

### General structural and optical measurements

The crystal structures of the perovskite films were measured by XRD (Bruker D2Phaser system) with Cu-Kα radiation (λ = 1.5405 Å) in Bragg–Brentano configuration using a LynxEye detector. Field emission top-view and cross-section SEM images of perovskite films were taken with a ZEISS Supra60 VP scanning electron microscope. SEM cross‑sections show thicknesses of ~90 nm for single-layer CsPbBr_3_, ~200 nm for single-layer FAPbBr_3_, and ~250 nm for the laminated bilayer. For cross‑sectional SEM imaging of the laminated bilayer, the CsPbBr_3_ and FAPbBr_3_ films were deposited on glass and ITO, respectively. After lamination, the two substrates were separated; the bilayer adhered preferentially to the glass side, enabling clean cross‑sectional imaging.

Absorption spectra were recorded using the PerkinElmer Spectrophotometer (Lambda 1050 UV/Vis/NIR) equipped with an integrating sphere. The PL and lasing measurements were performed using a nanosecond pulsed laser (INNOLAS, MOPA 25, 355 nm, 1 ns). The emission spectra were recorded by a SpectraPro HRS-500 spectrometer equipped with a Princeton Instruments PIXIS:400BR CCD with a spectra resolution <0.05 nm. Variable-neutral density filters (ND filters) were used to tune the laser power during measurements, and a LabMax TOP (Coherent) power meter was used to record real-time excitation powers. For angle-resolved PL measurements, a 355 nm nanosecond pulsed laser (CryLas, FTSS-355-Q, 1.3 ns, 355 nm) was used to pump the samples, and a fiber-connected Ocean Optics spectrometer was employed to detect PL signals. The goniometer was used to control the PL detection angle.

### Time resolved photoluminescence

A 405 nm femtosecond laser (Light Conversion, Pharos/Orpheus, 260 fs, 1 kHz) was adopted as the excitation laser. The PL signal was recorded by a streak camera (Hamamatsu Universal C10910) equipped with a spectrometer (Andor Kymera 328i).

### Confocal photoluminescence mapping

Confocal PL measurements were performed on the customized confocal microscope module (Neaspec, Attocube GmbH) together with an EMCCD camera-based spectrometer (iDUS + Kymera, Andor, Oxford Instruments). The sample was fixed on the table at the 3-axis piezo translation stage. The sample was excited through the long working distance objective with the 460 nm of CW laser beam (C-Wave, Huebner Photonics GmbH). The same objective was used for the collection of PL in reflection geometry. After collection, the PL was spatially filtered with a pinhole (to preserve the degree of polarization of the PL), spectrally filtered from the excitation laser, and finally detected with the spectrometer.

### Photoluminescence quantum yield (PLQY)

A 405 nm CW laser (Thorlabs) was used to excite the samples. The measurements were performed inside an integrating sphere in ambient conditions. An optical fiber collected the emission from the samples and was directed to the spectrometers (QE65 Pro from Ocean Optics from Avantes). The spectral response was calibrated using a calibration lamp (HL-3plus-INT-Cal from Ocean Optics). The PLQY value was determined by following the method described by de Mello et al. ^59^.

### TA spectroscopy measurements

Room-temperature TA measurements were performed by a commercial TA setup (Ultrafast Systems, HELIOS). We globally fitted the decay kinetic traces of CsPbBr_3_ and FAPbBr_3_ (data in Fig. 3c) to extract hot carrier transfer components (CsPbBr_3_: hot carrier gain/bleach, FAPbBr_3_: hot carrier transfer out). We use a comprehensive kinetic model that includes hot-carrier cooling, hot-carrier transfer (τ_transfer_), and biexponential decay components (τ_fast_, τ_slow_) for both phases with corresponding branching ratios. The model also accounts for an instrument response function (IRF, deconvolved during fitting). All parameters were shared globally across both traces, and the fit was performed by least-squares minimization with regularization on the fast components. The fitted hot carrier transfer constant and carrier decay constant were listed in Supplementary Table 2.

## Supplementary information

SUPPLEMENTARY INFO

Transparent Peer Review file

## Data Availability

The data files for the results presented in this study have been deposited in the Zenodo database under accession code https://doi.org/10.5281/zenodo.21496582.
