# Supplementary material for: Ultrafast directional hot carrier transfer in bilayer perovskite heterojunctions
Source: Nat Commun. 2026 Sep 7;17:9555. doi: 10.1038/s41467-026-76790-z (PMC13550544; doi:10.1038/s41467-026-76790-z)
Supplement: Supplementary file 1 [file 41467_2026_76790_MOESM1_ESM.pdf]

Supplementary Information

Ultrafast directional hot carrier transfer in bilayer perovskite heterojunctions

Shangpu Liu,[1,2,3] Changhao Gao,[3] Mohammad Gholipoor,[1,2] Tonghan Zhao,[1,2] Andrii Shcherbakov,[3] Zhiwei Lei,[4] Pirmin Tischler,[2] Yiqin Xie,[5] Nils W. Rosemann,[2] Uli Lemmer,[2] Yang Li,[1,2,4]* Felix Deschler[3]*, Ulrich W. Paetzold,[1,2]*

[1]Institute of Microstructure Technology, Karlsruhe Institute of Technology, Hermann-von-Helmholtz-Platz 1, 76344 Eggenstein-Leopoldshafen, Germany

[2]Light Technology Institute, Karlsruhe Institute of Technology, Engesserstraße 13, 76131 Karlsruhe, Germany

[3]Physikalisch-Chemisches Institut, Universität Heidelberg, Im Neuenheimer Feld 229, 69120 Heidelberg, Germany

[4]College of Materials Science and Engineering & Engineering Research Center of Alternative Energy Materials & Devices of Ministry of Education, Sichuan University, Chengdu 610065, China

[5]Physics Department and HK Institute of Quantum Science & Technology, The University of Hong Kong, Pokfulam road, Hong Kong, China

*Corresponding author. Email: yangli2024@scu.edu.cn, deschler@uni-heidelberg.de, ulrich.paetzold@kit.edu

Supplementary Methods

Extraction of optical gain coefficient

The optical gain coefficient *g* is a core parameter for evaluating the performance of gain media, which quantifies the increase in light intensity per unit length as light waves propagate in the medium. Variable strip length (VSL) or variable pump intensity (VPI) methods are commonly used to extract the optical gain coefficient *(1-2)*. Here, we quantitatively extracted the net optical gain coefficients directly from our transient absorption data using the well-established relationship:

$$g_{th} = \frac{10^{-(A_0+\Delta A)} - 1}{d}$$

Where d is the thickness of the film, A_0 is the steady state absorbance, ΔA is the TA signals at the lasing threshold. The threshold carrier density (N_{th}) was determined from the fluence-dependent PL data. The calculated threshold carrier density and optical gain coefficient values were listed in Supplementary Table. 1.

Modeling of laser rate equation for the bilayer perovskite heterojunctions

To model the stimulate emission process in the bilayer perovskite heterojunctions, the following set of modified laser rate equations including the term for hot carrier transfer are used:

$$\frac{dn_{\mathrm{Cs}}}{dt} = G_{\mathrm{Cs}}(t) + \left(\frac{dn_{\mathrm{Cs}}}{dt}\right)_{\mathrm{trans}} - k_1 n_{\mathrm{Cs}} - k_2 n_{\mathrm{Cs}}^2 - k_3 n_{\mathrm{Cs}}^3 + (n_{\mathrm{Cs}} - n_{\mathrm{tr\_Cs}})\delta_{\mathrm{st}} v_{\mathrm{g}} n_{\mathrm{p\_Cs}} \tag{1}$$

$$\frac{dn_{\mathrm{p\_Cs}}}{dt} = \Gamma \beta_{\mathrm{sp}} k_2 n_{\mathrm{Cs}}^2 + \Gamma(n_{\mathrm{Cs}} - n_{\mathrm{tr\_Cs}})\delta_{\mathrm{st}} v_{\mathrm{g}} n_{\mathrm{p\_Cs}} - \frac{n_{\mathrm{p\_Cs}}}{\tau_{\mathrm{p\_Cs}}} \tag{2}$$

$$\frac{dn_{\mathrm{FA}}}{dt} = G_{\mathrm{FA}}(t) - \frac{n_{\mathrm{FA}}}{\tau_{\mathrm{trans}}} - \frac{n_{\mathrm{FA}}}{\tau_{\mathrm{FA}}} \tag{3}$$

where n, n_{p}, k_1, k_2, k_3, n_{tr}, σ_{st}, v_{g}, Γ, β_{sp}, τ_{p}, τ_{trans}, and τ_{FA} are the carrier density, photon density, 1st rate constant (5.6×10^8 s^{-1}) 2nd rate constant (1×10^{-9} cm^3 s^{-1}), 3rd rate constant (0.6×10^{-29} cm^6 s^{-1}), transparency carrier density (10^{18} cm^{-3}), stimulated emission cross-section (10^{-16} cm^2), group velocity (5.6×10^9 cm s^{-1}), modal confinement factor (0.56), spontaneous emission coupling factor (10^{-4}), photon escape time (100 ps), hot carrier transfer time constant (1 ps), and carrier lifetime of $FAPbBr_3$ perovskite, respectively. *G*(*t*) is the Gaussian generation function (FWHM = 270 fs), and the integral of *G*(*t*) equals the initial carrier density

($\int G_{Cs}(t)dt + \int G_{FA}(t)dt = N_0$), which is varied from 10^{17} cm^{-3} to 10^{20} cm^{-3} during the modeling.

For the modeling of single layer perovskite, the generation laser rate equations (eqn. 1 and eqn. 2) are used with neglecting the hot carrier transfer term in eqn. 1. Upon numerically solving the rate equations, the time profile of photon density and L_{in}- L_{out} characteristics can be obtained (Supplementary Figure 8). Then effect of hot carrier transfer in the bilayer perovskite heterojunction to the lasing performance can be theoretically evaluated via comparing the lasing threshold between the single layer perovskite and heterojunction.

Supplementary Tables

Supplementary Table 1. Optical gain coefficient values for $CsPbBr_3$ films before and after lamination process.

Samples	Threshold carrier density N_{th} (cm^{-3})	Optical gain coefficient g_{th} (cm^{-1})
$CsPbBr_3$ Only	~2.6×10^{18}	**~255**
Laminated $CsPbBr_3$/$FAPbBr_3$ heterojunction	~7.3×10^{17}	**~712**

Supplementary Table 2. Fitting parameters of the hot carrier transfer in $CsPbBr_3$/$FAPbBr_3$ laminated heterojunctions.

Phases	$\tau_{transfer}$	τ_{fast}	τ_{slow}
Laminated $FAPbBr_3$	**1.2 ± 0.7 ps**	258 ± 1 ps (Amplitude 0.14)	~984 ± 1 ps (Amplitude 0.86)
Laminated $CsPbBr_3$	**9.8 ± 0.6 ps**	6.3 ± 0.6 ps (Amplitude 0.94)	~4890 ± 5 ps (Amplitude 0.06)

Supplementary Table 3. Summary of the recent reported state-of-the-art lasers.

Materials	Cavity	Pump (nm)	Emission (nm)	Pulse	Threshold	T (K)	Reference
$MAPbI_3$ film	DFB	445	790	20 ns	5 KW cm^{-2}	160	*Nano Lett.* 16, 7, 4624 (2026)
$CsPbBr_3$ QD film	VCSEL	400	505	5 ns	900 μJ cm^{-2}	RT	*Adv. Funct. Mater.* 27, 1605088 (2017)
$CsPbBr_3$ QD film	VCSEL	355	523	5 ns	98 μJ cm^{-2}	RT	*ACS Photonics* 4, 2281 (2017)
$CsPbBr_3$ film	DFB	355	539	0.3 ns	7.2 μJ cm^{-2}	RT	*Adv. Mater.* 31, 1903717 (2019)
N2F8/P2F8 quasi 2D film	DFB	488<br>337	555/553<br>559/52	CW<br>3 ns	0.059/0.045 KW cm^{-2}<br>4.7/32.8 μJ cm^{-2}	RT	*Nature* 585, 53 (2020)
$CsPbBr_3$ microcrystals	plasmonic lasers	480	540	4 ns	0.2 mJ cm^{-2}	RT	*Sci. Adv.*, 7, abf3362 (2021)
Quasi-2D/3D heterostructure	VCSEL	355	538	1.1 ns	46 μJ cm^{-2}	RT	*Adv. Funct. Mater.*, 32, 2200772 (2022)
Dion–Jacobson quasi 2D film	DFB	532	532-535	0.5 ns	5.5/8.9 μJ cm^{-2}	RT	*Sci. Adv.*, 9, eadj3476 (2023)
$MAPbI_3$ film	DFB	532	784	3 ns	100 mJ cm^{-2}	RT	*ACS Photonics*, 10, 1591 (2023)
$NMA_2FA_8Pb_8Br_{26}$ quasi-2D film	DFB	337	537	0.8 ns	1 μJ cm^{-2}	RT	*Adv. Funct. Mater.*, 33, 2301794 (2023)
Quasi-2D double heterostructure film	VCSEL	355	531	8 ns	53.7 μJ cm^{-2}	RT	*Nano Lett.*, 24, 6010 (2024)
$(FAPbI_3)_{0.95}(MAPbBr_3)_{0.05}$ film	DFB	532	787–830	2.3 ns	130 μJ cm^{-2}	RT	*Adv. Opt. Mater.*, 12, 2302496 (2024)

$Cs_{0.1}FA_{0.9}PbI_{2.855}Br_{0.145}$ LED	ASE	447	790	1000 ns	3.8 KW cm^{-2}	70	*Nat. Photon., 18, 132 (2024)*
$FA_{0.1}MA_{0.9}PbBr_3$ single-crystals	VCSEL	405	551	CW	0.4 W cm^{-2}	RT	*Sci. Adv.*, 11, eadr8826 (2025).
$CsPbBr_3$ nanocrystals	Fabry-Pérot cavity	355	530	7 ns	155 mJ cm^{-2}	RT	*Sci. Adv.*, 11, eadq9002(2025).
$CsPb(Cl_{1-x}Br_x)_3$ nanocrystals	In-glass structures	405	511	CW	57.2 mW cm^{-2}	83	*Sci. Adv.*, 12, eadz8460 (2026).
$CsPbBr_3/FAPbBr_3$ film	VCSEL	355	553	1 ns	1.4 μJ cm^{-2}	RT	**This work**

Supplementary Figures

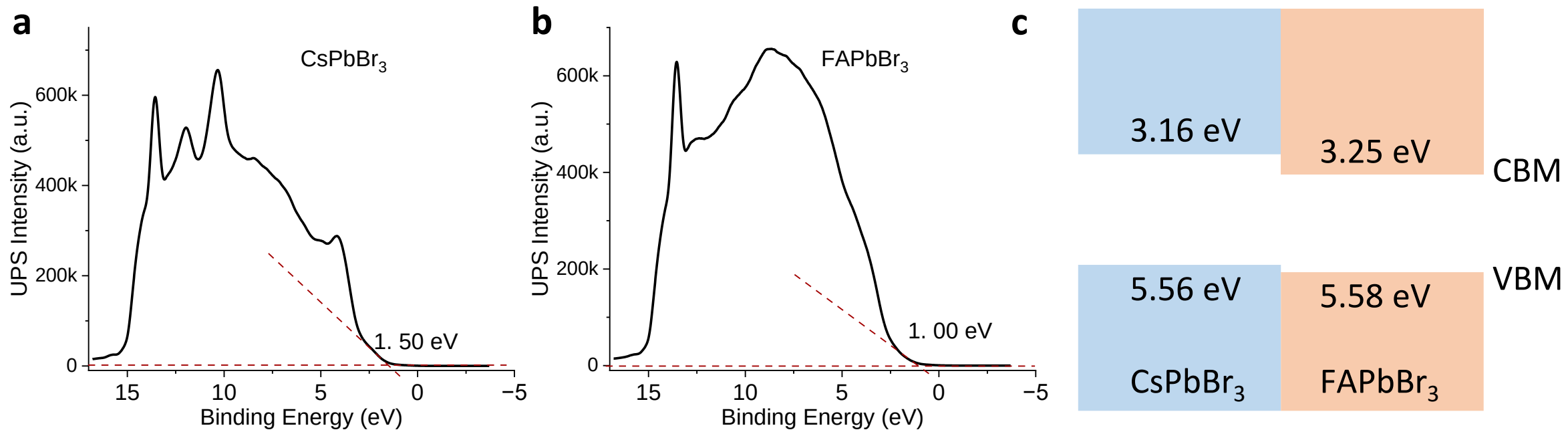

Supplementary Figure 1. Band alignment of laminated heterojunctions. a, b, UPS measurements of $CsPbBr_3$ (a) and $FAPbBr_3$ (b). **c,** Schematic illustration of band alignment of the lamination bilayer perovskite heterojunctions.

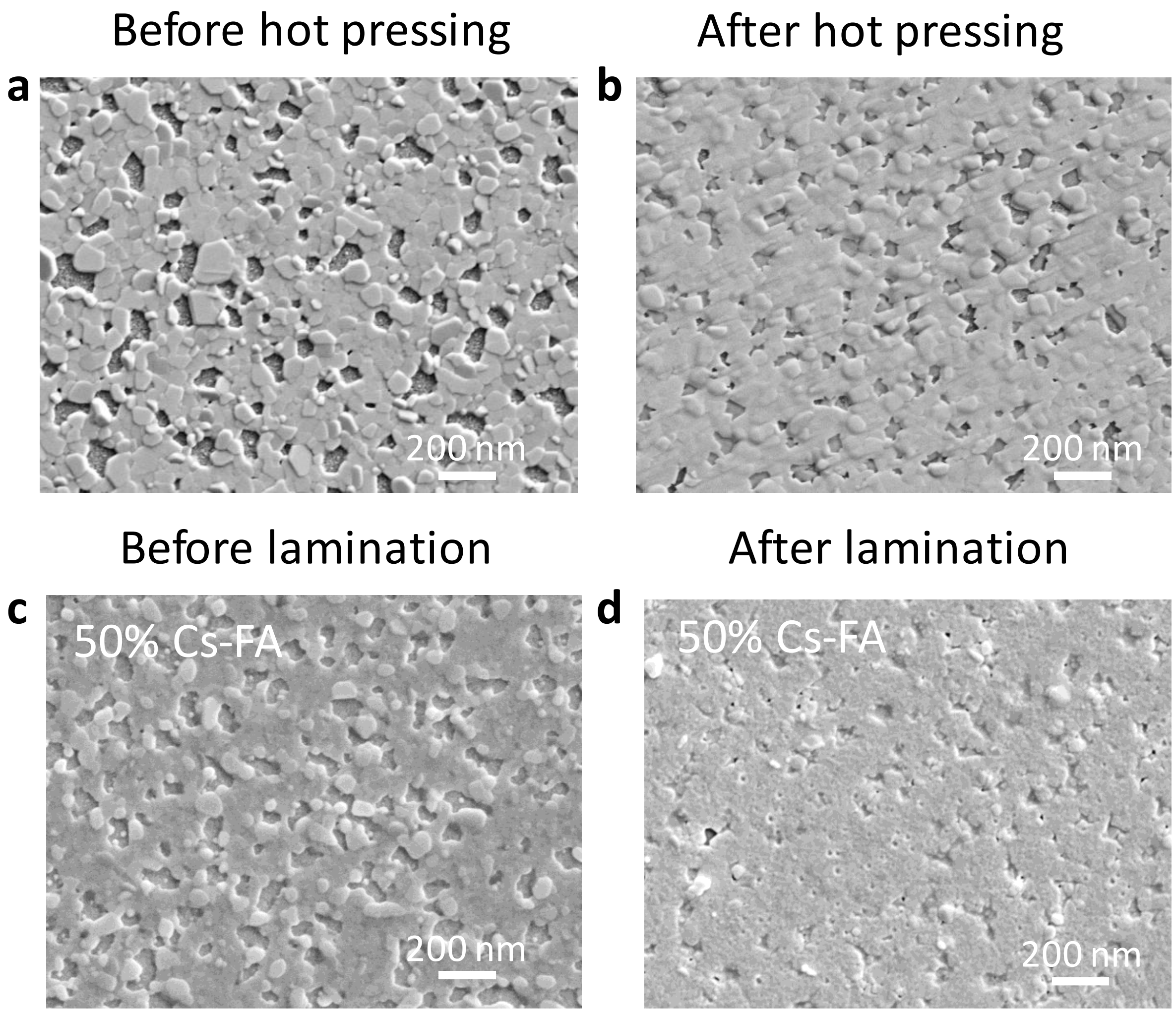

Supplementary Figure 2. Top-view SEM images of $CsPbBr_3$ films under different processing conditions. a, b, $CsPbBr_3$ before and after hot pressing process. A clean silicon wafer was used to press on top of $CsPbBr_3$ films under the same pressure and temperature conditions as lamination (i.e., without a second perovskite layer). Hot pressing reduces surface roughness, but the improvement is moderate. c, d, $CsPbBr_3$ film with reduced thickness (50% of the original spin-coating solution concentration) before after lamination with a $FAPbBr_3$ (reduced thickness, 50% of the original spin-coating solution concentration). The morphological change is less pronounced compared to the standard-thickness bilayer, indicating that film thickness plays a critical role in the recrystallization process.

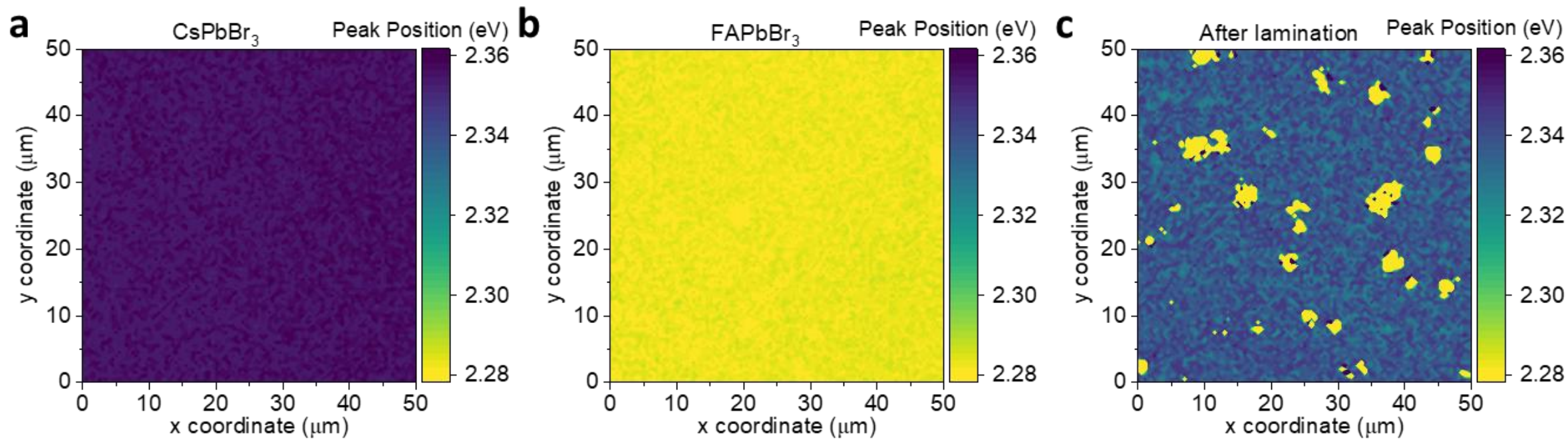

Supplementary Figure 3. **Confocal PL maps of perovskite films. a-c,** 2D PL images were obtained from $CsPbBr_3$ (a), $FAPbBr_3$ (b), and $CsPbBr_3$ films after lamination process (c). Samples were excited with a 460 nm continuous wave laser.

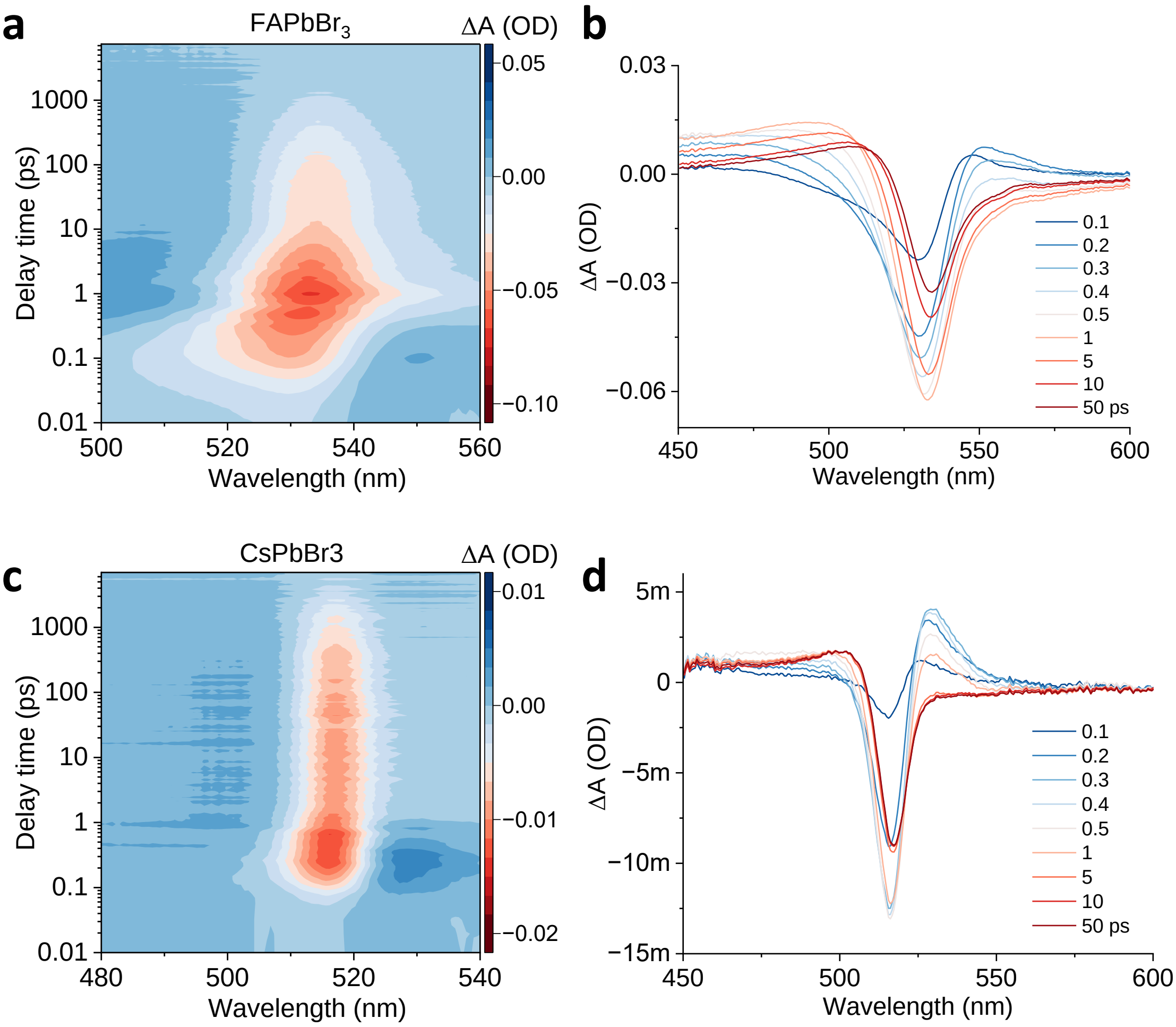

Supplementary Figure 4. Transient absorption (TA) spectra of perovskite films. **a, b,** TA maps (a) and spectra (b) obtained from $FAPbBr_3$ films. **c, d,** TA maps (c) and spectra (d) collected from $CsPbBr_3$ films. A 400 nm pulsed laser with a pump fluence of 25 μJ cm^{-2} was used here to excite the samples.

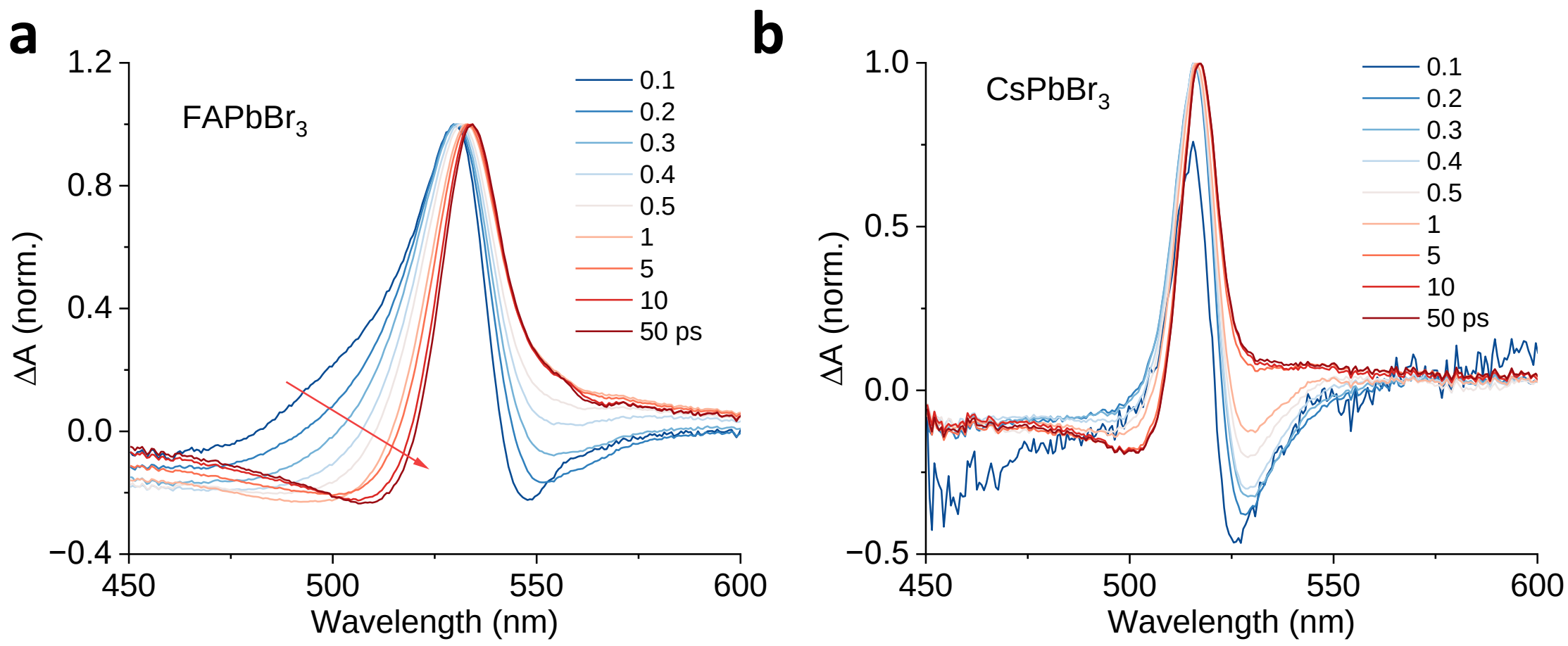

Supplementary Figure 5. Normalized TA spectra, obtained from $FAPbBr_3$ (a) and $CsPbBr_3$ (b) films. A 400 nm pulsed laser with a pump fluence of 25 μJ cm^{-2} was used here to excite the samples.

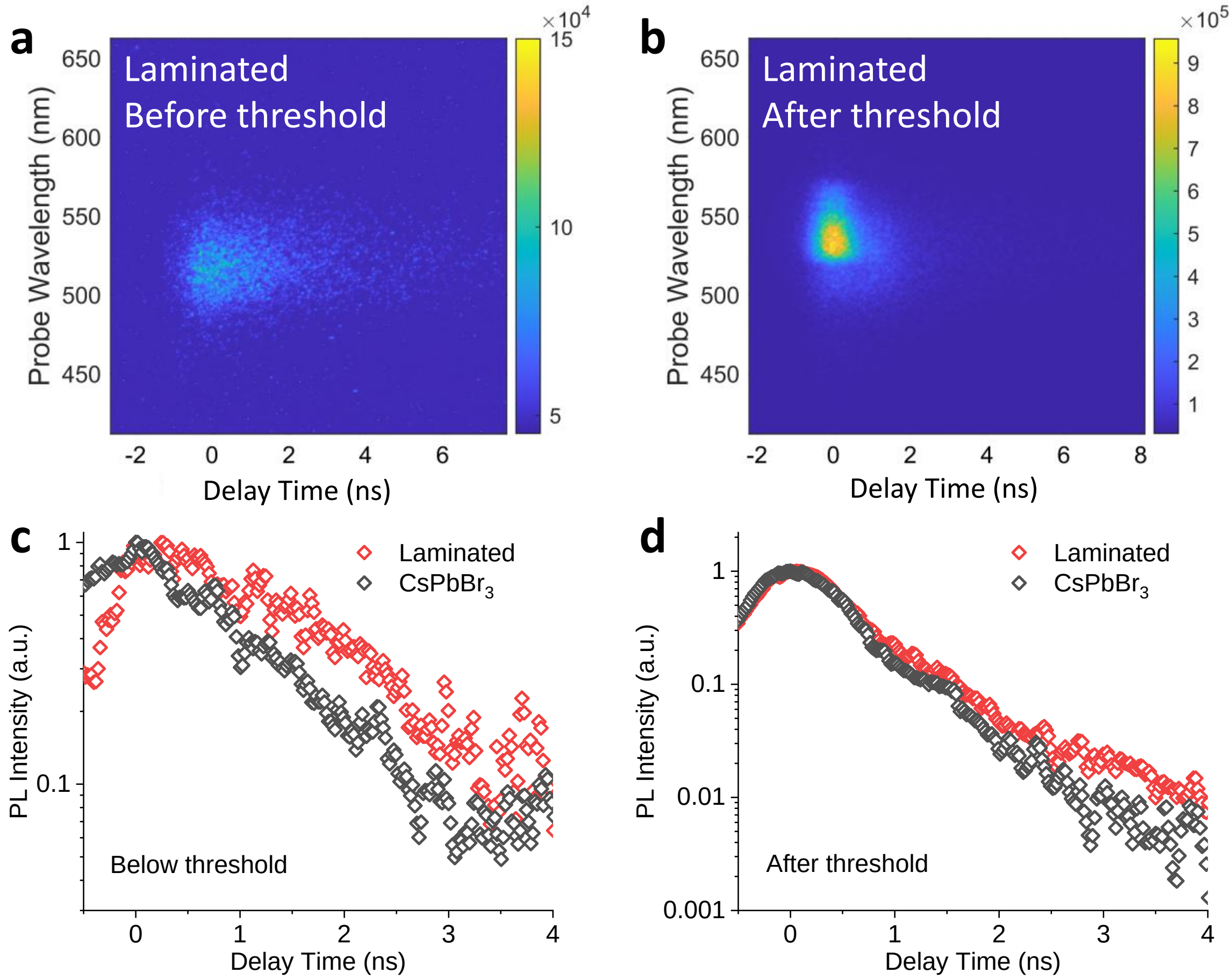

Supplementary Figure 6. Time resolved PL spectra of laminated samples. a, b, Time resolved PL mapping obtained from laminated $CsPbBr_3$ samples before (a) and after ASE threshold (b). **c, d,** Comparison of PL lifetime of laminated $CsPbBr_3$ and individual $CsPbBr_3$ films before (c) and after ASE threshold (d).

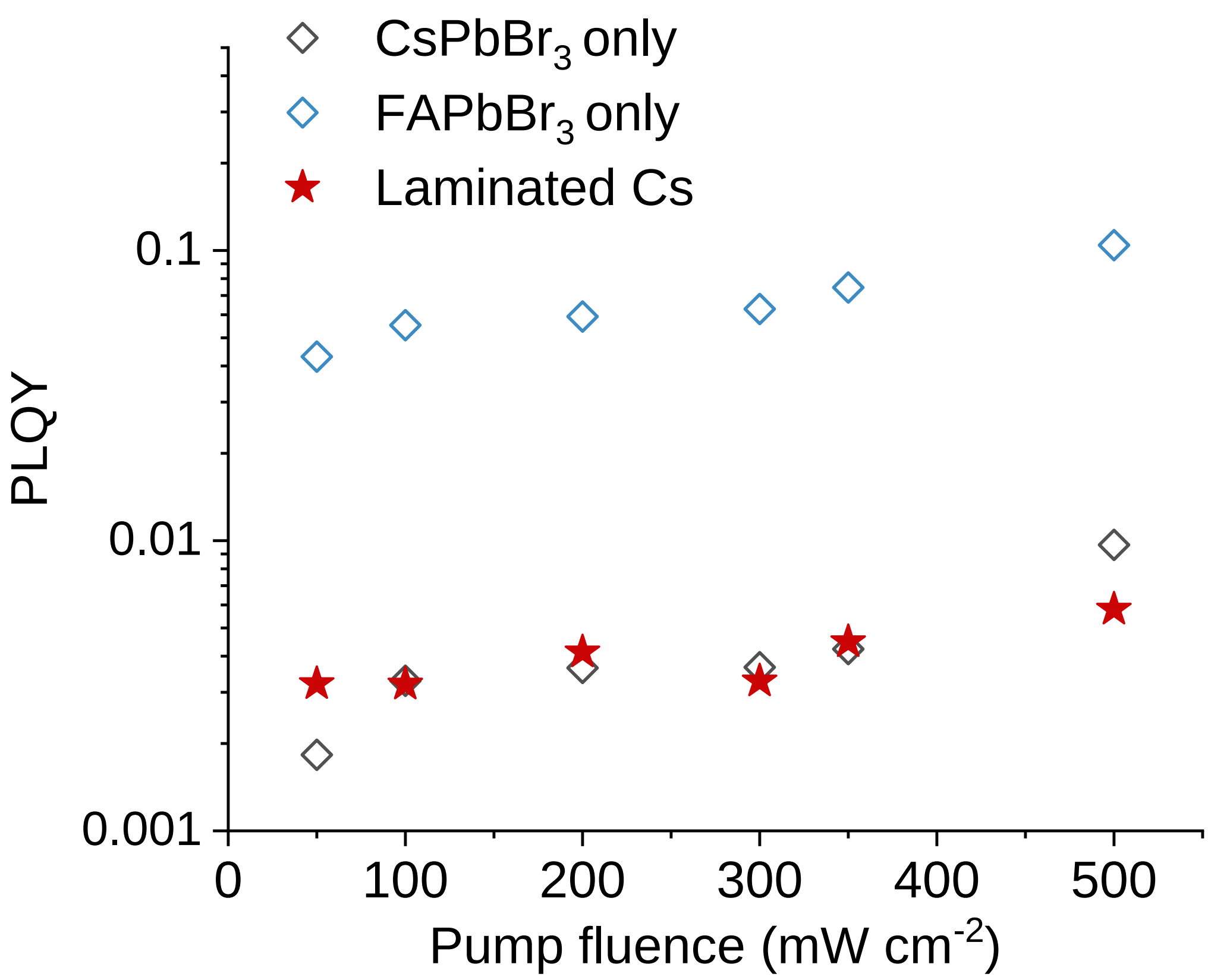

Supplementary Figure 7. Comparison of photoluminescence quantum yield (PLQY) results before and after lamination process. The samples were excited with a 400 nm continuous wave laser, and a ND filter wheel was used to control the excitation fluences. $FAPbBr_3$ films exhibit a PLQY that is at least ten times higher than that of $CsPbBr_3$ films at all pump powers. Importantly, after the lamination process, the PLQY of the $CsPbBr_3$ films remains almost unchanged compared to their pre-lamination values, further confirming that the defect density is not altered by lamination.

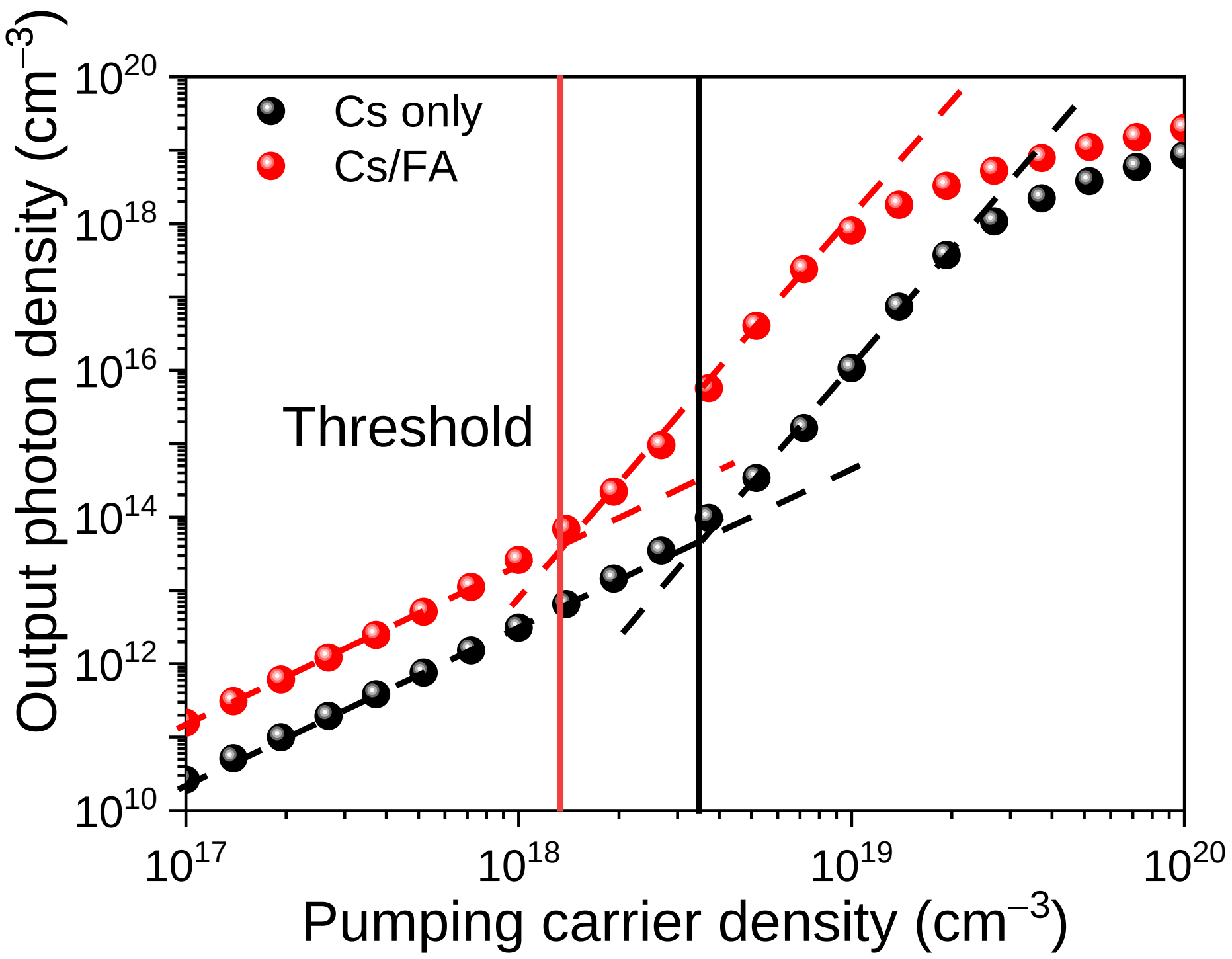

Supplementary Figure 8. Modelled L_{pump}-L_{out} characteristics for $CsPbBr_3$ and $CsPbBr_3/FAPbBr_3$ perovskite heterojunction via numerically solving the laser rate equations. The modeling results show a significant reduction in lasing threshold (3.7×10^{18} cm^{-3} for $CsPbBr_3$ and 1.2×10^{18} cm^{-3} for $CsPbBr_3/FAPbBr_3$) via introducing a hot carrier transfer term into the laser rate equations.

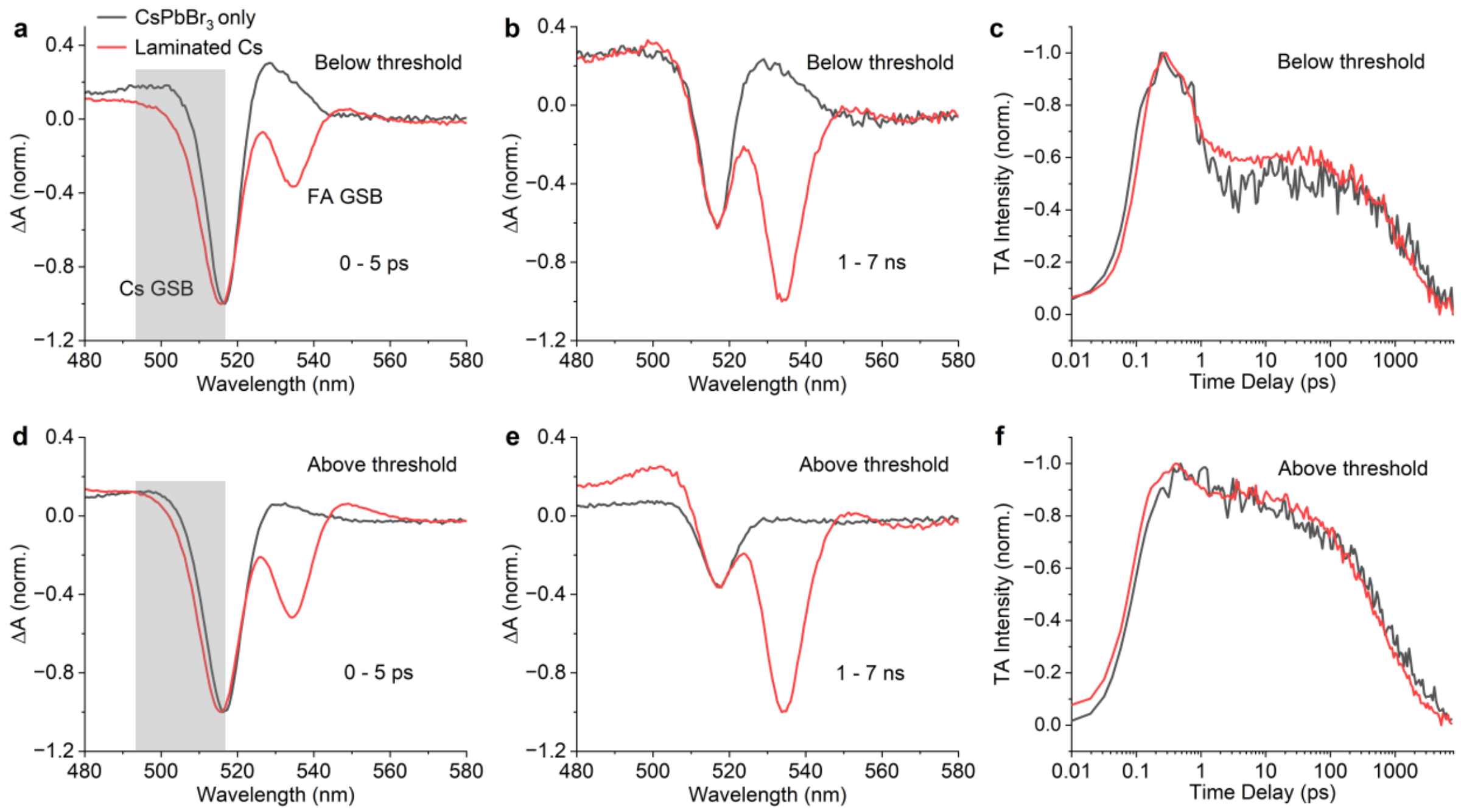

Supplementary Figure 9. Comparison of transient absorption spectra before and after lamination process. a-c, Low carrier density generation regime (below ASE threshold), TA spectra (a, 0-5 ps; b, 1-7 ns) and kinetics of $CsPbBr_3$ GSB (c) before and after lamination. d-f, High carrier density generation regime (above ASE threshold), TA spectra (a, 0-5 ps; b, 1-7 ns) and kinetics of $CsPbBr_3$ (c) before and after lamination. Notably, at equivalent carrier generation densities, the $CsPbBr_3$ GSB decay kinetics exhibit nearly identical behavior before and after lamination, indicating that the defect density remains essentially unchanged by the lamination process.

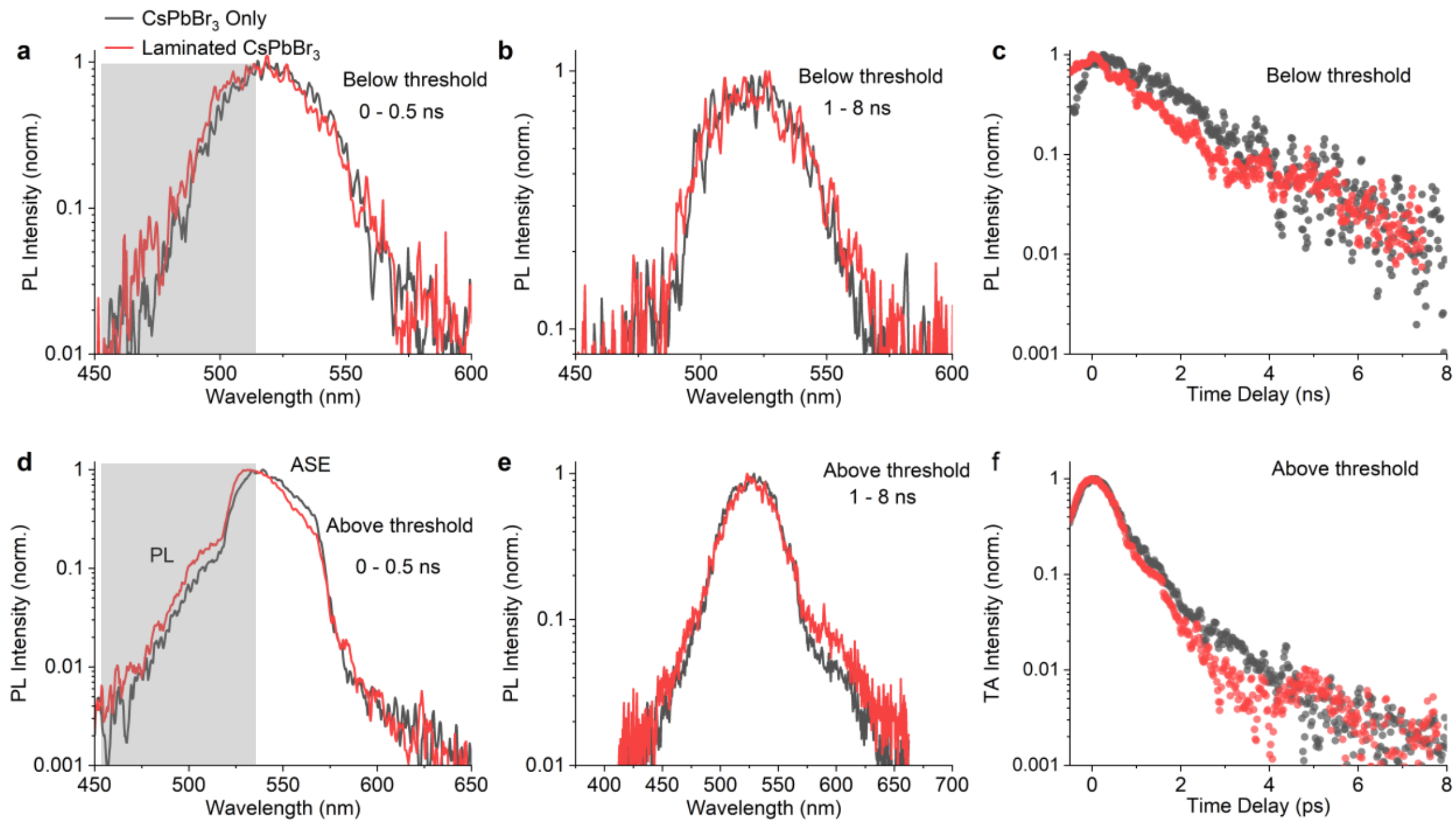

Supplementary Figure 10. Comparison of time-resolved PL spectra before and after lamination process. a-c, With low excitation fluence (below ASE threshold), PL spectra (a, 0-5 ns; b, 1-8 ns) and decay kinetics of $CsPbBr_3$ (c) before and after lamination. d-f, With high excitation fluence (above ASE threshold), PL spectra (a, 0-5 ns; b, 1-8 ns) and decay kinetics of $CsPbBr_3$ (c) before and after lamination. Time resolved PL spectra were obtained by streak camera. After lamination, the $CsPbBr_3$ PL decay exhibits a prolonged lifetime during the initial 4 ns, which we attribute to fast hot-carrier transfer from the $FAPbBr_3$ layer. Beyond 4 ns, the decay kinetics before and after lamination become nearly overlapped, confirming that the lamination process does not alter the density of long-lived trap states.

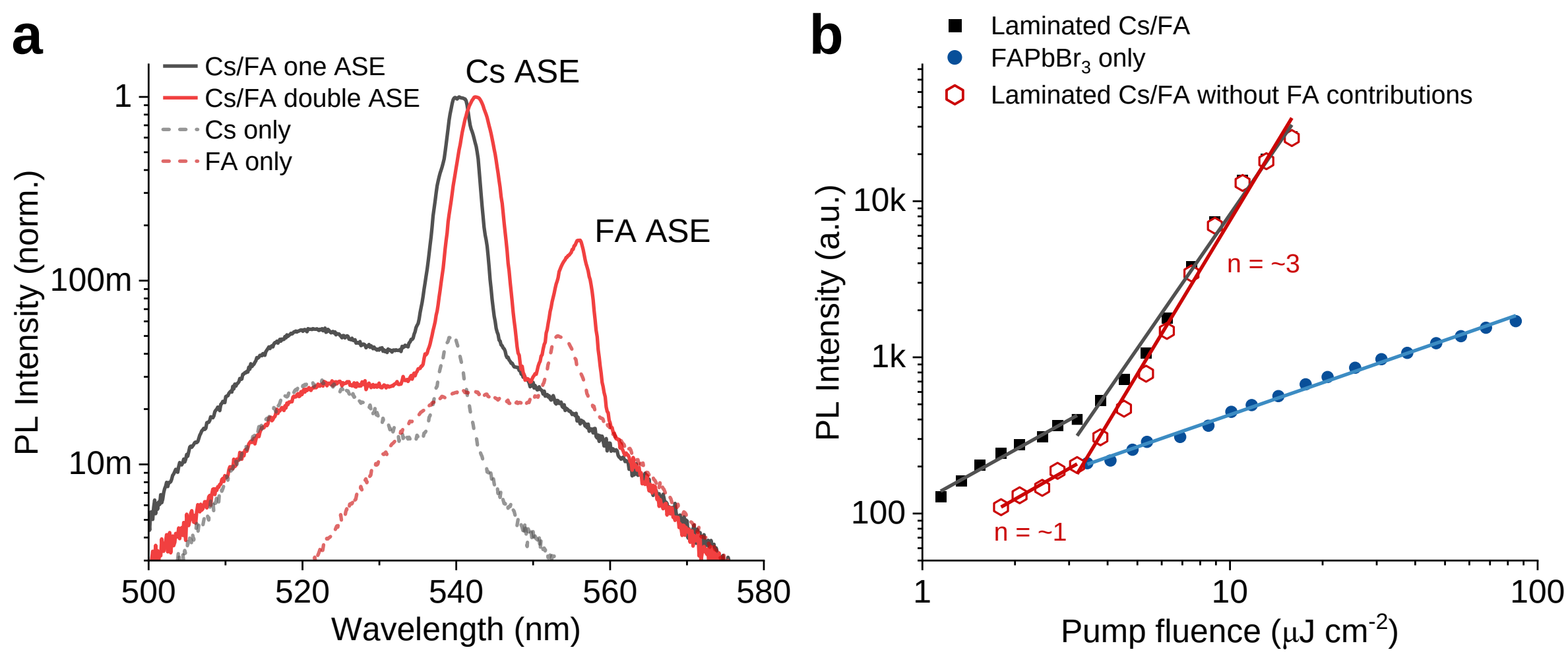

Supplementary Figure 11. Comparison of ASE signals before and after lamination process. a, PL spectra of the laminated bilayer, pure $CsPbBr_3$, and pure $FAPbBr_3$ films under high-fluence excitation (355 nm, 1 ns pulse width, 1 kHz repetition rate). The laminated heterojunction exhibits two distinct ASE peaks, corresponding to ASE from $CsPbBr_3$ (~540 nm) and $FAPbBr_3$ (~556 nm). b, Fluence-dependent PL intensity at 540 nm for the laminated heterojunction (black squares) and a pure $FAPbBr_3$ film (blue dots). Red hollow dots represent the heterojunction signal after subtracting the contribution of the $FAPbBr_3$ layer at 540 nm. After removing the $FAPbBr_3$ background, the laminated heterojunction also shows a transition from a linear increase (below threshold) to a superlinear increase (above threshold) with a superlinear slope factor of ~3, confirming the onset of ASE originating from carriers transferred into the $CsPbBr_3$ layer. The threshold fluence remains unchanged compared to the original (unsubtracted) data.

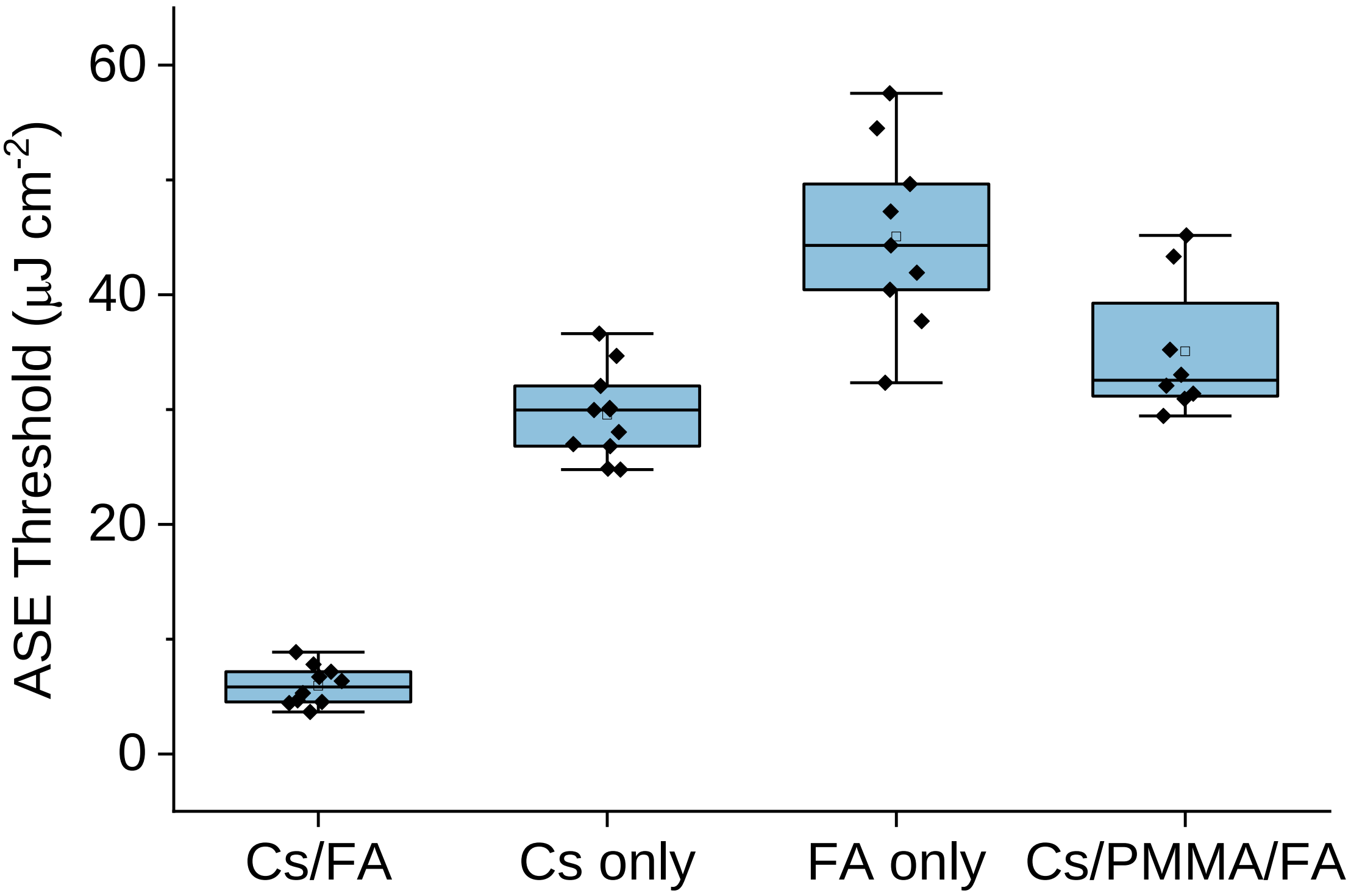

Supplementary Figure 12. Comparison of amplified spontaneous emission (ASE) thresholds. ASE thresholds were measured for the laminated $CsPbBr_3/FAPbBr_3$ heterojunction, single-layer $CsPbBr_3$, single-layer $FAPbBr_3$, and a control sample with a PMMA interlayer inserted between the two perovskite layers during the lamination process. Each condition was measured on at least five devices (individual data points shown as solid squares). A 355 nm nanosecond pulsed laser was used for excitation.

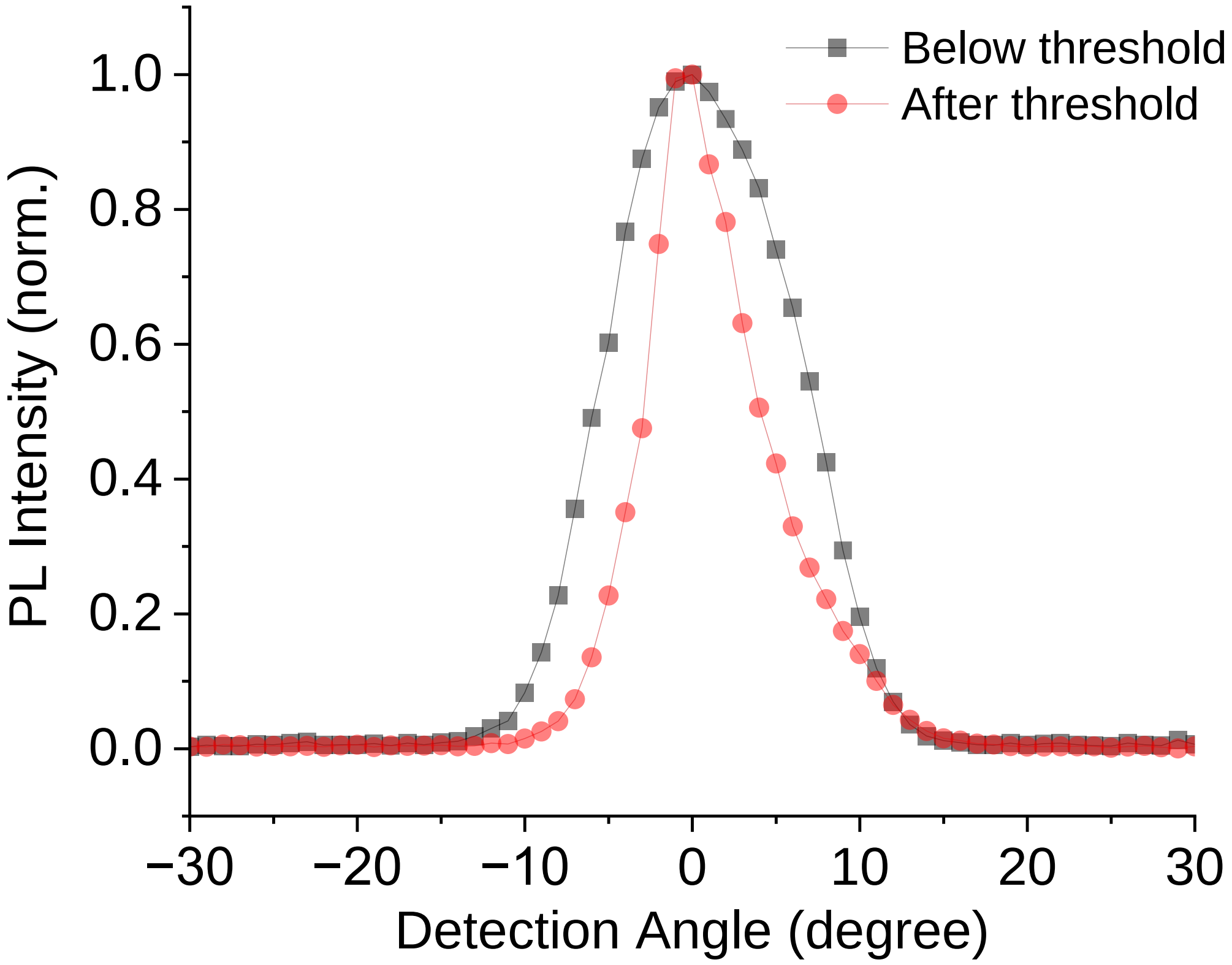

Supplementary Figure 13. Angle resolved PL intensity of laminated VCSELs. The data are collected from PL position at 542 nm.

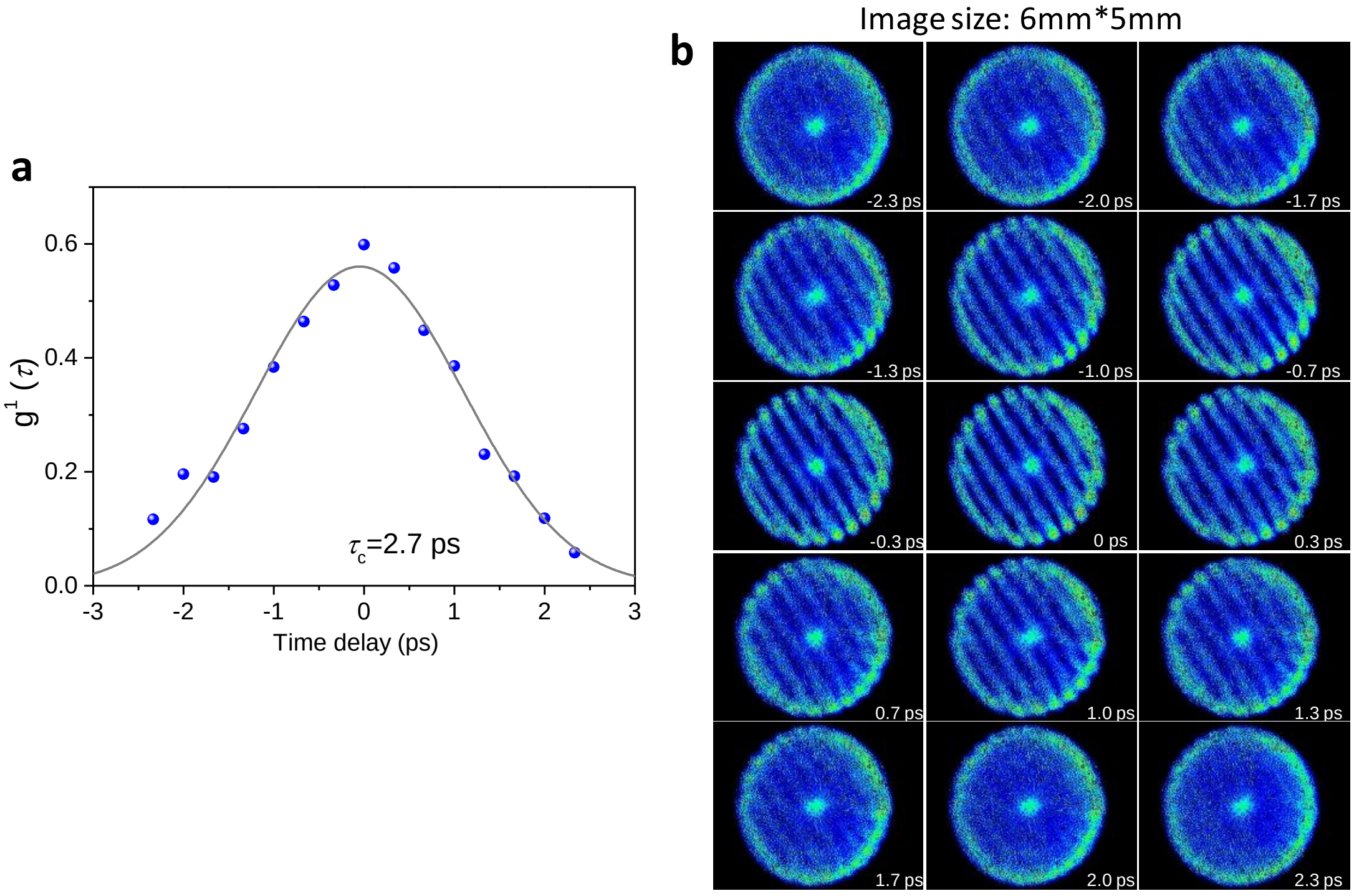

Supplementary Figure 14. Temporal coherence of the laser emission from the laminated heterojunction VCSEL. a, First-order coherence function $g^1(\tau)$ measured above the lasing threshold using a Michelson interferometer. The data points are obtained from the envelope of interference fringes as a function of time delay. The solid line represents a Gaussian fit to the coherence envelope, yielding a coherence time of 2.7 ps. The solid line represents a Gaussian fit to the data. b, Representative Michelson interference pattern at time delay from -2.3 ps to 2.3 ps. The high-contrast fringes directly demonstrate the temporal coherence of the laser emission.

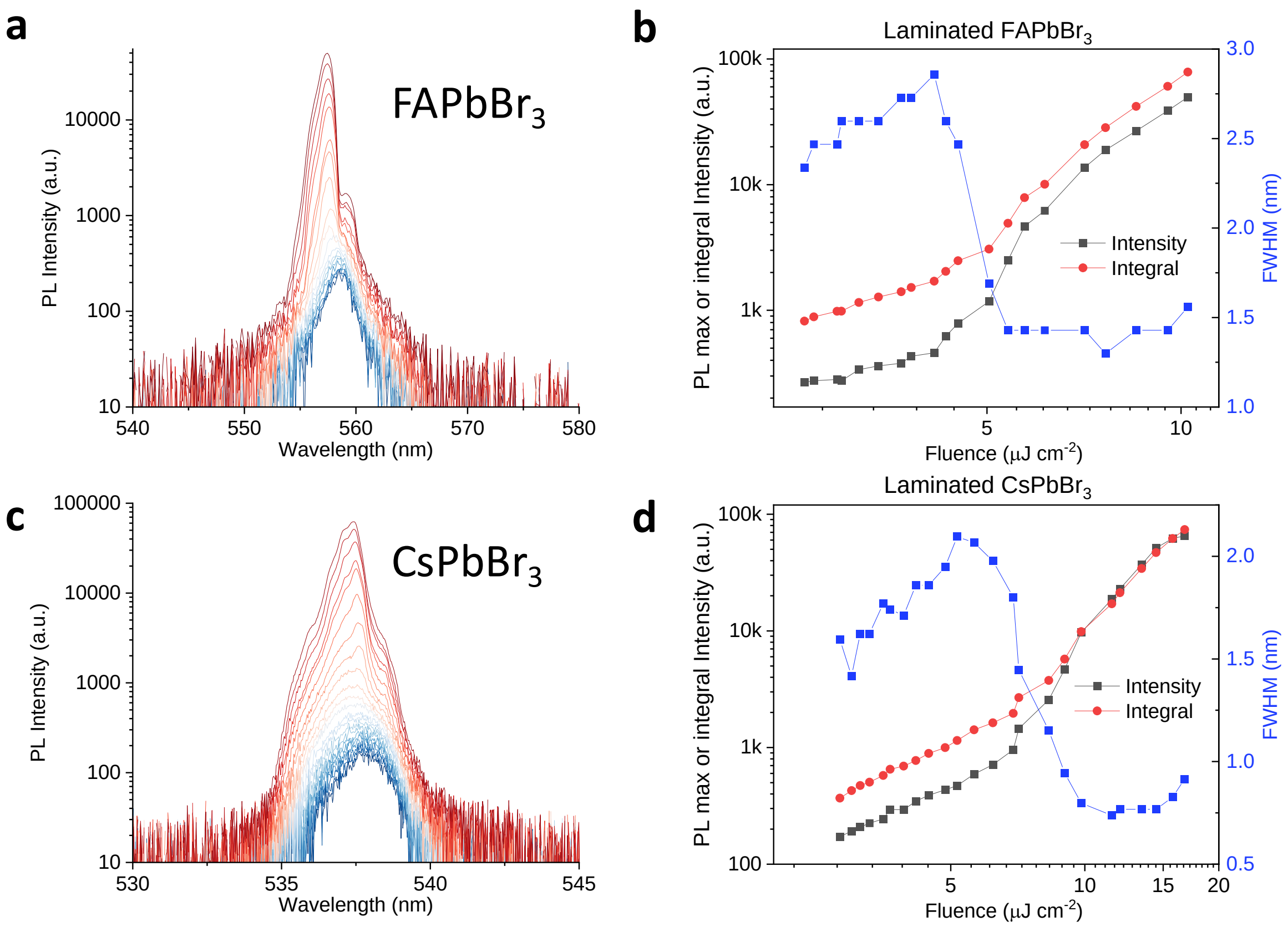

Supplementary Figure 15. Comparison of laser thresholds of laminated VCSELs. a, c, Fluence dependent PL spectra obtained from $FAPbBr_3$ (a)**,** and $CsPbBr_3$ (c). **b, d,** Evolution of PL intensity, integral PL intensity, and FWHM of $FAPbBr_3$ (b)**,** and $CsPbBr_3$ (d) VCSELs across different excitation fluences.

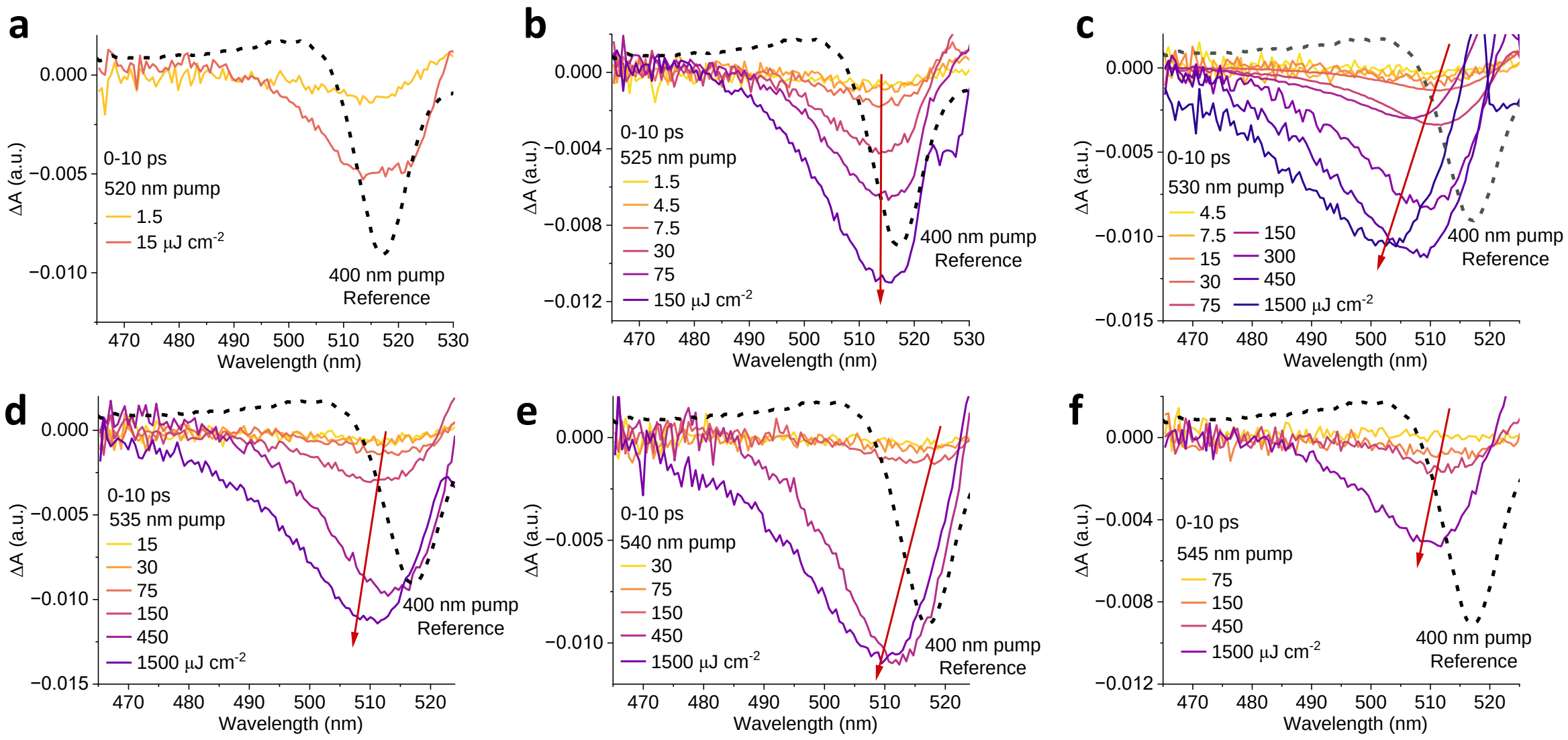

Supplementary Figure 16. TA spectra of single-layer $CsPbBr_3$ as a function of pump fluence and wavelength. a-f, TA spectra (0–10 ps) at pump fluences ranging from 1.5μJ cm^{-2} to 1500 μJ cm^{-2} and pump wavelengths from near-resonant (520 nm) to below-bandgap (525–545 nm).

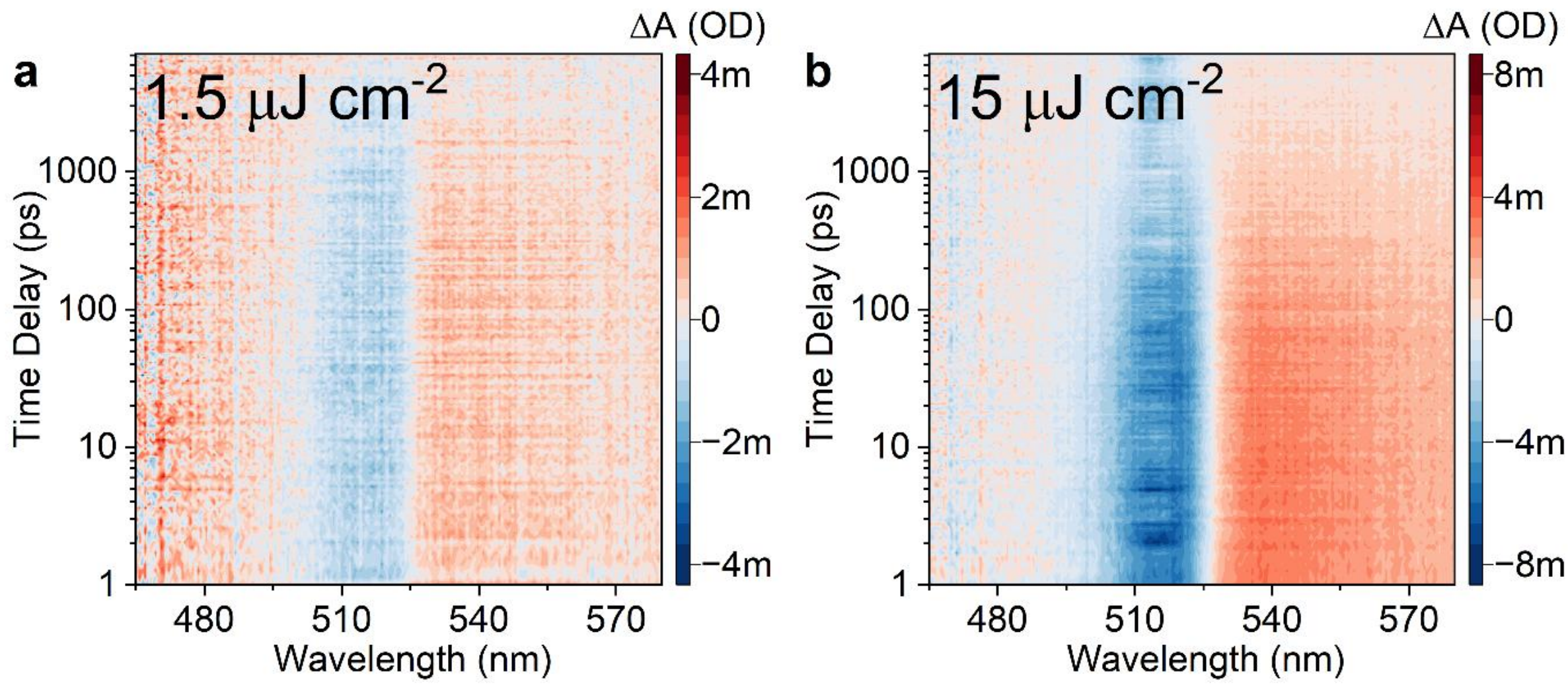

Supplementary Figure 17. Fluence dependent transient absorption (TA) spectra of single layer $CsPbBr_3$ films. a-i, TA maps obtained from single layer $CsPbBr_3$ films. The pump fluence was increased from low value 1.5 to ultrahigh value 15 μJ cm^{-2}. A 520 nm pulsed laser was used here to excite the samples.

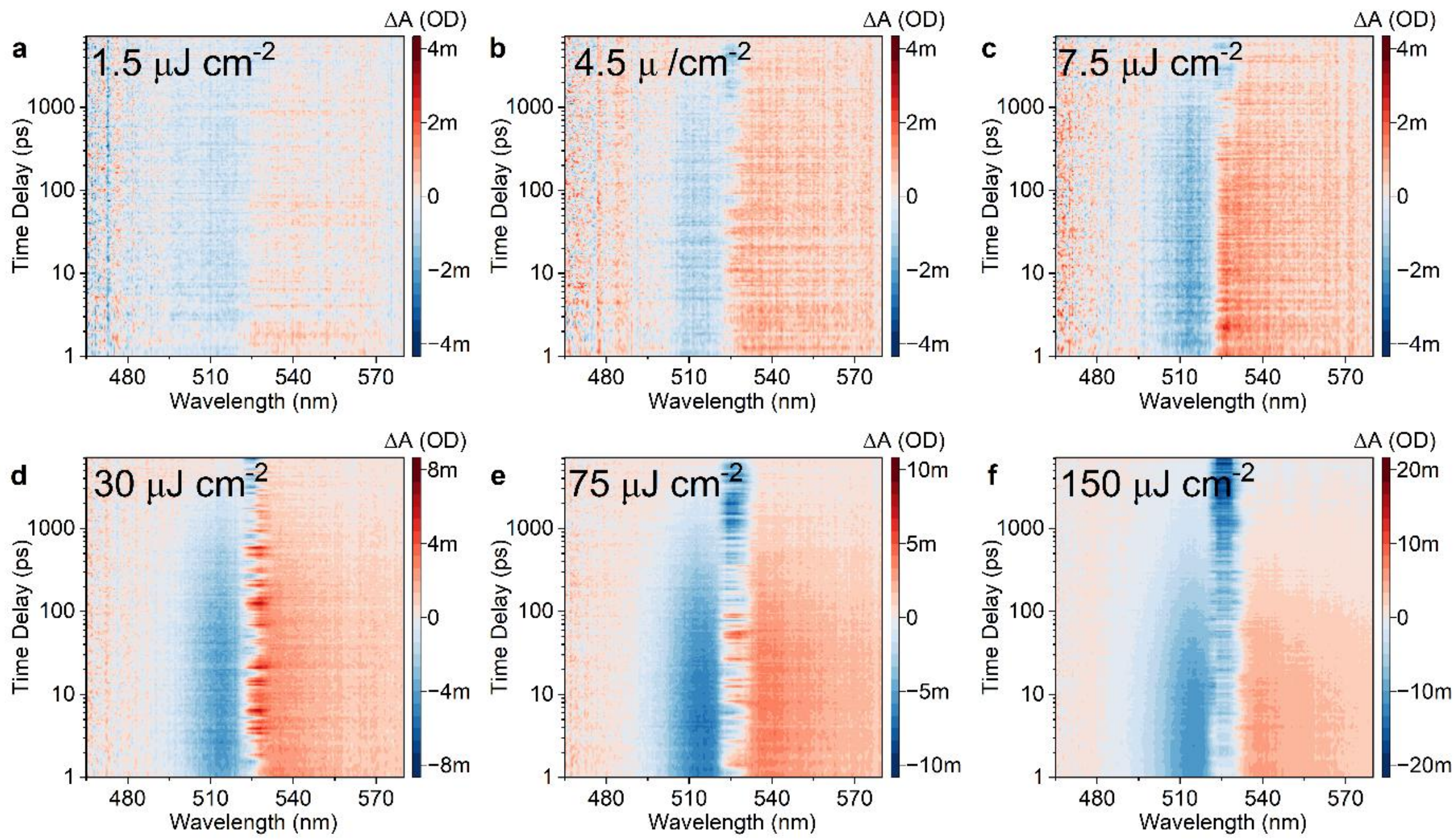

Supplementary Figure 18. Fluence dependent transient absorption (TA) spectra of single layer $CsPbBr_3$ films. a-i, TA maps obtained from single layer $CsPbBr_3$ films. The pump fluence was increased from low value 1.5 to ultrahigh value 150 μJ cm^{-2}. A 525 nm pulsed laser was used here to excite the samples.

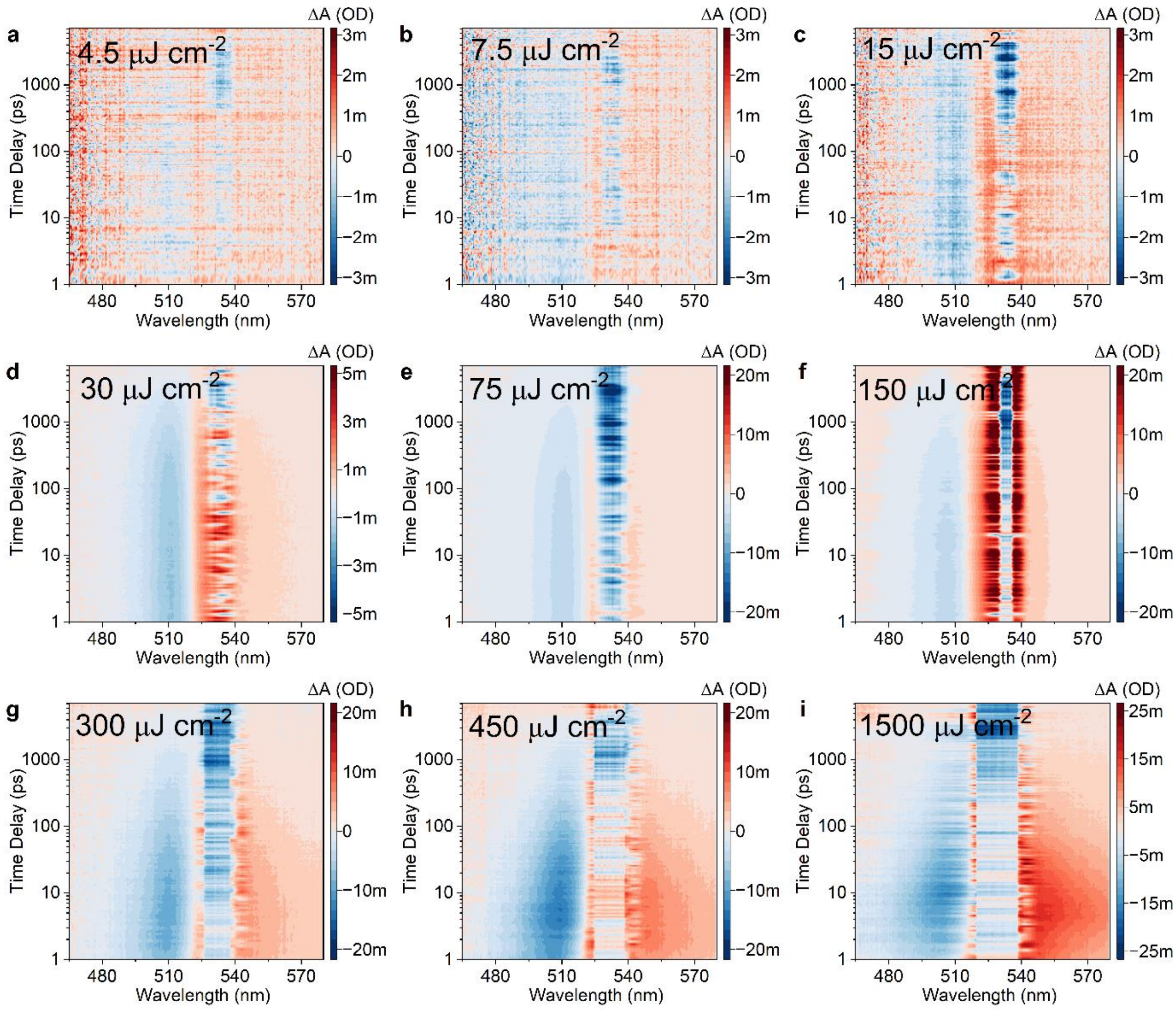

Supplementary Figure 19. Fluence dependent transient absorption (TA) spectra of single layer $CsPbBr_3$ films. a-i, TA maps obtained from single layer $CsPbBr_3$ films. The pump fluence was increased from low value 4.5 to ultrahigh value 1500 μJ cm^{-2}. A 530 nm pulsed laser was used here to excite the samples.

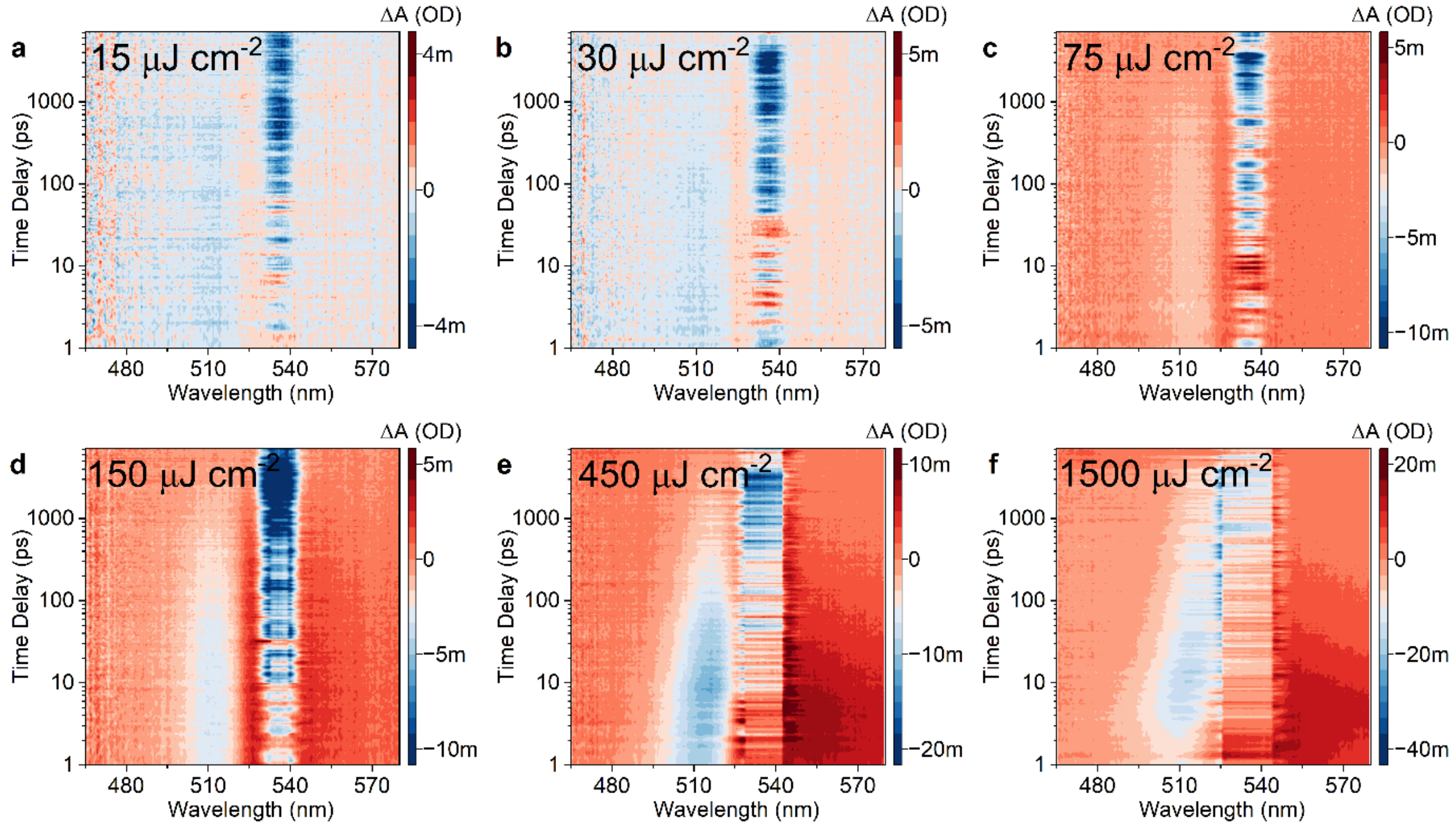

Supplementary Figure 20. Fluence dependent transient absorption (TA) spectra of single layer $CsPbBr_3$ films. a-i, TA maps obtained from single layer $CsPbBr_3$ films. The pump fluence was increased from low value 15 to ultrahigh value 1500 μJ cm^{-2}. A 535 nm pulsed laser was used here to excite the samples.

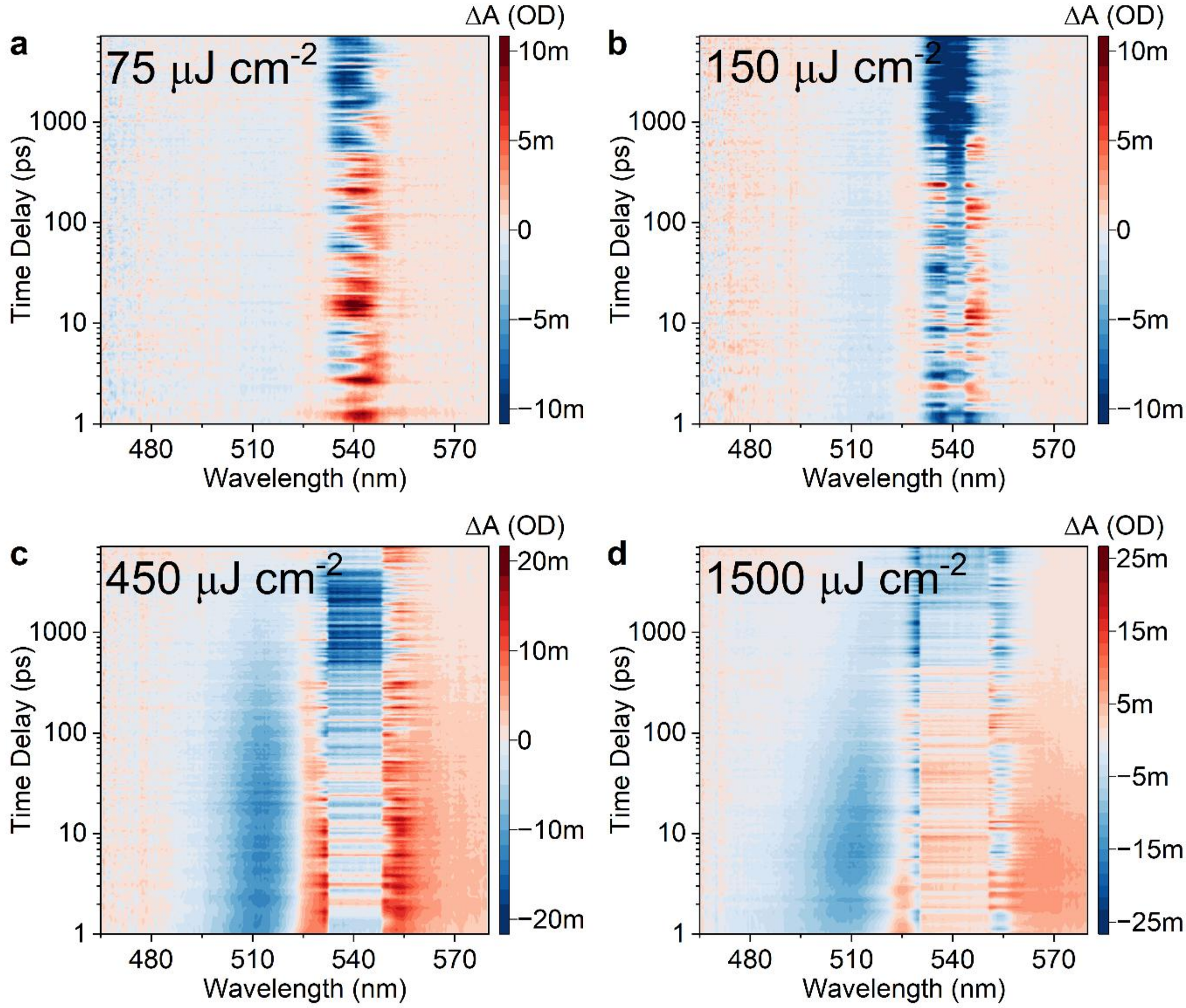

Supplementary Figure 21. Fluence dependent transient absorption (TA) spectra of single layer $CsPbBr_3$ films. a-i, TA maps obtained from single layer $CsPbBr_3$ films. The pump fluence was increased from low value 75 to ultrahigh value 1500 μJ cm^{-2}. A 540 nm pulsed laser was used here to excite the samples.

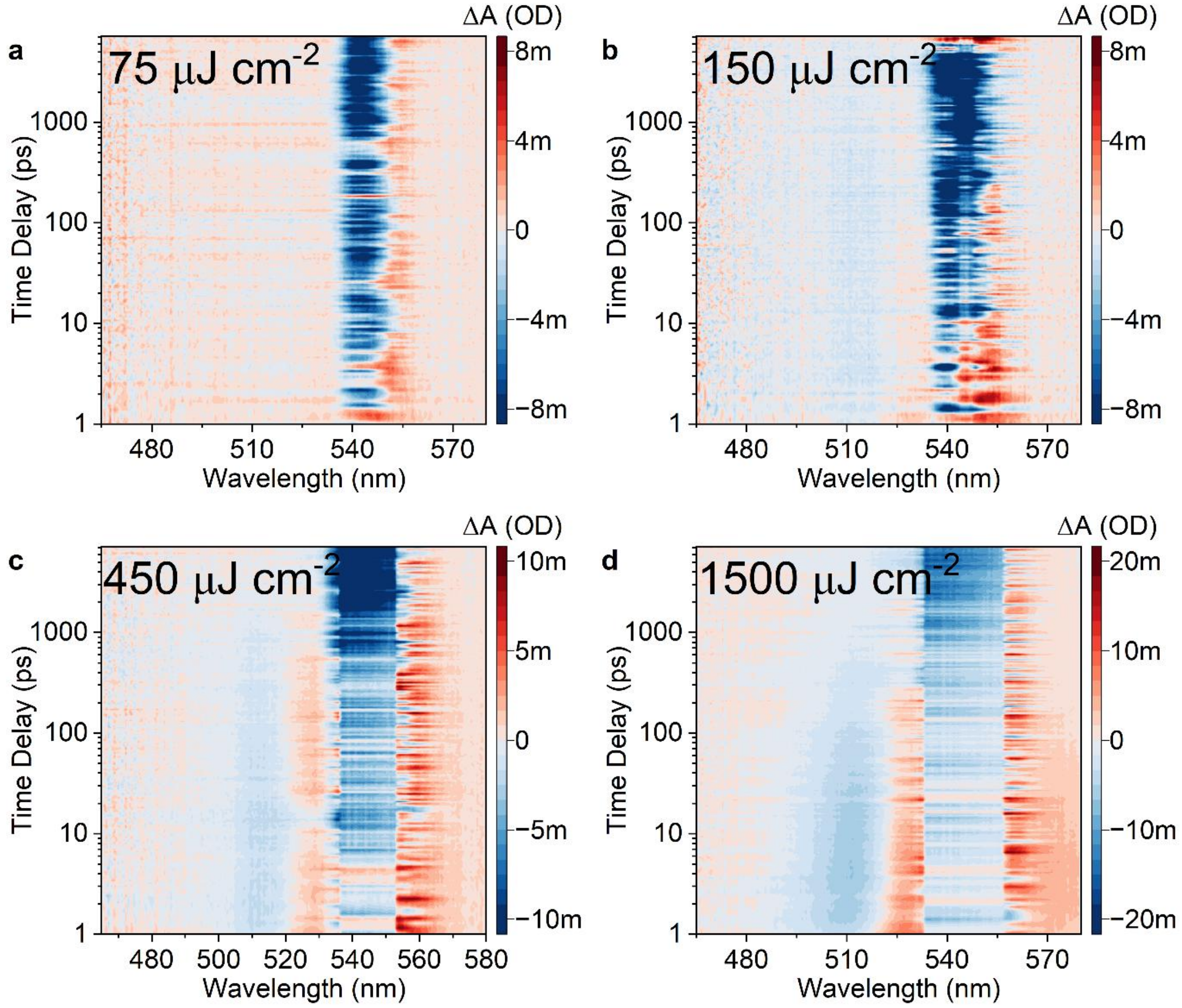

Supplementary Figure 22. Fluence dependent transient absorption (TA) spectra of single layer $CsPbBr_3$ films. a-i, TA maps obtained from single layer $CsPbBr_3$ films. The pump fluence was increased from low value 75 to ultrahigh value 1500 μJ cm^{-2}. A 545 nm pulsed laser was used here to excite the samples.

Supplementary References

1. Milanese, S., De Giorgi, M.L., Anni, M., Bodnarchuk, M.I., Cerdán, L., Rationalizing the amplified spontaneous emission mechanism in $CsPbBr_3$ perovskite nanocrystals films by means of optical gain measurements. *Adv. Optical Mater.* **12**, 2401078 (2024).
2. Cerdán, L. Simultaneous retrieval of optical gains, losses, and threshold in active waveguides. *Optics & Laser Technology* **121**, 105814 (2020).
