# Supplementary material for: Ultrafast directional hot carrier transfer in bilayer perovskite heterojunctions
Source: Nat Commun. 2026 Sep 7;17:9555. doi: 10.1038/s41467-026-76790-z (PMC13550544; doi:10.1038/s41467-026-76790-z)
Supplement: Supplementary file 2 [file 41467_2026_76790_MOESM2_ESM.pdf]

nature portfolio

Peer Review File

Ultrafast directional hot carrier transfer in bilayer perovskite heterojunctions

Corresponding Author: Professor Ulrich Paetzold

This file contains all reviewer reports in order by version, followed by all author rebuttals in order by version.

Version 0:

Reviewer comments:

Reviewer #1

(Remarks to the Author)
I co-reviewed this manuscript with one of the reviewers who provided the listed reports. This is part of the Nature Communications initiative to facilitate training in peer review and to provide appropriate recognition for Early Career Researchers who co-review manuscripts.

Reviewer #2

(Remarks to the Author)
I read the manuscript “Ultrafast directional hot carrier transfer in 1 bilayer perovskite heterojunctions” and I find difficult to catch its core message.
Given that the authors demonstrate the transfer of hot carriers from two different phases of perovskites, since it happens in a few ps timescale, as “more classical” energy transfer between two thin phases (or blended at the nanoscale) of different bandgap, why should it be a more effective solution in lowering the lasing trashold?

Then, what I find not well explained is why the bilayer results in a low but also wavelength independent laser threshold. The laser threshold is proportional to the ratio between the time carriers injected in the electronic state take to relax to the bottom(top) of the conduction(valence) band, which includes the time carrier will take to transfer from one layer to the other and the time carriers take to cool and the time such state takes to get depleted. If we assume that the depletion time does not change I still expect an excitation energy dependence.

Then, how the authors can exclude the role of defects at the interfaces, e.g. the formation of a sort of three states system, which would also help the population inversion, since the heterojunction is not “clean” as one would find in epitaxially grown systems?

To conclude I do not find the manuscript convincing in its current form.

Reviewer #3

(Remarks to the Author)
This paper investigates the ultrafast directional transport mechanism of hot carriers in perovskite heterojunctions. By designing a $CsPbBr_3/FAPbBr_3$ bilayer bulk heterojunction and combining ultrafast spectroscopy with device fabrication, the study demonstrates a cooling-rate-gradient-driven directional transfer of hot carriers, which enables the successful realization of a low-threshold perovskite vertical-cavity surface-emitting laser (VCSEL). The research is positioned at the forefront of optoelectronic devices, featuring an innovative experimental design and robust data support. Its conclusions provide valuable guidance for the development of perovskite lasers, photodetectors, and other related devices. Overall, the paper exhibits a clear logical structure, well-organized figures and tables, and fluent writing. However, further refinement/major reversion is needed in the mechanistic interpretation, experimental details, data comparison, and stability analysis, and some conclusions require additional validation.
1. Several issues regarding Figure 2 require attention. First, the scale bars are missing in Figures 2d, 2e, 2g, and 2h, which are essential for interpreting the morphological features. Second, the substrate used in Figures 2d and 2e is ITO, whereas

the $CsPbBr_3$/$FAPbBr_3$ bilayer in Figure 2f is deposited on glass. Given that ITO and glass exhibit different surface chemistries and physical properties—factors that can significantly influence the crystallization process of perovskites—this inconsistency in substrate selection necessitates clarification. Additionally, the morphologies of $CsPbBr_3$ shown before (Figures 2g) and after (Figure 2i) the lamination process appear markedly different. The underlying mechanism driving this morphological evolution should be discussed to validate the integrity of the lamination procedure. The horizontal axis label in Figure 3h, "Pump power (μJ/cm²)," is a misnomer; the correct term should be "Pump fluence."
2. The energy barrier and kinetic details of the hot carrier transfer process require further clarification. Although the paper demonstrates that hot carrier transfer from $FAPbBr_3$ to $CsPbBr_3$ is driven by differences in cooling rates, it does not specify the energy barriers at the heterojunction interface (e.g., the transfer barriers for electrons and holes), nor does it provide a quantitative analysis of the transfer time constants. For instance, the transfer is only described as occurring on a "picosecond" timescale, without precise values. To address this, it is recommended to extract the rate constants of hot carrier transfer by fitting transient absorption (TA) kinetic data, and to supplement the energy band diagram with a quantitative determination of the barrier heights. Such additions would significantly enhance the understanding of the transfer kinetics.
3. Defects play a critical role in determining the TA kinetics, optical gain, and laser performance of halide perovskite semiconductors. The nature of defects—whether shallow or deep—governs the relaxation pathways and rates of hot carriers. Specifically, hot carriers can be directly captured by trap states without first relaxing to the band edge (Nat. Commun. 11, 2712 (2020)). More critically, deep-level defects accelerate hot-carrier cooling by providing efficient energy dissipation channels, thereby weakening both the hot-phonon bottleneck and Auger reheating effects that otherwise slow energy loss (Nat. Commun. 15, 8120 (2024); Adv. Funct. Mater. 2025, e17023).
Optical gain requires population inversion, which depends on maximizing the number of excited carriers at the conduction band edge for stimulated emission. However, defect states—particularly deep-level traps—act as non-radiative recombination centers, diverting carriers from radiative processes and dissipating energy as heat. This parasitic pathway depletes the carrier population available for optical amplification, reducing the optical gain coefficient (Nat. Commun. 15, 5484 (2024)). Consequently, a higher excitation density is required to achieve population inversion, leading to an increased laser threshold. Furthermore, trap states facilitate Auger recombination—a process that would otherwise require three-particle interactions—thereby shortening the gain lifetime under high-injection conditions. These defects also introduce energy barriers that increase optical waveguide losses (reducing the cavity quality factor), both of which impede efficient photon amplification (Nat. Commun. 15, 5484 (2024); Adv. Mater. 35, 2306102 (2023)).
In this work, the lamination process (120 °C, 100 MPa for 5 min) can markedly alter the defect density (Nat. Commun. 11, 5402 (2020)). Given that the fabricated hot-carrier laser devices achieve a threshold as low as 1.4 μJ/cm² under nanosecond pulsed excitation—five times lower than that of individual perovskite layers—a careful discussion of defect in the laminated samples is essential. Such analysis would ensure that the observed hot-carrier dynamics and performance enhancement is primarily attributed to directional hot-carrier transport rather than defect-mediated effects.
4. Given that steady-state absorption, transient absorption, and laser performance measurements are critically dependent on the thickness of the perovskite photoactive layer, because film thickness significantly affects carrier transport and optical confinement effects. a detailed specification of the sample thickness is essential. Such information is crucial for ensuring the validity and comparability of the experimental results.
5. The proposition that hot carrier transfer constructs an ideal three-level system in the heterojunction requires rigorous quantitative support. While this concept is central to the work, the current manuscript lacks essential quantitative data to substantiate it. Specifically, it does not delineate the energy level spacing of the proposed three-level system, nor does it determine the critical carrier density necessary for achieving population inversion. Furthermore, the optical gain coefficient—a key parameter linking the three-level system to laser performance—has not been extracted from the experimental data. To address this, it is recommended that the authors provide a detailed energy level diagram with labeled energy values and calculate the optical gain using the PL or ASE or TA results. Such an analysis would quantitatively establish the contribution of the three-level system to the observed reduction in laser threshold.
6. The comparison of laser thresholds with existing perovskite devices is insufficiently comprehensive. While the paper reports that the fabricated VCSEL exhibits a threshold of 1.4 μJ/cm²—five times lower than that of a single perovskite layer—this benchmark is limited to an internal reference. A broader contextualization is needed against state-of-the-art values reported for other perovskite VCSELs and planar heterojunction lasers. Moreover, the work would benefit from a critical analysis of its advantages and limitations relative to other material systems, such as colloidal nanocrystal and quasi-2D perovskite lasers. Therefore, it is recommended that the authors include an up-to-date comparison table of threshold values from representative perovskite laser devices. This addition would effectively highlight the progress and innovation of the present work within the field.
7. Light is fundamentally a wave characterized by both amplitude and phase. Laser light, by definition, is coherent—meaning it exhibits well-defined phase relationships in both time and space (Svelto, O. Principles of Lasers 475-504 (Springer US, 2010).; Nat. Photonics 3, 546–549 (2009).). To conclusively demonstrate that a sharp emission peak originates from lasing rather than amplified spontaneous emission (ASE) or other incoherent processes, evidence beyond intensity-dependent measurements is required. While the current work shows how the peak amplitude evolves with pump fluence—a necessary condition for identifying a lasing threshold—it does not constitute sufficient proof. The critical missing piece is phase information. Therefore, it is recommended that the authors provide direct evidence of coherence, such as measurements of temporal coherence (e.g., via interferometry to determine coherence length) or spatial coherence (e.g., via Young's double-slit experiment). Such data would unambiguously confirm the coherent nature of the emission and solidify the claim of achieving lasing.

Reviewer #4

(Remarks to the Author)

This manuscript reports laminated CsPbBr3/FAPbBr3 bilayer heterojunctions and proposes that a dynamic population gradient, arising from different hot-carrier cooling pathways, drives ultrafast directional hot-carrier transfer from FAPbBr3 to CsPbBr3. The authors further claim that this process increases the carrier population in CsPbBr3 and thereby reduces the ASE and lasing thresholds. The idea is interesting and potentially important. In particular, using interfacial hot-carrier dynamics to improve optical gain is an appealing concept.
However, in my view, the current experimental evidence is still insufficient to support the central mechanistic claims of the paper. Several key observations remain open to alternative interpretations. As a result, I do not believe the manuscript, in its present form, meets the standard for publication in Nature Communications. Below are several issues
1. The authors argue that hot-carrier transfer from FAPbBr3 to CsPbBr3 increases the carrier density in CsPbBr3 and promotes population inversion. However, for lasing or ASE, the relevant population is the band-edge carrier population rather than the transient hot-carrier population. Under the type-II alignment proposed in the manuscript, band-edge electrons in CsPbBr3 should energetically favor transfer to FAPbBr3, which would deplete, rather than enhance, the steady-state band-edge population in CsPbBr3. The manuscript does not sufficiently explain how the proposed hot-carrier injection competes with the energetically favorable band-edge charge separation. A quantitative discussion of these competing channels is essential. Without such an analysis, the link between ultrafast hot-carrier transfer and lower lasing threshold remains speculative.
2. The manuscript assumes that under 530 nm resonant excitation, only FAPbBr3 is initially excited, and at low fluence no CsPbBr3 signal is observed, which the authors regard as consistent with the type-II alignment. However, if the interface indeed supports efficient interfacial transfer, then the hole-transfer channel from FAPbBr3 to CsPbBr3 should also be considered explicitly. In that case, one may also expect signatures associated with CsPbBr3 even under lower-power excitation. The current discussion focuses selectively on the proposed hot-electron pathway but does not rigorously analyze why other interfacial processes expected for a type-II junction are absent or negligible.
3. Because the bandgaps of FAPbBr3 and CsPbBr3 are quite close, excitation nominally resonant with one component may still partially excite the other, especially under high fluence. In particular, under strong pumping, contributions from spectral tail absorption, two-photon absorption, phonon-assisted upconversion, or other nonlinear effects cannot be ruled out. This issue is especially important for the interpretation of the 530 nm resonant-pump TA data and the assignment of the delayed Cs-related signal to hot-carrier transfer induced by Auger heating. At present, the manuscript does not provide sufficient control experiments to exclude these competing possibilities. Fluence-dependent controls on the individual single-component films, together with excitation-wavelength-dependent measurements and nonlinear absorption controls, are needed.
4. The manuscript attributes the lower ASE and lasing thresholds primarily to hot-carrier transfer. However, ASE and lasing thresholds are also highly sensitive to film morphology, roughness, cavity quality, thickness uniformity, interface flatness, and optical confinement. In fact, the authors themselves show that lamination dramatically changes the film morphology and makes the bilayer much flatter and denser. Therefore, it is difficult to separate the effect of carrier-transfer physics from improvements in cavity or scattering loss. This concern is particularly serious because the threshold differences between the pristine films and heterostructures are not supported by any statistical analysis across multiple devices or batches. The manuscript should provide device-to-device statistics, error bars, and sample numbers before drawing a strong conclusion that the reduced threshold originates predominantly from hot-carrier transfer.
5. The cross-sectional SEM images show that the pristine CsPbBr3 and FAPbBr3 films are presented on ITO substrates, whereas the laminated bilayer is shown on glass. At minimum, the manuscript should explain clearly why the substrate changes between these panels. In addition, comparing Fig. 2e and 2f, the morphology of the FAPbBr3 layer undergoes a dramatic transformation, becoming extremely dense and flat after lamination. What causes such a profound morphological change through a simple solid-state pressing process?
6. In Fig. 3g, the ASE from the bilayer is dominated by a peak near 540 nm, which also appears closer to emission from the FA component. The manuscript does not discuss this sufficiently. This seems difficult to reconcile with the core claim that hot carriers are injected into the CsPbBr3 layer and that the gain enhancement is mainly realized there. The possibility of reabsorption, cavity filtering, or mixed contributions from both layers should be carefully evaluated.
7. In Fig. 3c, the GSB of the Cs layer in the heterojunction decays to a very low level after 20 ps. However, in Fig. 3e, the GSB of the Cs layer in the two control samples remains highly significant after 20 ps. What accounts for this substantial difference in decay behavior? Are these data from different batches of samples, or are there differences in measurement conditions or probe wavelengths? Similarly, when comparing Fig. 3c and 3f, clear differences in the decay behavior of the FA layer are also observed.

Version 1:

Reviewer comments:

Reviewer #1

(Remarks to the Author)
I co-reviewed this manuscript with one of the reviewers who provided the listed reports. This is part of the Nature Communications initiative to facilitate training in peer review and to provide appropriate recognition for Early Career Researchers who co-review manuscripts.

Reviewer #3

(Remarks to the Author)
The author has completely answered my questions, and the related experiments and revisions have improved the quality of

the manuscript. Therefore, it is recommended to publish it in Nature Communications.

Reviewer #4

(Remarks to the Author)
The authors have addressed all of my concerns, and I have no additional comments.

Open Access This Peer Review File is licensed under a Creative Commons Attribution 4.0 International License, which permits use, sharing, adaptation, distribution and reproduction in any medium or format, as long as you give appropriate credit to the original author(s) and the source, provide a link to the Creative Commons license, and indicate if changes were made.
In cases where reviewers are anonymous, credit should be given to 'Anonymous Referee' and the source.
The images or other third party material in this Peer Review File are included in the article's Creative Commons license, unless indicated otherwise in a credit line to the material. If material is not included in the article's Creative Commons license and your intended use is not permitted by statutory regulation or exceeds the permitted use, you will need to obtain permission directly from the copyright holder.
To view a copy of this license, visit https://creativecommons.org/licenses/by/4.0/

Manuscript ID: NCOMMS-26-013613-T

Title: "Ultrafast directional hot carrier transfer in bilayer perovskite heterojunctions"

Author(s): Shangpu Liu, Changhao Gao, Mohammad Gholipoor, Tonghan Zhao, Andrii Shcherbakov, Zhiwei Lei, Pirmin Tischler, Yiqin Xie, Nils Rosemann, Uli Lemmer, Yang Li, Felix Deschler, Ulrich Paetzold

We sincerely thank the reviewers for their insightful and constructive comments, which highlighted key areas where the manuscript required further clarification, quantitative analysis, and experimental validation. Below, we provide a detailed point-by-point response to address all comments. To strengthen our mechanistic claims, we have performed additional experiments, analyses, and theoretical calculations that supply rigorous evidence for the proposed hot-carrier transfer mechanism and direct confirmation of lasing.

REVIEWER COMMENTS

Reviewer #1 (Remarks to the Author):

I co-reviewed this manuscript with one of the reviewers who provided the listed reports. This is part of the Nature Communications initiative to facilitate training in peer review and to provide appropriate recognition for Early Career Researchers who co-review manuscripts.

Reviewer #2 (Remarks to the Author):

I read the manuscript “Ultrafast directional hot carrier transfer in 1 bilayer perovskite heterojunctions” and I find difficult to catch its core message.

Given that the authors demonstrate the transfer of hot carriers from two different phases of perovskites, since it happens in a few ps timescale, as “more classical” energy transfer between two thin phases (or blended at the nanoscale) of different

bandgap, why should it be a more effective solution in lowering the lasing trashold?

Our Response: We thank the reviewer for this important question. Here, we clarify that the key distinction between our hot carrier transfer mechanism and classical energy transfer lies in the drive force and directionality.

In a conventional Type-II heterojunction with band alignment as measured here ($CsPbBr_3$ CBM higher than $FAPbBr_3$ by 0.09 eV), thermalized carriers at the band edges flow from the high-bandgap material ($CsPbBr_3$) to the low-bandgap material ($FAPbBr_3$). This depletes carriers from the very material we wish to lase from. Our control experiments confirm this: under low-fluence excitation at 530 nm (resonant with $FAPbBr_3$ only), we observe no $CsPbBr_3$ ground-state bleach – meaning no carriers reach $CsPbBr_3$ via classical transfer. This, and the higher lasing thresholds in $FAPbBr_3$ (Fig. 3h) hinder classical energy transfer (Type-II) and lasing in $CsPbBr_3$, and cannot explain our observed, unexpected, results (decreased lasing threshold in $CsPbBr_3$).

In contrast, our hot carrier transfer mechanism occurs from low-bandgap to high-bandgap material, and equivalent to a (beneficial) transfer from high lasing threshold to low lasing threshold material. Hot carriers generated in $FAPbBr_3$ (lower bandgap) are transferred into $CsPbBr_3$ (higher bandgap) before they cool to the band edge. This is possible only because the transferred carriers have excess kinetic energy (hot carriers) that allows them to surmount the 0.09 eV conduction band offset. The driving force is not band-edge alignment but a cooling-rate gradient – $FAPbBr_3$ exhibits a strong hot-phonon bottleneck (slow cooling), while $CsPbBr_3$ cools rapidly, creating a population imbalance that drives hot carriers across the interface.

In our current picture, this behavior constructs an ideal three-level laser system, which lowers the lasing thresholds

Pump: Excitation creates hot carriers in $FAPbBr_3$ (level 3).

Fast transfer and cooling: Hot carriers are funnelled into hot state of $CsPbBr_3$ and

cooling down to its excited state band edge within a few ps (level 2).
Lasing transition: Carriers relax from excited state band edge to the $CsPbBr_3$ ground state (level 1), emitting light.

This three-level scheme achieves population inversion at a much lower threshold than a conventional two-level system, because the pump does not have to directly create a high density of carriers in the upper lasing state – instead, carriers are funneled to these from a separate reservoir (the hot carriers in FAPbBr3 in our case). Our data in Fig. 3h and 3i support this: laminated $FAPbBr_3$ shows an increased threshold compared to pure $FAPbBr_3$, conforming carriers are transferred to $CsPbBr_3$ side; the threshold reduction of laminated $CsPbBr_3$ relative to pure $CsPbBr_3$ is ~4× at 355 nm (where hot carrier effects are strong) but only ~2× at 460 nm (where hot carriers are fewer), confirming that hot carriers are essential for the low threshold. This principle – using hot carrier injection to achieve inversion without directly pumping the upper lasing state to high density – is a well-established strategy in classical laser physics, especially for III-V semiconductor lasers.

Further both carrier cooling and hot-carrier injection into $CsPbBr_3$ are extremely fast (the transfer time constant is ~9.8 ps, while initial cooling occurs in <0.2 ps ($CsPbBr_3$)). This enables efficient carrier accumulation in the $CsPbBr_3$ phase before any significant decay can take place. The TA decay kinetics further show that the carrier lifetime in $CsPbBr_3$ is only tens of ps (Table S2), which is much shorter than the hundreds of picoseconds observed in $FAPbBr_3$. Moreover, most conventional carrier transport across the bilayer heterostructure is reported to occur on timescales longer than 100 ps (*JACS*, 140(46), 15675-15683 (2018).; *ACS Photonics*, 9(6), 2008-2014, (2022).; *Angew. Chem. Int. Ed.*, 64, e202506436 (2025).). Thus, any carriers that reach the conduction band edge of $CsPbBr_3$ may recombine radiatively to contribute to optical gain before they can transfer back to $FAPbBr_3$.

Thus, the core message is not only simply “picosecond transfer”, but rather: We exploit intrinsic differences in hot carrier cooling rates to drive directional transfer

from a low-bandgap to a high-bandgap perovskite, creating selective population of the levels in a three-level system that achieves population inversion at much lower pump density than classical energy transfer or single-layer lasing – a direct experimental proof of the advantage. We have revised the manuscript to state this more explicitly in the Results sections, and we thank the reviewer for prompting this clarification.

We ***added*** on the Page 11 of revised manuscript, that "Based on the hot carrier transfer mechanism discussed above, the planar $FAPbBr_3/CsPbBr_3$ heterojunctions are expected to mimic an ideal three-level system~~,~~. High-energy photoexcitation generates hot carriers in $FAPbBr_3$ (level 3), which exhibits a relatively slow cooling rate. These hot carriers are then rapidly funneled into the $CsPbBr_3$ side within a few picoseconds, and subsequently cool to the band-edge excited state of $CsPbBr_3$ (level 2) on a sub-picosecond timescale. Finally, the carriers radiatively relax to the $CsPbBr_3$ ground state (level 1), emitting light (Fig. 1b). Such a three-level configuration would lead~~ing~~ to an enhancement of optical gain in $CsPbBr_3$."

We have ***modified*** our schematic illustration in Fig. 1 to better explain the difference between thermal-imbalance-driven hot carrier transfer and conventional energy gradient-driven carrier transfer mechanism.

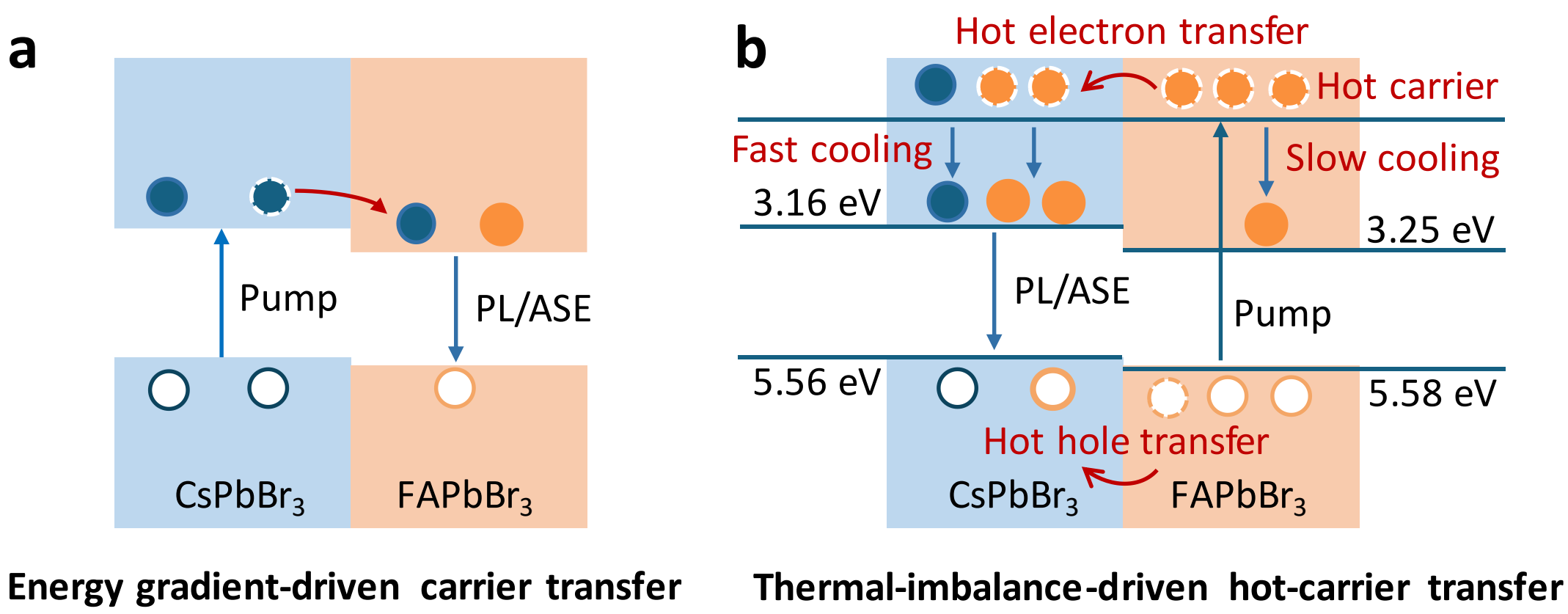

Figure 1. Carrier transfer mechanisms in semiconductor heterojunctions. a, Schematic illustration of conventional carrier transfer between high and low band

gap semiconductors. Red arrows highlight the flow of cooled carriers across the interface driven by band energy gradient. **b, c** Hot carrier transfer from a low band gap ($FAPbBr_3$) semiconductor to a high band gap semiconductor ($CsPbBr_3$). Under high-energy pumping conditions (b), hot carriers are generated in both high and low band gap semiconductors, while different hot carrier cooling rates influence the transfer dynamics, directing hot carriers into materials with faster cooling pathways. With resonant pumping at low band gap semiconductor (c), hot carriers with excess energy above the band minimum (generated by scattering, Auger recombination or phase space filling) can move across the interface to the high band gap semiconductor.

We ***edited*** on the Page 4 of revised manuscript, that "We hypothesize two types of mechanisms for the hot carrier transfer mechanisms in semiconductor heterojunctions. In the first scenario (Scenario I), With excess-energy pumping, both low and high-bandgap semiconductors are excited, resulting in hot carrier generation on both sides (Fig. 1b). Directed hot carrier transfer is then driven by differences in hot carrier cooling rates (thermal imbalance), which are intrinsically related to the materials' properties. Rapid hot carrier cooling reduces the hot carrier population, whereas slow cooling—such as the hot phonon bottleneck effect (*1*, *11*, *14*)—leads to accumulation of hot carriers within the material. This population imbalance can drive hot carrier transfer from the slow-cooling material to the fast-cooling one, and then hot carriers further cool down at the fast-cooling materials side, thereby aiding enhancing their carrier population inversion at the CB minimum. Notably, the hot carrier transfer can induce substantial population inversion in the excited states, which has been widely reported in III-V semiconductor laser (*24*-*26*). Thus, when combined with bandgap transitions, this hot carrier transfer mechanism effectively creates a three-level system that enhances optical gain and lowers lasing thresholds in heterostructures of slow- and fast-cooling materials. This innovative approach leverages hot carrier dynamics to optimize carrier population distributions and emission processes, offering a promising pathway for the development of hot-carrier-based optical devices and lasing systems with improved efficiencies."

25. Tolstikhin, V. I., & Willander, M. Resonant-tunneling injection hot electron laser: An approach to picosecond gain-switching and pulse generation. Applied Physics Letters, 67(18), 2684-2686 (1995).

26. Hoskens, R. C. P., Theo G. van de Roer, E. Smalbrugge, J. J. M. Kwaspen, V. I. Tolstikhin, Hark Hoe Tan, C. Jagadish, and Gerard A. Acket. "Hot electron injection laser controlled carrier-heating induced gain switching." In *Conference on Optoelectronic and Microelectronic Materials and Devices, 2004.*, 397-400. IEEE, 2004.

We have now ***performed*** a quantitative kinetic analysis of our TA data to extract the hot-carrier transfer kinetics to confirm that hot-carrier transfer is the primary source of population in the $CsPbBr_3$. The details are provided below and in the updated Supplementary Information.

We have ***added*** on Method part of revised manuscript, that "Room-temperature TA measurements were performed by commercial TA setup (Ultrafast Systems, HELIOS). We globally fitted the decay kinetic traces of $CsPbBr_3$ and $FAPbBr_3$ (data in Fig. 3c) to extract hot carrier transfer components ($CsPbBr_3$: hot carrier gain/bleach, $FAPbBr_3$: hot carrier transfer out). We use a comprehensive kinetic model that includes hot-carrier cooling, hot carrier transfer ($\tau_{transfer}$), and biexponential decay components (τ_{fast}, τ_{slow}) for both phases with corresponding branching ratios. The model also accounts for an instrument response function (IRF, deconvolved during fitting). All parameters were shared globally across both traces, and the fit was performed by least-squares minimization with regularization on the fast components. The fitted hot carrier transfer constant and carrier decay constant were listed in Table S2."

Table S2. Fitting parameters of the hot carrier transfer in $CsPbBr_3/FAPbBr_3$ laminated heterojunctions.

Phases	$\tau_{transfer}$	τ_{fast}	τ_{slow}

Laminated $FAPbBr_3$	**1.2 ± 0.7 ps**	258 ± 1 ps (Amplitude 0.14)	~984 ± 1 ps (Amplitude 0.86)
Laminated $CsPbBr_3$	**9.8 ± 0.6 ps**	6.3 ± 0.6 ps (Amplitude 0.94)	~4890 ± 5 ps (Amplitude 0.06)

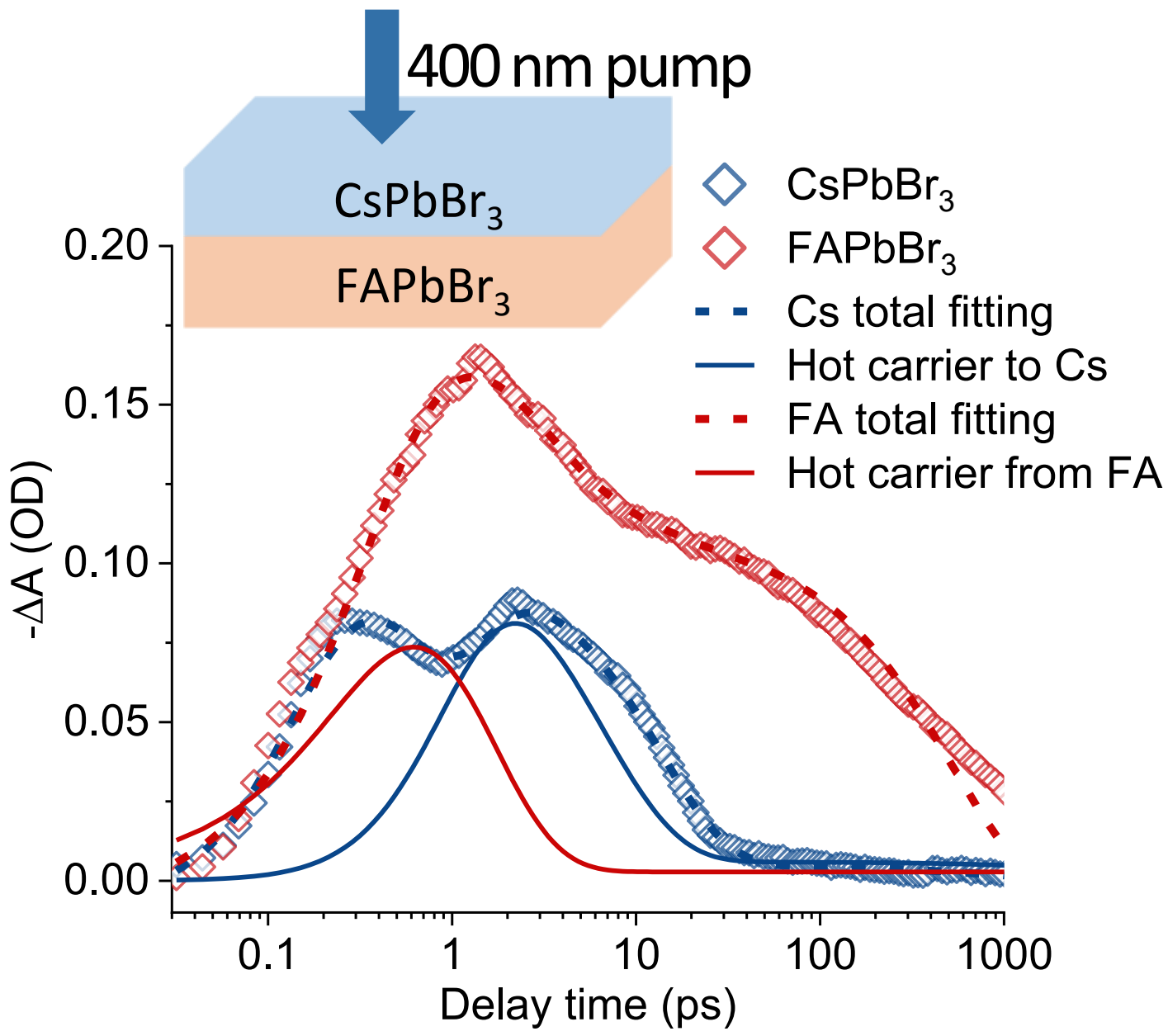

Fig. 3c, TA kinetics of laminated bilayer thin films. The pump laser (400 nm) was directed at $CsPbBr_3$ side. Global fitting was used to extract hot carrier transfer process in total TA signals (see details in Methods part). The dash line and solid line in (c) represent the fitting of total TA signals and the hot carrier transfer process, respectively.

We have ***added*** on the Page 10 of revised manuscript, that "As proposed in our earlier discussion of the hot carrier transfer mechanism (Fig. 1), when hot carriers are generated on both sides of the heterojunction, the differing cooling rates drive hot carriers toward the material with the faster cooling rate, such as $CsPbBr_3$. We globally fitted the TA kinetics at the $FAPbBr_3$ and $CsPbBr_3$ GSB positions using a sequential kinetic model to extract the hot carrier transfer dynamics (see Methods for details). In Fig. 3c, the red and blue solid lines represent hot carriers transferred out of $FAPbBr_3$ and those injected into $CsPbBr_3$, respectively. Critically, the blue component constitutes the dominant fraction of the total carriers present in

$CsPbBr_3$, confirming that hot carrier transfer is the primary source of population in the $CsPbBr_3$. At early times, hot carriers initially accumulate in the $FAPbBr_3$ phase due to the hot phonon bottleneck effect, reaching a maximum at ~0.6 ps, whereas hot carriers in $CsPbBr_3$ cool very rapidly (<0.2 ps). This population imbalance between these hot carrier levels in both materials drives directional hot carrier transfer from $FAPbBr_3$ to $CsPbBr_3$, resulting in a pronounced hot carrier population in $CsPbB_3$ that persists until ~2 ps and subsequently decays over approximately 10 ps. The fitted hot carrier transfer time constant and carrier decay rates are summarized in Table S2. Notably, the hot carrier population remains detectable in $CsPbBr_3$ for ~9.8 ps, which is considerably longer than the transfer out of $FAPbBr_3$ (~1.2 ps). This difference may be attributed to the finite transport time of hot carriers across the heterointerface."

We have ***added*** on the Page 11 of revised manuscript, that "After inserting the PMMA block layer, the GSB kinetics for both $CsPbBr_3$ and $FAPbBr_3$ (Fig. 3e, f) layers are close to those of the individual layers shown in Fig. 3a. These results further confirm that hot carrier transfer occurs within the laminated bilayers, and that the transfer is hindered when an intervening layer prevents direct interfacial interaction. Importantly, the hot-carrier transfer process persists for approximately 10 ps, which is fully consistent with our global fitting results (Fig. 3c)."

We ***added*** on the Page 11 of revised manuscript, that "Besides, as depicted in Fig. 1b, due to type II band alignment, in principle, the hole generated in $FAPbBr_3$ ~~would~~ may also jump to $CsPbBr_3$ VB maximum further increasing their carrier density. However, the conduction band offset (ΔCBM = 0.09 eV) in our $CsPbBr_3$/$FAPbBr_3$ heterojunction is substantially larger than the valence band offset (ΔVBM = 0.02 eV). Similar perovskite heterostructures have been identified, through both theoretical calculations and experimental measurements, as possessing a quasi-type-II alignment *(36-38)*. A quasi-type-II alignment is characterized by partial spatial separation between electron and hole wavefunctions, meaning that significant wavefunction overlap exists. Consequently, electron transfer across the interface is much more favourable than hole transfer. Although $CsPbBr_3$ has a high CBM

compared to that of $FAPbBr_3$, we did not observe the conventional carrier transfer from $CsPbBr_3$ to $FAPbBr_3$~~, likely due to fast carrier recombination in $CsPbBr_3$ (<100 ps) before carrier reaching interface.~~ Global fitting of the hot-carrier transfer dynamics reveals that both carrier cooling and hot-carrier injection into $CsPbBr_3$ are extremely fast (the transfer time constant is ~9.8 ps, while initial cooling occurs in <0.2 ps in $CsPbBr_3$). This enables efficient carrier accumulation in the $CsPbBr_3$ phase before any significant decay can take place. The TA decay kinetics further show that the carrier lifetime in $CsPbBr_3$ is only tens of ps (Table S2), which is much shorter than the hundreds of picoseconds observed in $FAPbBr_3$. Moreover, most conventional carrier transport across the bilayer heterostructure is reported to occur on timescales longer than 100 ps *(39-41)*. Any carriers that reach the conduction band edge of $CsPbBr_3$ recombine radiatively to contribute to optical gain before they can transfer back to $FAPbBr_3$. Thus, the hot-carrier (both electron and hole) transfer from slower-cooling $FAPbBr_3$ to fast-cooling $CsPbBr_3$ dominates while other processes (hole transfer from $FAPbBr_3$ to $CsPbBr_3$ and electron transfer from $CsPbBr_3$ to $FAPbBr_3$) are negligible under our experimental conditions (Fig. 1b)."

36. Zhang, X., Guo, Z., Li, R., Yu, J., Yuan, B., Chen, B., He, T. and Chen, R., Quasi-type II core–shell perovskite nanocrystals for improved structural stability and optical gain. *ACS Applied Materials & Interfaces*, 13(48), pp.58170-58178 (2021).
37. Imran, M., Peng, L., Pianetti, A., Pinchetti, V., Ramade, J., Zito, J., Di Stasio, F., Buha, J., Toso, S., Song, J. and Infante, I., Halide perovskite–lead chalcohalide nanocrystal heterostructures. *Journal of the American Chemical Society,* 143(3), pp.1435-1446 (2021).
38. Fang, Q., Shang, Q., Zhao, L., Wang, R., Zhang, Z., Yang, P., Sui, X., Qiu, X., Liu, X., Zhang, Q. and Zhang, Y., Ultrafast charge transfer in perovskite nanowire/2D transition metal dichalcogenide heterostructures. *The journal of physical chemistry letters*, 9(7), 1655-1662 (2018).
39. Fu, Y., Zheng, W., Wang, X., Hautzinger, M.P., Pan, D., Dang, L., Wright, J.C., Pan, A. and Jin, S., Multicolor heterostructures of two-dimensional layered halide perovskites that show interlayer energy transfer. *Journal of the*

American Chemical Society, 140(46), 15675-15683 (2018).

40. Li, W., Hu, L., Ma, J., Jiang, C., Zhang, S., Chen, Y., Hu, J., Liu, X., Wu, T. and Li, D., Enhancing self-trapped exciton emission via energy transfer in two-dimensional/quantum dot perovskite heterostructures. *ACS Photonics*, 9(6), 2008-2014, (2022).

41. C. Guo, J. Guo, W. Song, S. Peng, M. Sun, G. Xing, L. Yu, Atomic heterointerface engineering and nonequilibrium carrier dynamics for enhanced photocatalysis in halide perovskite/MoS_2 systems. *Angew. Chem. Int. Ed.*, 64, e202506436 (2025).

To quantitatively evaluate how hot-carrier transfer affects the lasing threshold, we ***performed*** additional rate-equation simulations on the bilayer heterojunction.

We have ***added*** on the Supporting Information of revised manuscript, that: "

Modeling of laser rate equation for the bilayer perovskite heterojunctions

To model the stimulate emission process in the bilayer perovskite heterojunctions, the following set of modified laser rate equations including the term for hot carrier transfer are used:

$$\frac{dn_{\mathrm{Cs}}}{dt} = G_{\mathrm{Cs}}(t) + \left(\frac{dn_{\mathrm{Cs}}}{dt}\right)_{\mathrm{trans}} - k_1 n_{\mathrm{Cs}} - k_2 n_{\mathrm{Cs}}^2 - k_3 n_{\mathrm{Cs}}^3 + (n_{\mathrm{Cs}} - n_{\mathrm{tr\_Cs}})\delta_{\mathrm{st}} v_g n_{\mathrm{p\_Cs}} \tag{1}$$

$$\frac{dn_{\mathrm{p\_Cs}}}{dt} = \Gamma \beta_{\mathrm{sp}} k_2 n_{\mathrm{Cs}}^2 + \Gamma(n_{\mathrm{Cs}} - n_{\mathrm{tr\_Cs}})\delta_{\mathrm{st}} v_g n_{\mathrm{p\_Cs}} - \frac{n_{\mathrm{p\_Cs}}}{\tau_{\mathrm{p\_Cs}}} \tag{2}$$

$$\frac{dn_{\mathrm{FA}}}{dt} = G_{\mathrm{FA}}(t) - \frac{n_{\mathrm{FA}}}{\tau_{\mathrm{trans}}} - \frac{n_{\mathrm{FA}}}{\tau_{\mathrm{FA}}} \tag{3}$$

where n, n_p, k_1, k_2, k_3, n_{tr}, σ_{st}, v_g, Γ, β_{sp}, τ_p, τ_{trans}, and τ_{FA} are the carrier density, photon density, 1st rate constant (5.6×10^8 s^{-1}) 2nd rate constant (1×10^{-9} cm^3 s^{-1}), 3rd rate constant (0.6×10^{-29} cm^6 s^{-1}), transparency carrier density (10^{18} cm^{-3}), stimulated emission cross-section (10^{-16} cm^2), group velocity (5.6×10^9 cm s^{-1}), modal confinement factor (0.56), spontaneous emission coupling factor (10^{-4}), photon escape time (100 ps), hot carrier transfer time constant (1 ps), and carrier lifetime of $FAPbBr_3$ perovskite, respectively. $G(t)$ is the Gaussian generation

function (FWHM = 270 fs), and the integral of $G(t)$ equals the initial carrier density ($\int G_{Cs}(t)dt + \int G_{FA}(t)dt = N_0$), which is varied from 10^{17} cm^{-3} to 10^{20} cm^{-3} during the modeling.

For the modeling of single layer perovskite, the generation laser rate equations (eqn. 1 and eqn. 2) are used with neglecting the hot carrier transfer term in eqn. 1. Upon numerically solving the rate equations, the time profile of photon density and L_{in}- L_{out} characteristics can be obtained. Then effect of hot carrier transfer in the bilayer perovskite heterojunction to the lasing performance can be theoretically evaluated via comparing the lasing threshold between the single layer perovskite and heterojunction."

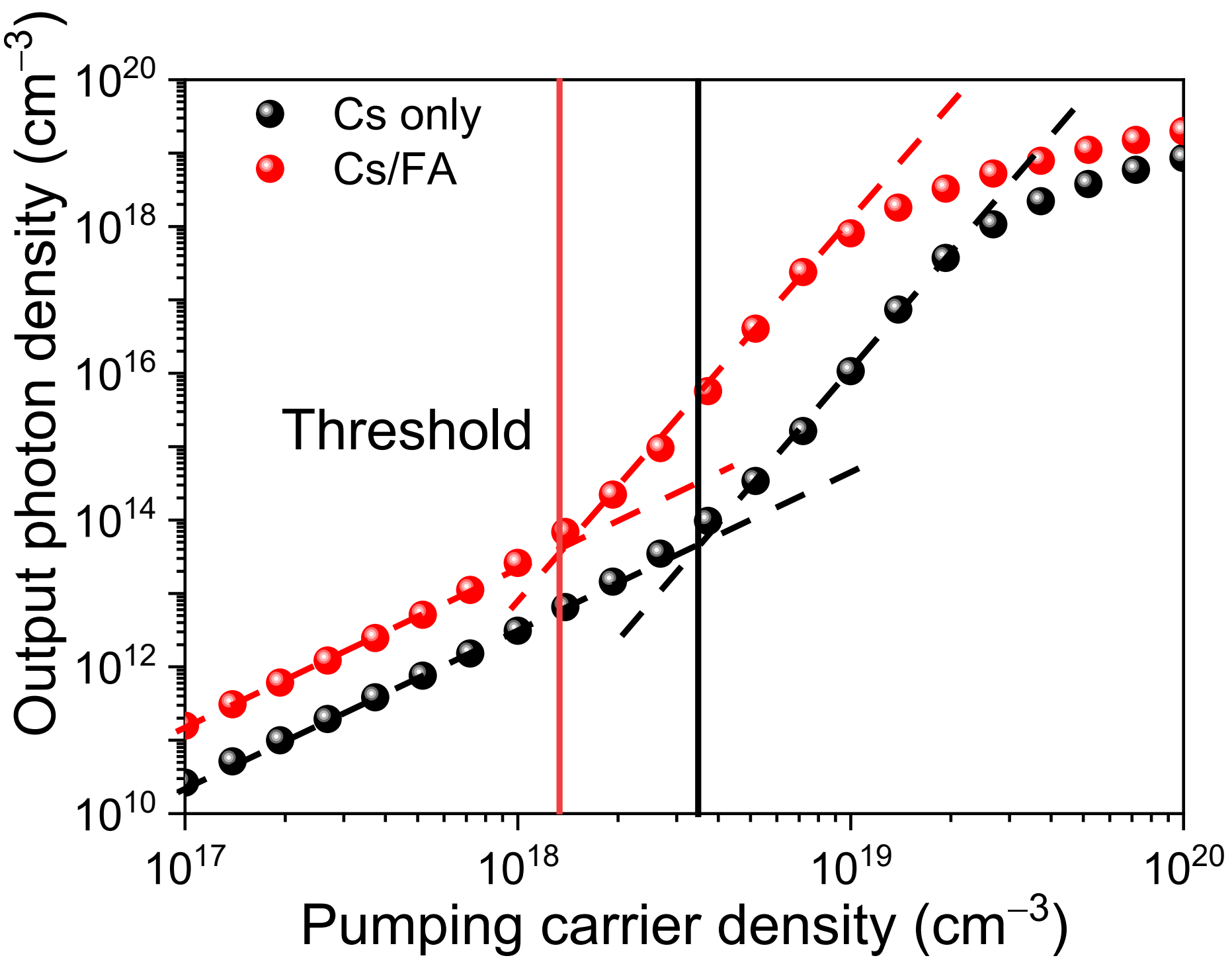

Figure. S8. Modelled L_{pump}-L_{out} characteristics for $CsPbBr_3$ and $CsPbBr_3/FAPbBr_3$ perovskite heterojunction via numerically solving the laser rate equations. The modeling results show a significant reduction in lasing threshold (3.7 × 10^{18} cm^{-3} for $CsPbBr_3$ and 1.2 × 10^{18} cm^{-3} for $CsPbBr_3/FAPbBr_3$) via introducing a hot carrier transfer term into the laser rate equations.

We have ***added*** on the Page 12 of revised manuscript, that: "These findings further confirm the occurrence of hot carrier transfer from $FAPbBr_3$ to $CsPbBr_3$ within the laminated structures, which markedly enhances the carrier density at $CsPbBr_3$ excited states and facilitates their population inversion and optical gain, thereby contributing to the observed large reduction in ASE threshold, consistent with our simulation results (Fig. S8, see details in Supporting Information)."

Then, what I find not well explained is why the bilayer results in a low but also wavelength independent laser threshold. The laser threshold is proportional to the ratio between the time carriers injected in the electronic state take to relax to the bottom(top) of the conduction(valence) band, which includes the time carrier will take to transfer from one layer to the other and the time carriers take to cool and the time such state takes to get depleted. If we assume that the depletion time does not change I still expect an excitation energy dependence.

Our Response: We thank the reviewer for this incisive comment. We agree that a simple model where threshold scales only with cooling time is incomplete, and we appreciate the opportunity to clarify why the observed threshold shows only a weak dependence on pump wavelength, and why this is actually consistent with, and indeed supports, our proposed hot-carrier transfer mechanism.

First, we confirm the reviewer's expectation for single-layer films. When comparing higher-energy excitation (360 nm and 400 nm) to lower-energy excitation (460 nm), both pure $CsPbBr_3$ and pure $FAPbBr_3$ exhibit a clear decrease in ASE threshold at 460 nm (Fig. 3i). This is consistent with previous studies (*Nat Commun 6, 8056 (2015).; Advanced Materials 35, 2208293 (2023)*), which report lower thresholds under near-resonant pumping due to accelerated carrier cooling and reduced energy loss to heat. Thus, the reviewer is correct: for single layers, there is a clear excitation energy dependence.

For the laminated bilayer, the situation is more complex because two competing effects operate simultaneously as the pump wavelength increases: an increased

carrier cooling rate, which tends to lower the threshold in both $CsPbBr_3$ and $FAPbBr_3$, and a decreased proportion of transferred hot carriers, which tends to raise (lower) the threshold in $CsPbBr_3$ ($FAPbBr_3$). Thus, as the pump wavelength increases (i.e., photon energy decreases), fewer hot carriers are generated, so the transfer efficiency from $FAPbBr_3$ to $CsPbBr_3$ declines. This effect opposes the beneficial cooling effect for $CsPbBr_3$ but reinforces it for $FAPbBr_3$, consistent with our experiment observations.

We ***added*** our discussion on the Page 11 of revised manuscript, that "To further elucidate the role of hot carrier transfer, we investigate the ASE thresholds of laminated samples by varying the pump wavelength. When comparing higher energy excitations at 360 nm and 400 nm to lower energy excitation at 460 nm, both pure $CsPbBr_3$ and $FAPbBr_3$ films exhibit a decrease in their ASE thresholds at 460 nm (Fig. 3i). These findings are consistent with previous studies reporting lower thresholds under near-resonant pumping conditions due to accelerated carrier cooling and reduced energy loss to heat. (*42–44*). In our work, however, we deliberately extract hot carriers from the slow-cooling material ($FAPbBr_3$) and transfer them to the fast-cooling material ($CsPbBr_3$) to further enhance the carrier population in $CsPbBr_3$. Consequently, as the pump energy decreases – and thus the population of hot carriers diminishes – the ASE threshold reduction induced by hot-carrier transfer from pure $CsPbBr_3$ to the planar heterojunction becomes less pronounced: the reduction factor is 4.5 at 400 nm but only ~2.5 at 460 nm. ~~Interestingly, as the pumping energy decreases, leading to less hot carrier populations, the hot carrier transfer-induced ASE threshold reduction from pure $CsPbBr_3$ to the planar heterojunction becomes less significant (4.5 times for 400 nm and ~2.5 times for 460 nm).~~ Furthermore, for the laminated $FAPbBr_3$ samples, we observe a dramatic decrease in the ASE threshold (from ~10 to ~3 μJ/cm²) when the pump wavelength is increased from 360 nm to 460 nm. In contrast, the threshold of laminated $CsPbBr_3$ samples shows only a weak dependence on pump wavelength (varying from ~3 to ~2 μJ/cm²). This behavior is fully consistent with our proposed hot-carrier transfer mechanism. As the pump wavelength increases, two competing effects come into play: an increased carrier cooling rate, which tends to

lower the threshold in both $CsPbBr_3$ and $FAPbBr_3$, and a decreased proportion of transferred hot carriers, which tends to raise (lower) the threshold in $CsPbBr_3$ ($FAPbBr_3$). The interplay of these effects results in a weak wavelength dependence for the laminated $CsPbBr_3$ samples, where the two effects partially cancel. In contrast, the laminated $FAPbBr_3$ samples exhibit a much stronger threshold reduction with increasing wavelength, driven by the combined influence of an accelerated cooling rate and a reduced loss of carriers through transfer. This result further supports the conclusion that hot carrier transfer is the primary mechanism responsible for increasing carrier population and lowering the ASE threshold in the perovskite heterojunctions."

Then, how the authors can exclude the role of defects at the interfaces, e.g. the formation of a sort of three states system, which would also help the population inversion, since the heterojunction is not "clean" as one would find in epitaxially grown systems?

To conclude I do not find the manuscript convincing in its current form.

Our Response: We thank the reviewer for raising this critical point. We recognize that our manuscript did not sufficiently clarify the intrinsic level structure of our heterojunction, nor did we provide direct experimental controls to rule out defect-mediated effects. We have now addressed this concern in two ways: (1) by clarifying that the heterostructure itself constitutes a multi-level system even in the absence of defects, and (2) by proposing and, in the revised manuscript, including new control experiments that distinguish hot-carrier transfer from defect-assisted processes.

The heterojunction inherently provides a multi-level system. Even without any interface defects, our $CsPbBr_3$/$FAPbBr_3$ bilayer forms an intrinsic multi-level system due to:

The distinct bandgaps of the two materials ($CsPbBr_3$ ~2.40 eV, $FAPbBr_3$ ~2.33 eV)

The type-II band alignment ($\Delta CBM = 0.09$ eV, $\Delta VBM = 0.02$ eV)

The hot carrier states accessed under high-fluence or high-energy excitation Thus, the three-level laser picture we propose relies on the band structure of the heterojunction, not on defect states. Defects are not required to explain the observed population inversion or threshold reduction.

We further ***performed*** control experiments to rule out the possible contributions from defect states. Notably, we find that the PLQE values for the laminated structure does not decrease below the single layer values (revised Fig. S7), excluding an impact from potential defects at the interface to the light emission process.

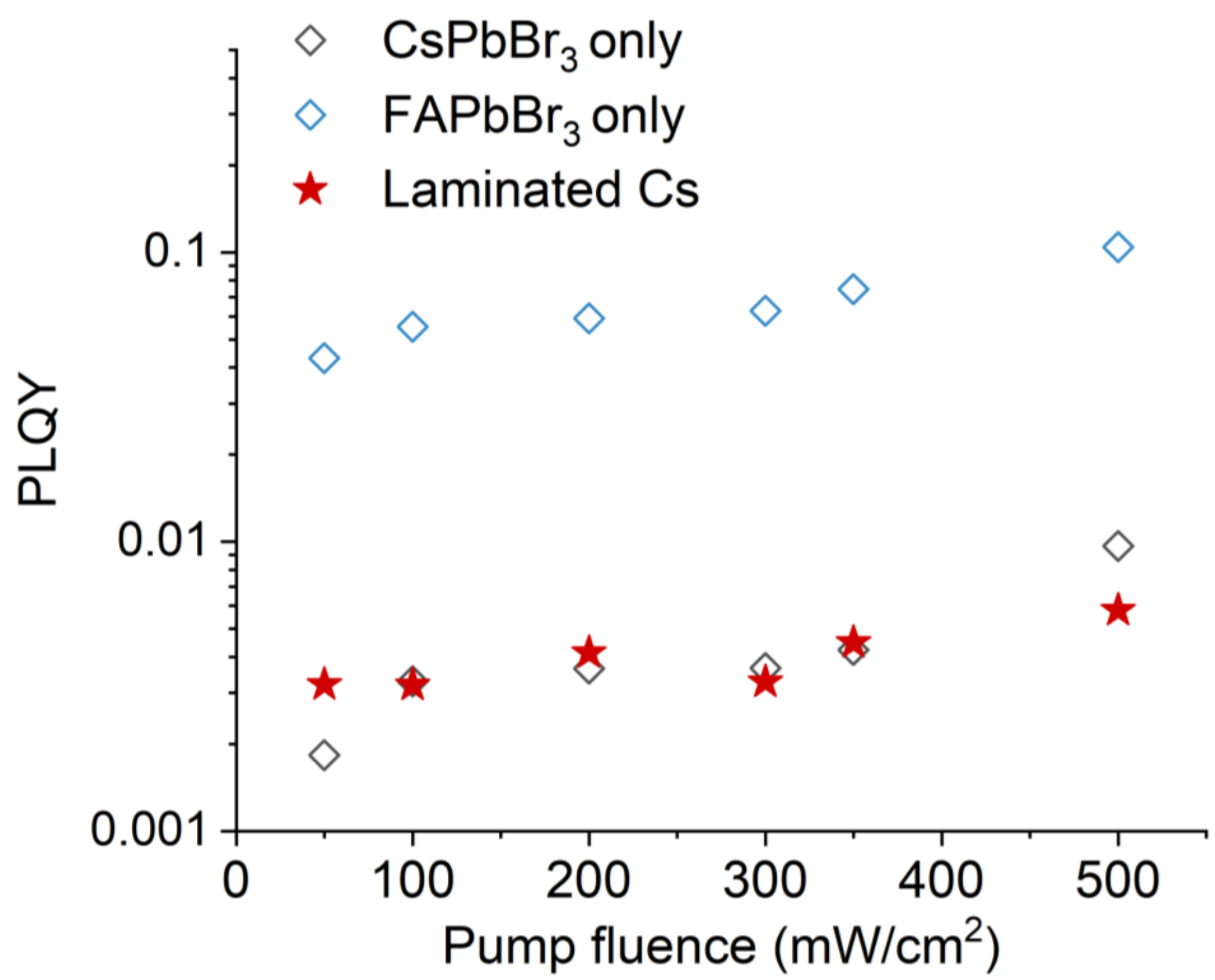

Figure S7. Comparison of photoluminescence quantum yield (PLQY) results before and after lamination process ~~$CsPbBr_3$ and $FAPbBr_3$ films~~. The samples were excited with a 400 nm continuous wave laser, and a ND filter wheel was used to control the excitation fluences. $FAPbBr_3$ films exhibit a PLQY that is at least ten times higher than that of $CsPbBr_3$ films at all pump powers. Importantly, after the lamination process, the PLQY of the $CsPbBr_3$ films remains almost unchanged compared to their pre-lamination values, further confirming that the defect density

is not altered by lamination.

We have ***added*** the discussion on the Page 12 of revised manuscript, that "Recent studies have suggested that defect states could also form a three-level system, which might facilitate population inversion *(45-47)*. In our work, however, the heterojunction itself inherently provides a multi-level structure. The three-level laser picture we propose is grounded in the band alignment and hot carrier transfer in the $CsPbBr_3$/$FAPbBr_3$ heterojunction, not in the presence of defect states. Consequently, defects are not required to explain the observed population inversion or the reduction in lasing threshold. To further rule out possible contributions from defect states, we compared the excited-state carrier dynamics before and after the lamination process. In both TA and time-resolved photoluminescence (TRPL) spectra, we observed blue-shifted signals on the high-energy tail at early times after lamination (Fig. S9 and S10), indicating the contribution of hot carriers transferred into the $CsPbBr_3$ side *(48-50)*. At later times (beyond 1 ns), both TA and TRPL spectra overlapped well on the low-energy tail, confirming that no additional trap states were introduced and that existing trap states were not strongly passivated by lamination.

Notably, under equivalent low carrier generation densities (Fig. S9c), the $CsPbBr_3$ ground-state bleach (GSB) signals before and after lamination exhibited similarly rapid decays over the first few picoseconds – a feature typically attributed to trap-mediated recombination *(51, 52)* – indicating comparable trap densities. Moreover, at both low and high carrier generation densities, the $CsPbBr_3$ GSB decay kinetics remained nearly identical before and after lamination, further demonstrating that the defect density is essentially unchanged by the lamination process. Consistently, after lamination, the $CsPbBr_3$ PL decay showed a prolonged lifetime during the initial 4 ns (Fig. 10c and f), which we attribute to fast hot-carrier transfer from the $FAPbBr_3$ layer. Beyond 4 ns, the decay kinetics before and after lamination became almost overlapped, confirming that the density of long-lived trap states remains unaffected *(53)*. Further, the PLQY of $CsPbBr_3$ films remained nearly unchanged after lamination compared to their pre-lamination values (Fig.

S7). Taking together, all these results demonstrate that the lamination process does not significantly alter the defect density in $CsPbBr_3$. Therefore, the dominant mechanism responsible for reducing the ASE threshold in the laminated heterojunctions is hot-carrier transfer driven by cooling-rate gradients, not defect-mediated processes."

45. Wu, C. S. J., Wong, E. L., Li, H., Jiménez-López, J., Lin, C. K., Hsu, H. C., & Petrozza, A. Impact of Nano-Scale Defects on the Macroscopic Amplified Spontaneous Emission in Polycrystalline Perovskite Thin-Films. Advanced Materials, 38(24), e16903 (2026).
46. Zhang, L., Wang, Y., Chu, A. *et al.* Facet-selective growth of halide perovskite/2D semiconductor van der Waals heterostructures for improved optical gain and lasing. *Nat Commun* **15**, 5484 (2024).
47. S. Huang, Z. Shen, Y. Liao, Z. Liu, Z. Hu, Q. Li, Z. Zhang, S. Dong, J. Luo, J. Du, J. Tang, Y. Leng, Water-Resistant Subwavelength Perovskite Lasing from Transparent Silica-Based Nanocavity. Adv. Mater., 35, 2306102 (2023).
48. Righetto, M., Lim, S.S., Giovanni, D. *et al.* Hot carriers perspective on the nature of traps in perovskites. *Nat Commun* **11**, 2712 (2020).
49. Ye, J., Mondal, N., Carwithen, B.P. *et al.* Extending the defect tolerance of halide perovskite nanocrystals to hot carrier cooling dynamics. *Nat Commun* **15**, 8120 (2024).
50 L. Zhang, W. Xi, H. Zhang, et al. Efficient Hot Hole Extraction in Halide Perovskite/2D Semiconductor van der Waals Heterostructures. *Adv. Funct. Mater.*36, 22, e17023 (2026).
51. Fan, H., Li, F., Wang, P. *et al.* Methylamine-assisted growth of uniaxial-oriented perovskite thin films with millimeter-sized grains. *Nat Commun* **11**, 5402 (2020).
52. Wang, X., Jin, L., Sergeev, A., Liu, W., Gu, S., Li, N., Fan, K., Chen, S.C., Wong, K.S., Sun, X. and Zhao, N., Quasi-2D Dion-Jacobson phase perovskites as a promising material platform for stable and high-performance lasers. *Science Advances*, 9(43), eadj3476 (2023).
53. Fang, HH., Adjokatse, S., Shao, S. et al. Long-lived hot-carrier light emission

and large blue shift in formamidinium tin triiodide perovskites. Nat Commun 9, 243 (2018).

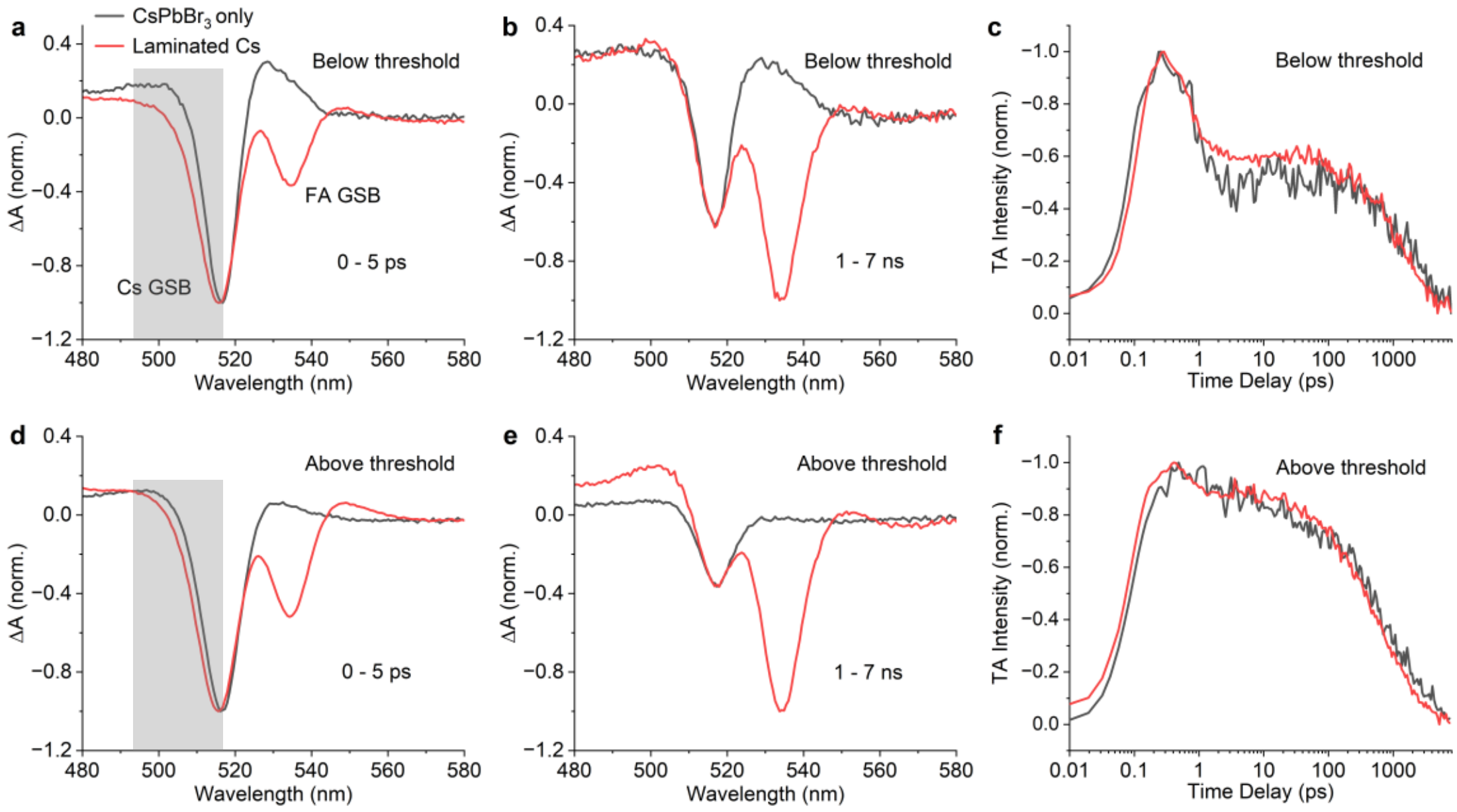

Figure S9. Comparison of transient absorption spectra before and after lamination process. a-c, Low carrier density generation regime (below ASE threshold), TA spectra (a, 0-5 ps; b, 1-7 ns) and kinetics of $CsPbBr_3$ GSB (c) before and after lamination. d-f, High carrier density generation regime (above ASE threshold), TA spectra (a, 0-5 ps; b, 1-7 ns) and kinetics of $CsPbBr_3$ (c) before and after lamination. Notably, at equivalent carrier generation densities, the $CsPbBr_3$ GSB decay kinetics exhibit nearly identical behavior before and after lamination, indicating that the defect density remains essentially unchanged by the lamination process.

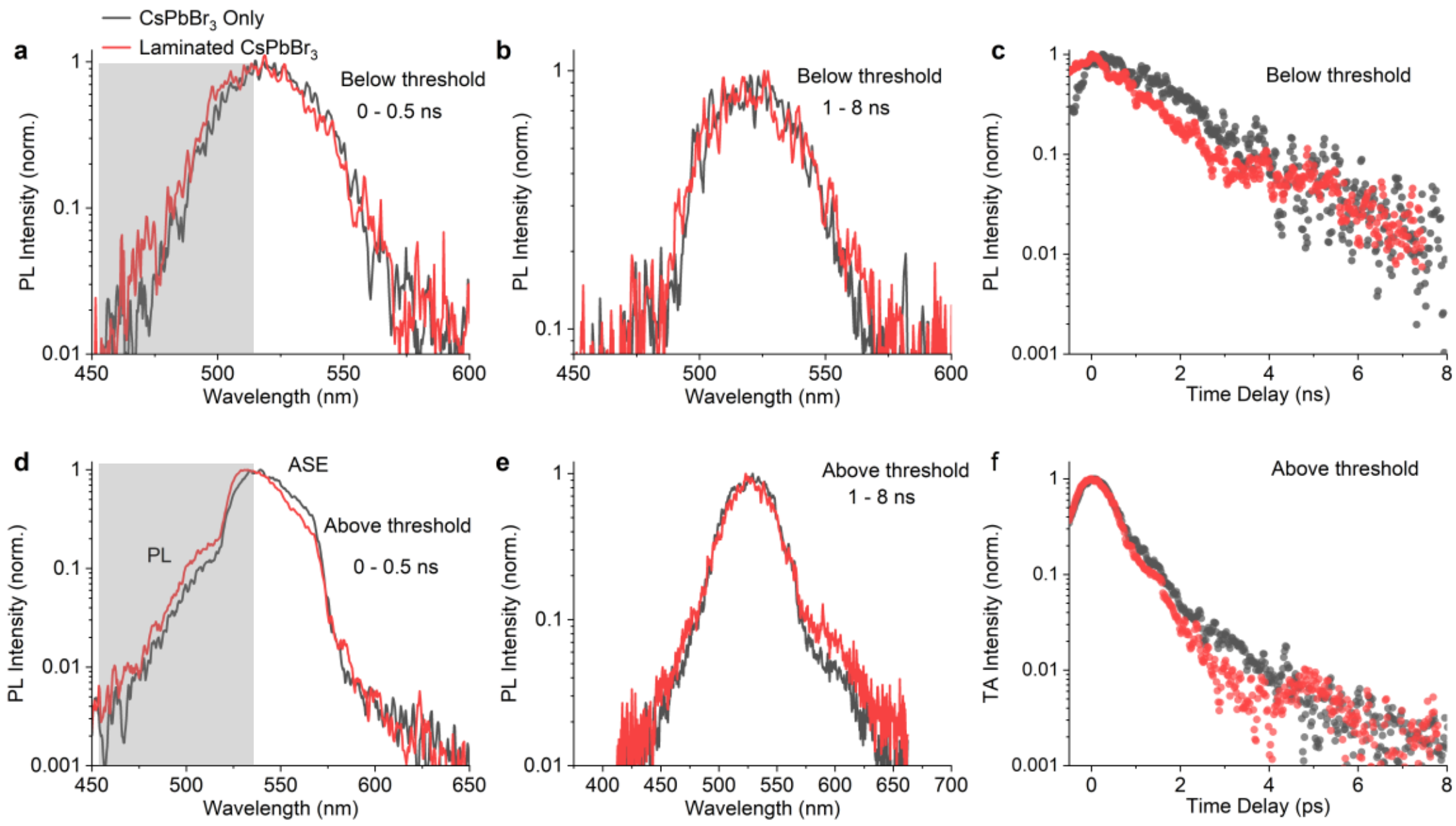

Figure S10. Comparison of time-resolved PL spectra before and after lamination process. a-c, With low excitation fluence (below ASE threshold), PL spectra (a, 0-5 ns; b, 1-8 ns) and decay kinetics of $CsPbBr_3$ (c) before and after lamination. d-f, With high excitation fluence (above ASE threshold), PL spectra (a, 0-5 ns; b, 1-8 ns) and decay kinetics of $CsPbBr_3$ (c) before and after lamination. Time resolved PL spectra were obtained by streak camera. After lamination, the $CsPbBr_3$ PL decay exhibits a prolonged lifetime during the initial 4 ns, which we attribute to fast hot-carrier transfer from the $FAPbBr_3$ layer. Beyond 4 ns, the decay kinetics before and after lamination become nearly overlapped, confirming that the lamination process does not alter the density of long-lived trap states.

We further **compared** the ASE thresholds of laminated $CsPbBr_3/FAPbBr_3$ heterojunction and a control sample with a PMMA interlayer inserted between the two perovskite layers. Both samples underwent the identical lamination process; however, the PMMA layer blocks hot-carrier transfer, thereby disabling the transfer pathway.

We have ***plotted*** below and in revised manuscript Fig. S12 all ASE threshold results with error bars indicated.

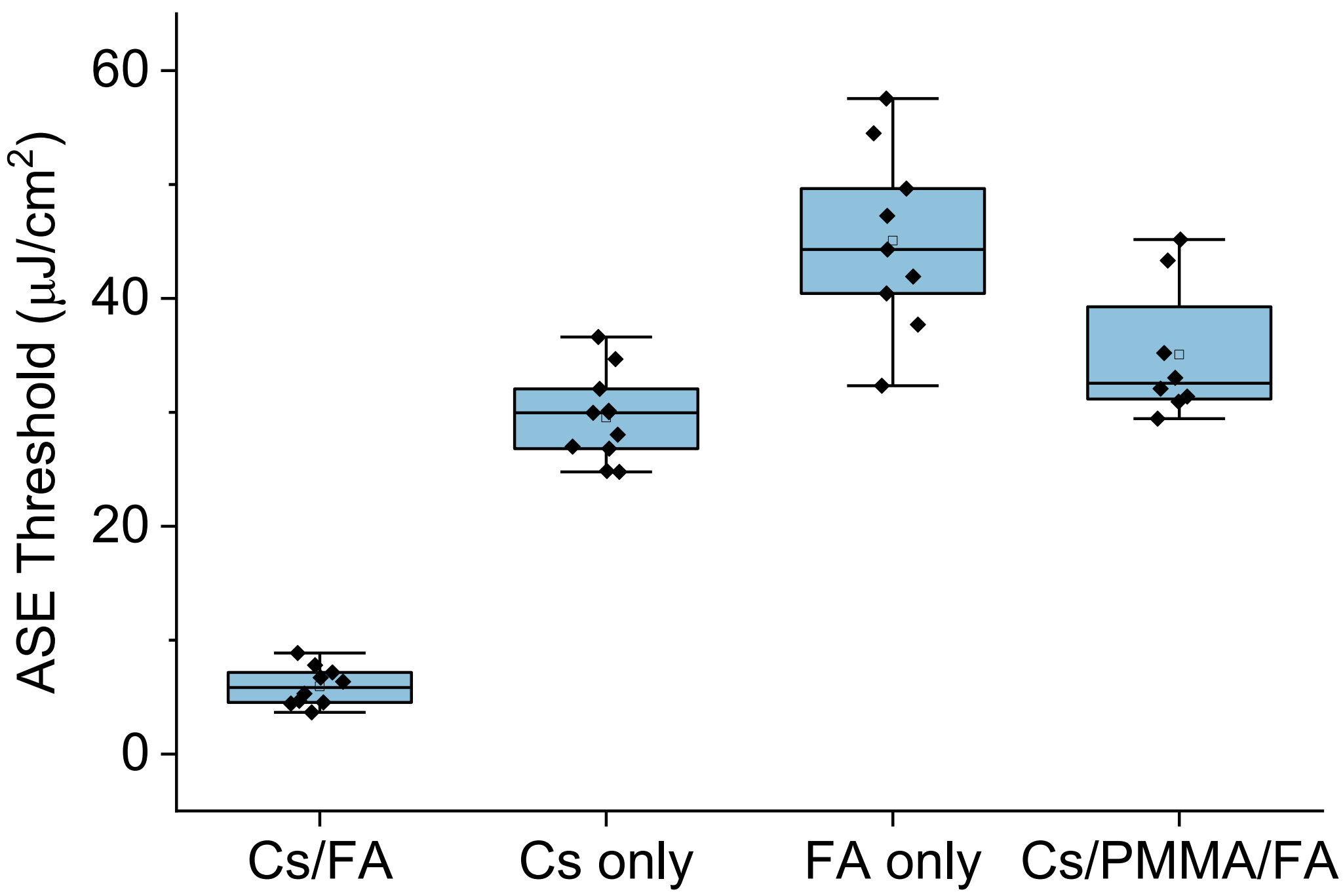

Figure S12. Comparison of amplified spontaneous emission (ASE) thresholds. ASE thresholds were measured for the laminated $CsPbBr_3$/$FAPbBr_3$ heterojunction, single-layer $CsPbBr_3$, single-layer $FAPbBr_3$, and a control sample with a PMMA interlayer inserted between the two perovskite layers during the lamination process. Each condition was measured on at least five devices (individual data points shown as solid squares). A 355 nm nanosecond pulsed laser was used for excitation.

We have ***added*** on the Page 15 of revised manuscript, that "Using 355 nm nanosecond pulsed excitation (1 ns pulse width, 1 kHz repetition rate) laminated $CsPbBr_3$ samples exhibit a substantially lower ASE threshold (~4 μJ/cm²) compared to individual samples (Fig. 4a). Statistical analysis across multiple devices confirms the reproducibility of this threshold reduction (Fig. S12). We further compared the ASE thresholds of laminated CsPbBr3/FAPbBr3 heterojunction and a control sample with a PMMA interlayer inserted between the two perovskite layers. Both samples underwent the identical lamination process; however, the PMMA layer blocks hot-carrier transfer, thereby disabling the hot transfer pathway. Consequently, the

control sample exhibited no ASE threshold reduction relative to single-layer $CsPbBr_3$ (Fig. S12), confirming that the threshold reduction in the laminated heterojunction is primarily attributable to hot-carrier transfer."

We hope that with these clarifications and additional experiments, the reviewer finds the manuscript substantially more convincing. We thank the reviewer for pushing us to provide this rigorous control evidence.

Reviewer #3 (Remarks to the Author):

This paper investigates the ultrafast directional transport mechanism of hot carriers in perovskite heterojunctions. By designing a $CsPbBr_3$/$FAPbBr_3$ bilayer bulk heterojunction and combining ultrafast spectroscopy with device fabrication, the study demonstrates a cooling-rate-gradient-driven directional transfer of hot carriers, which enables the successful realization of a low-threshold perovskite vertical-cavity surface-emitting laser (VCSEL). ***The research is positioned at the forefront of optoelectronic devices, featuring an innovative experimental design and robust data support. Its conclusions provide valuable guidance for the development of perovskite lasers, photodetectors, and other related devices. Overall, the paper exhibits a clear logical structure, well-organized figures and tables, and fluent writing.*** However, further refinement/major reversion is needed in the mechanistic interpretation, experimental details, data comparison, and stability analysis, and some conclusions require additional validation.

Our Response: We thank the reviewer for acknowledging the interest and the importance of the current work.

1. Several issues regarding Figure 2 require attention. First, the scale bars are missing in Figures 2d, 2e, 2g, and 2h, which are essential for interpreting the morphological features. Second, the substrate used in Figures 2d and 2e is ITO, whereas the $CsPbBr_3$/$FAPbBr_3$ bilayer in Figure 2f is deposited on glass. Given that ITO and glass exhibit different surface chemistries and physical properties—factors that can

significantly influence the crystallization process of perovskites—this inconsistency in substrate selection necessitates clarification. Additionally, the morphologies of $CsPbBr_3$ shown before (Figures 2g) and after (Figure 2i) the lamination process appear markedly different. The underlying mechanism driving this morphological evolution should be discussed to validate the integrity of the lamination procedure. The horizontal axis label in Figure 3h, "Pump power (μJ/cm²)," is a misnomer; the correct term should be "Pump fluence."

Our Response: We thank the reviewer for carefully identifying these issues. We have now addressed each one in the revised manuscript, as detailed below.

We have ***added*** scale bars to all SEM images in Figure 2 (panels d, e, g, h). The scale bars are now clearly indicated in the figure and the caption.

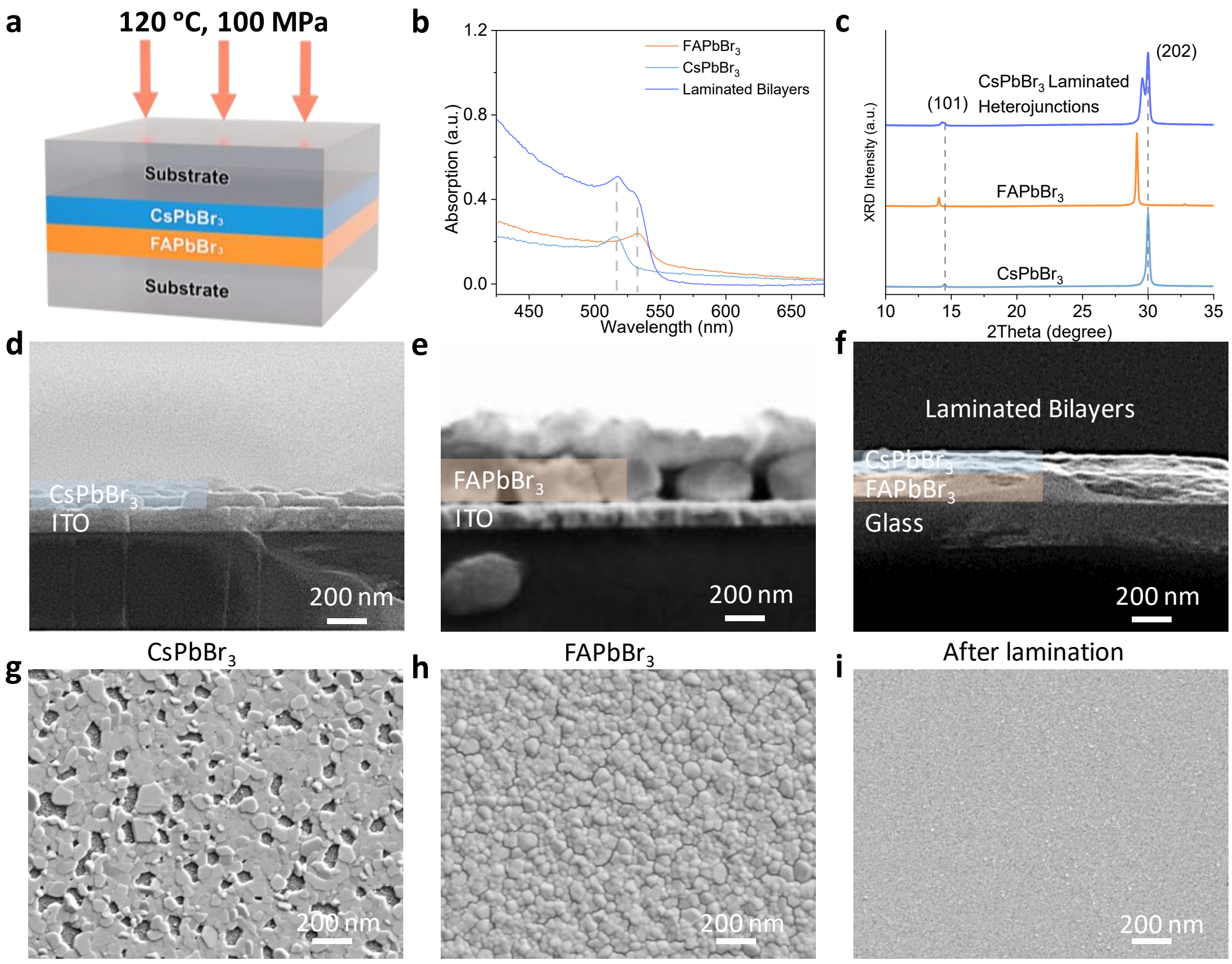

Figure 2. Formation of bilayer bulk three-dimensional perovskite heterojunctions. **a**, Schematic illustration of the lamination process used to fabricate bilayer perovskite heterojunctions. **b**, Absorption spectra of $CsPbBr_3$, $FAPbBr_3$, and laminated bilayer films. **c**, X-ray diffraction (XRD) patterns of

$CsPbBr_3$, $FAPbBr_3$, and $CsPbBr_3$ films after lamination processes. **d-f**, Cross-sectional SEM images obtained from $CsPbBr_3$ (d), $FAPbBr_3$ (e), and laminated bilayer films (f). **g-i**, Top-view SEM images of $CsPbBr_3$ (g), $FAPbBr_3$ (h), and $CsPbBr_3$ films after lamination process (i).

For SEM measurements using the ITO and glass substrates, we thank the reviewer for pointing out this inconsistency, and we apologize for not providing a clear explanation in the original manuscript. The choice of substrate was dictated by practical considerations for cross-sectional SEM imaging of the delicate laminated bilayer, not by any intended difference in film growth.

In the lamination process, a $CsPbBr_3$ film and a $FAPbBr_3$ film are pressed face-to-face. After lamination, the two substrates must be separated to expose the bilayer interface for cross-sectional imaging. We found that the perovskite films adhere more strongly to glass than to ITO. Therefore, when we laminate a $CsPbBr_3$ film on glass with a $FAPbBr_3$ film on ITO (or vice versa), and then separate the two substrates, the bilayer structure remains intact on the glass side, while the ITO substrate detaches cleanly. This allowed us to obtain a clean cross-section of the intact $CsPbBr_3$/$FAPbBr_3$ bilayer for SEM imaging (Fig. 2f). In contrast, if both substrates were ITO, the bilayer often tore or delaminated during separation, preventing reliable cross-sectional imaging.

We acknowledge that the growth morphology of each individual perovskite layer may differ slightly between glass and ITO. However, for the purpose of Figure 2f, the key information is the existence of a continuous bilayer with a well-defined interface, not the absolute grain size or roughness of the individual layers.

We have ***added*** on the Method section of revised manuscript, that "Field emission top-view and cross-section SEM images of perovskite films were taken with a ZEISS Supra60 VP scanning electron microscope. For cross-sectional SEM imaging of the laminated bilayer, the $CsPbBr_3$ and $FAPbBr_3$ films were deposited on glass and ITO, respectively. After lamination, the two substrates were separated; the bilayer

adhered preferentially to the glass side, enabling clean cross-sectional imaging."

The reviewer rightly notes that the top-view morphology of $CsPbBr_3$ changes dramatically from a rough, partially covered film (Fig. 2g) to a smooth, dense, ultra-small-grain film (Fig. 2i) after lamination. We have now provided a clear explanation of this evolution together with control experiments.

We have ***added*** on the Page 7 of revised manuscript, that "Notably, prior to lamination, both $CsPbBr_3$ and $FAPbBr_3$ exhibit only partial coating of the substrate, while after lamination, additional heat and pressure drive the recrystallization of perovskite films, resulting in flat and uniform bilayer films. The same trends were observed in top-view SEM images (Fig. 2g-i). After lamination, $CsPbBr_3$ films become very uniform and smooth, with grain size below 10 nm resolution and improved surface roughness. The lamination process applies elevated temperature (120 °C) and high pressure (100 MPa) for 5 min. Under these conditions, the perovskite layer undergoes pressure-induced recrystallization. The combined heat and pressure promote atomic rearrangement, grain rotation, and coalescence, resulting in a much smoother, denser film with grain sizes below resolution (Fig. 2g-i). Similar recrystallization phenomena have been reported in laminated perovskite films by several groups *(27–30)*. Control experiments reveal that hot pressing a $CsPbBr_3$ film against a silicon wafer (i.e., without a second perovskite layer) improves its surface roughness, but the smoothing effect is substantially weaker than that observed after lamination with a $FAPbBr_3$ layer (Fig. S2). Additionally, lamination of thinner films produces a less pronounced morphological change. These observations indicate that the dramatic transformation is driven by recrystallization at the interface when two thick perovskite films are brought into intimate contact under heat and pressure. Importantly, this recrystallization does not degrade their structure and optical properties; as shown in Fig. 2b and 2c, the absorption spectrum and XRD of perovskite films are unchanged after lamination."

27. R. Schmager, J. Roger, J. A. Schwenzer, F. Schackmar, T. Abzieher, M. Malekshahi Byranvand, B. Abdollahi Nejand, M. Worgull, B. S. Richards, U. W.

Paetzold, Laminated perovskite photovoltaics: enabling novel layer combinations and device architectures. *Adv. Funct. Mater.* 30, 1907481 (2020).

28. J. Roger, L. K. Schorn, M. Heydarian, A. Farag, T. Feeney, D. Baumann, H. Hu, F. Laufer, W. Duan, K. Ding, A. Lambertz, P. Fassl, M. Worgull, U. W. Paetzold, Laminated monolithic perovskite/silicon tandem photovoltaics. *Adv. Energy Mater.* 12, 2200961 (2022).
29. Y. Li, J. Roger, I. Allegro, J. C. Fischer, Q. Jin, U. Lemmer, I. A. Howard, U. W. Paetzold, Lasing from laminated quasi-2D/3D perovskite planar heterostructures. *Adv. Funct. Mater.* 32, 2200772 (2022).
30. S. P. Dunfield, D. T. Moore, T. R. Klein, D. M. Fabian, J. A. Christians, A. G. Dixon, B. Dou, S. Ardo, M. C. Beard, S. E. Shaheen, J. J. Berry, M. F. A. M. Van Hest, Curtailing perovskite processing limitations via lamination at the perovskite/perovskite interface. *ACS Energy Lett.* 3, 1192–1197 (2018)

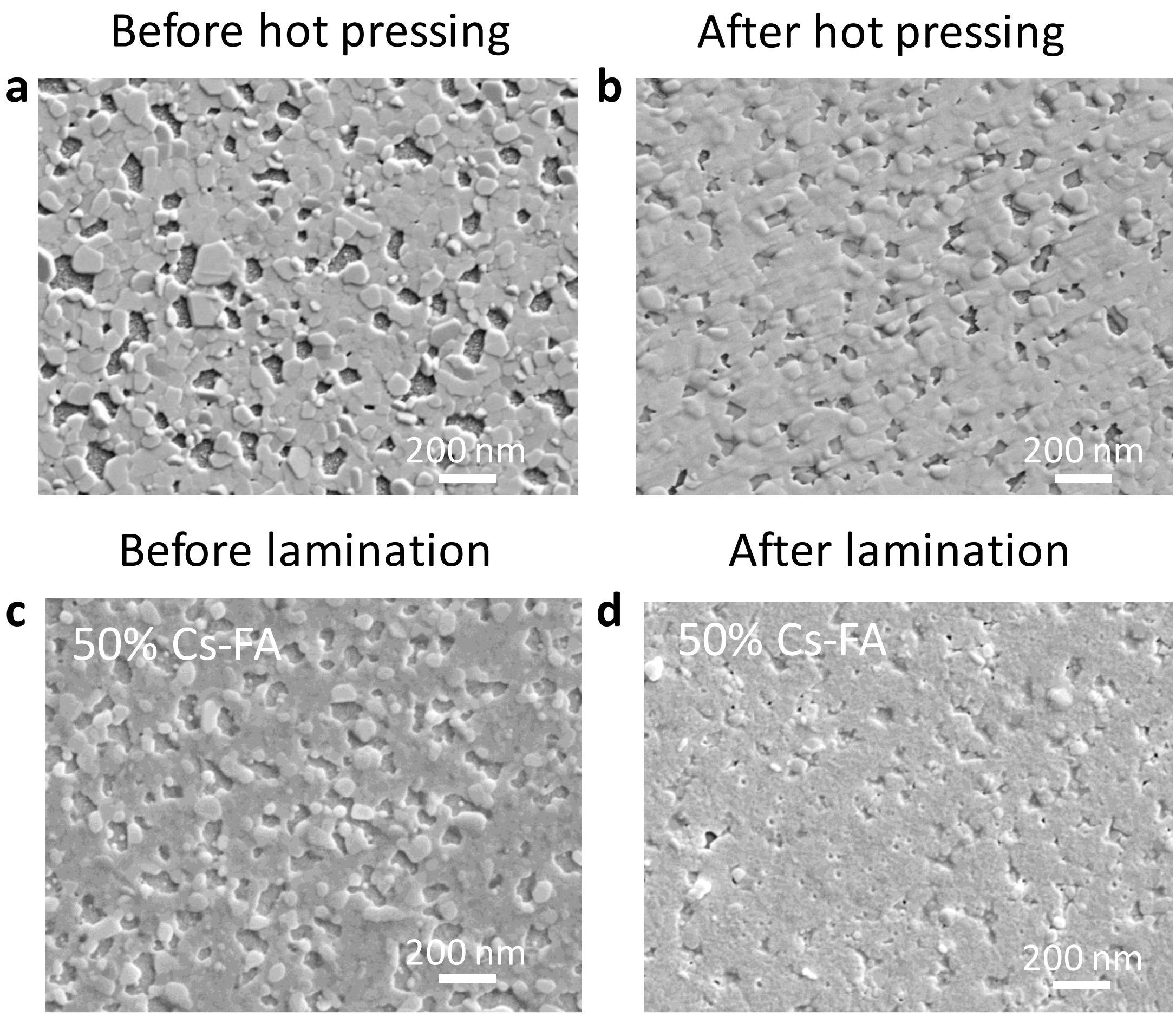

Figure S2. Top-view SEM images of $CsPbBr_3$ films under different processing conditions. a, b, $CsPbBr_3$ before and after hot pressing process. A clean silicon wafer was used to press on top of $CsPbBr_3$ films under the same pressure and temperature conditions as lamination (i.e., without a second perovskite layer). Hot pressing reduces surface roughness, but the improvement is moderate. c, d, $CsPbBr_3$ film with reduced thickness (50% of the original spin-coating solution concentration) before after lamination with a $FAPbBr_3$ (reduced thickness, 50% of the original spin-coating solution concentration). The morphological change is less pronounced compared to the standard-thickness bilayer, indicating that film thickness plays a critical role in the recrystallization process.

We agree with the reviewer that the horizontal axis label 'Pump power (μJ/cm^2)' in Fig. 3h is a misnomer; the correct term should be 'Pump fluence (μJ/cm^2) '.

We have ***changed*** the label to "Pump fluence (μJ/cm^2)" in the revised manuscript.

2. The energy barrier and kinetic details of the hot carrier transfer process require further clarification. Although the paper demonstrates that hot carrier transfer from $FAPbBr_3$ to $CsPbBr_3$ is driven by differences in cooling rates, it does not specify the energy barriers at the heterojunction interface (e.g., the transfer barriers for electrons and holes), nor does it provide a quantitative analysis of the transfer time constants. For instance, the transfer is only described as occurring on a "picosecond" timescale, without precise values. To address this, it is recommended to extract the rate constants of hot carrier transfer by fitting transient absorption (TA) kinetic data, and to supplement the energy band diagram with a quantitative determination of the barrier heights. Such additions would significantly enhance the understanding of the transfer kinetics.

Our Response: We agree that quantitative kinetic analysis is essential. We will extract transfer time constants from our TA data and provide energy barrier information.

We have **modified** the scheme in Fig 1 to quantitatively show the energy barrier between $CsPbBr_3$ and $FAPbBr_3$ CBM and VBM.

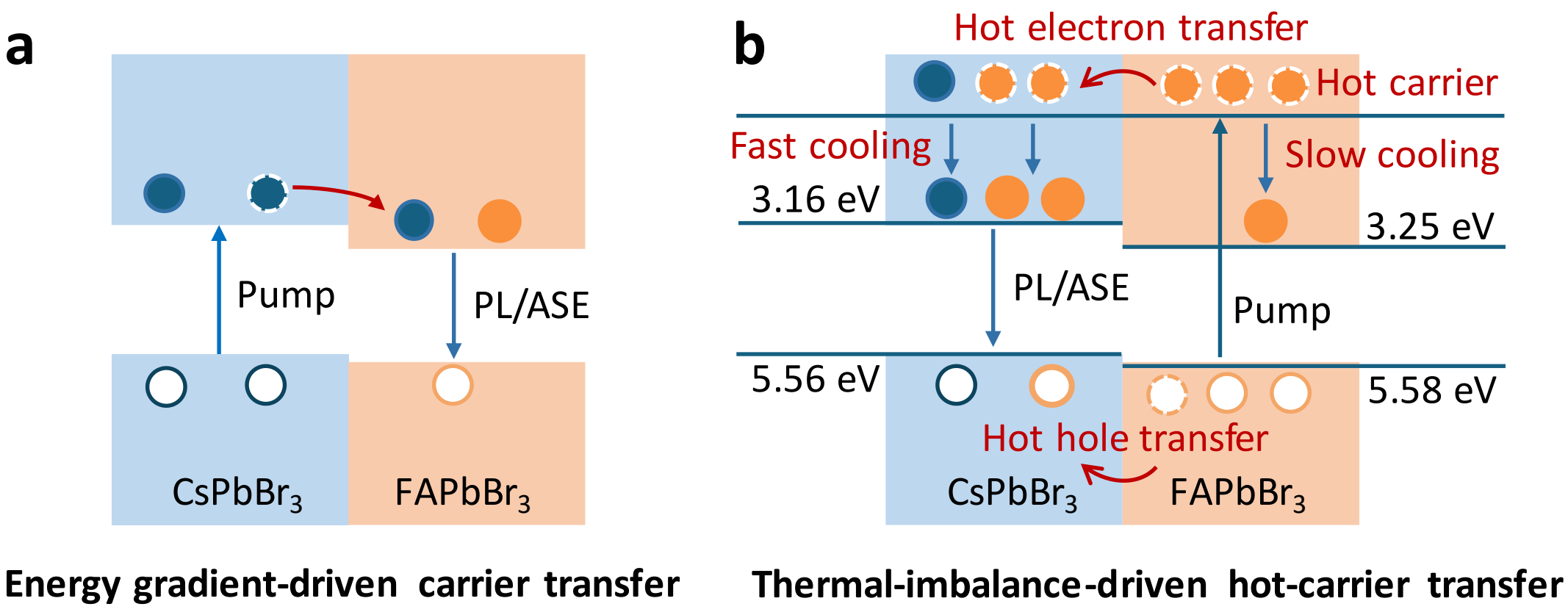

Figure 1. Carrier transfer mechanisms in semiconductor heterojunctions. a, Schematic illustration of conventional carrier transfer between high and low band gap semiconductors. Red arrows highlight the flow of cooled carriers across the interface driven by band energy gradient. **b**~~, c~~ Hot carrier transfer from a low band gap ($FAPbBr_3$) semiconductor to a high band gap semiconductor ($CsPbBr_3$). Under high-energy pumping conditions ~~(b)~~, hot carriers are generated in both high and low band gap semiconductors, while different hot carrier cooling rates influence the transfer dynamics, directing hot carriers into materials with faster cooling pathways. ~~With resonant pumping at low band gap semiconductor (c), hot carriers with excess energy above the band minimum (generated by scattering, Auger recombination or phase space filling) can move across the interface to the high band gap semiconductor.~~

We have ***added*** on the Page 10 of revised manuscript, that "We next extend our investigation to laminated heterojunctions to elucidate how interfacial carrier transfer processes influence hot carrier dynamics in such architectures. From UPS measurements, we know the energy barrier for electron transfer from $FAPbBr_3$ to $CsPbBr_3$ is 0.09 eV, while almost no barrier for hole transfer from $FAPbBr_3$ to $CsPbBr_3$ (Fig. 1b). Thus, for hot carriers generated under high-energy excitation (e.g., 400 nm, 3.10 eV), the excess energy (~0.77 eV above the $FAPbBr_3$ CBM) is sufficient to

overcome the 0.09 eV electron transfer barrier, enabling efficient directional hot-carrier transfer."

We have now ***performed*** a quantitative kinetic analysis of our TA data to extract the hot-carrier transfer time constant. The details are provided below and in the updated Supplementary Information.

We have ***added*** on Method part of revised manuscript, that "Room-temperature TA measurements were performed by commercial TA setup (Ultrafast Systems, HELIOS). We globally fitted the decay kinetic traces of $CsPbBr_3$ and $FAPbBr_3$ (data in Fig. 3c) to extract hot carrier transfer components ($CsPbBr_3$: hot carrier gain/bleach, $FAPbBr_3$: hot carrier transfer out). We use a comprehensive kinetic model that includes hot-carrier cooling, hot carrier transfer ($\tau_{transfer}$), and biexponential decay components (τ_{fast}, τ_{slow}) for both phases with corresponding branching ratios. The model also accounts for an instrument response function (IRF, deconvolved during fitting). All parameters were shared globally across both traces, and the fit was performed by least-squares minimization with regularization on the fast components. The fitted hot carrier transfer constant and carrier decay constant were listed in Table S2."

Table S2. Fitting parameters of the hot carrier transfer in $CsPbBr_3$/$FAPbBr_3$ laminated heterojunctions.

Phases	$\tau_{transfer}$	τ_{fast}	τ_{slow}
Laminated $FAPbBr_3$	**1.2 ± 0.7 ps**	258 ± 1 ps (Amplitude 0.14)	~984 ± 1 ps (Amplitude 0.86)
Laminated $CsPbBr_3$	**9.8 ± 0.6 ps**	6.3 ± 0.6 ps (Amplitude 0.94)	~4890 ± 5 ps (Amplitude 0.06)

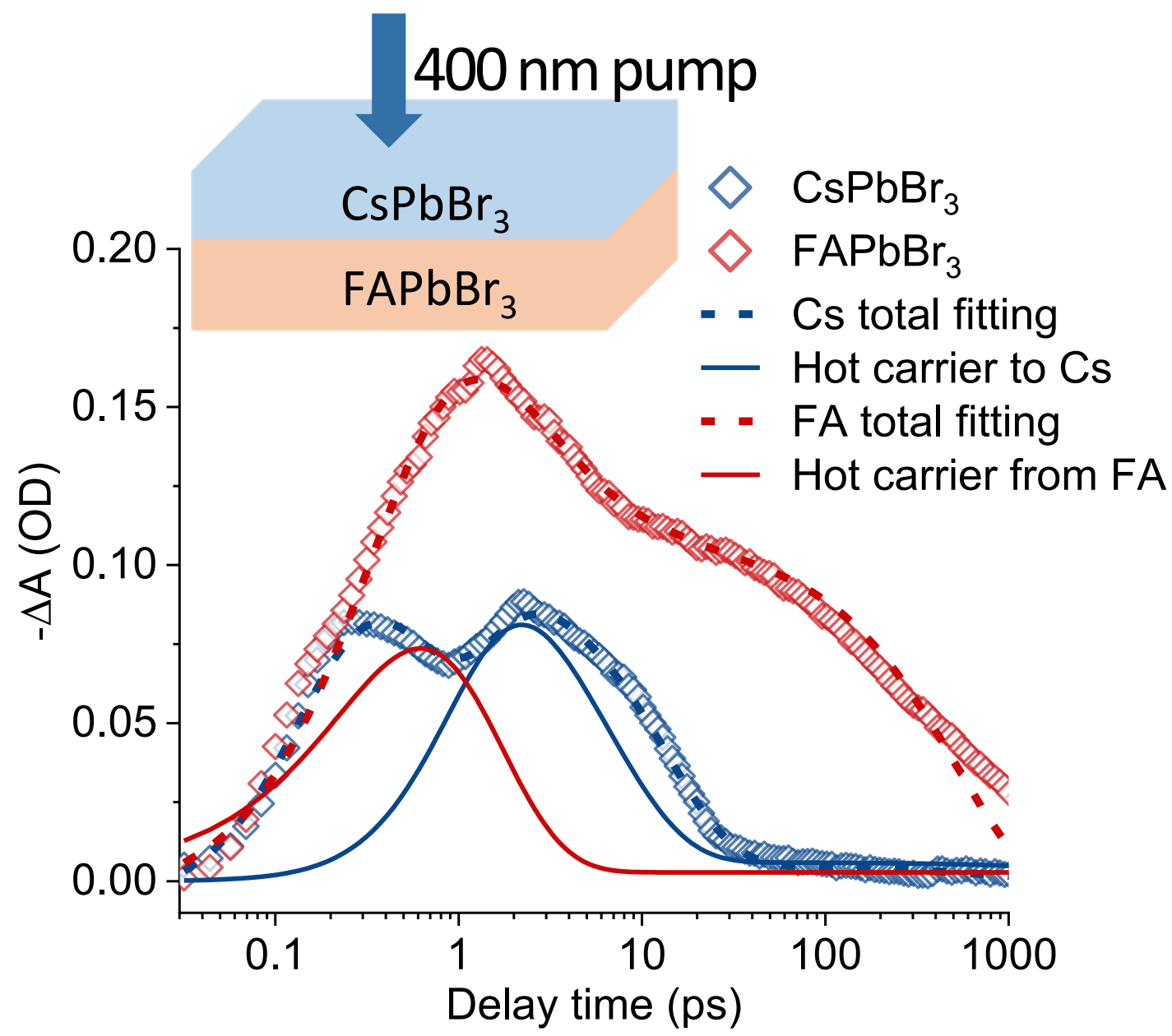

Fig. 3c, TA kinetics of laminated bilayer thin films. The pump laser (400 nm) was directed at $CsPbBr_3$ side. Global fitting was used to extract hot carrier transfer process in total TA signals (see details in Methods part). The dash line and solid line in (c) represent the fitting of total TA signals and the hot carrier transfer process, respectively.

We have ***added*** on the Page 10 of revised manuscript, that "As proposed in our earlier discussion of the hot carrier transfer mechanism (Fig. 1), when hot carriers are generated on both sides of the heterojunction, the differing cooling rates drive hot carriers toward the material with the faster cooling rate, such as $CsPbBr_3$. We globally fitted the TA kinetics at the $FAPbBr_3$ and $CsPbBr_3$ GSB positions using a sequential kinetic model to extract the hot-carrier transfer dynamics (see Methods for details). In Fig. 3c, the red and blue solid lines represent hot carriers transferred out of $FAPbBr_3$ and those injected into $CsPbBr_3$, respectively. Critically, the blue component constitutes the dominant fraction of the total carriers present in $CsPbBr_3$, confirming that hot-carrier transfer is the primary source of population in the $CsPbBr_3$. At early times, hot carriers initially accumulate in the $FAPbBr_3$ phase due to the hot-phonon bottleneck effect, reaching a maximum at ~0.6 ps, whereas hot carriers in $CsPbBr_3$ cool very rapidly (<0.2 ps). This population imbalance drives

directional hot-carrier transfer from $FAPbBr_3$ to $CsPbBr_3$, resulting in a pronounced hot-carrier population in $CsPbB_3$ that persists until ~2 ps and subsequently decays over approximately 10 ps. The fitted hot-carrier transfer time constant and carrier decay rates are summarized in Table S2. Notably, the hot-carrier population remains detectable in $CsPbBr_3$ for ~9.8 ps, which is considerably longer than the transfer out of $FAPbBr_3$ (~1.2 ps). This difference may be attributed to the finite transport time of hot carriers across the heterointerface."

We have ***added*** on the Page 11 of revised manuscript, that "After inserting the PMMA block layer, the GSB kinetics for both $CsPbBr_3$ and $FAPbBr_3$ (Fig. 3e, f) layers are close to those of the individual layers shown in Fig. 3a. These results further confirm that hot carrier transfer occurs within the laminated bilayers, and that the transfer is hindered when an intervening layer prevents direct interfacial interaction. Importantly, the hot-carrier transfer process persists for approximately 10 ps, which is fully consistent with our global fitting results (Fig. 3c)."

3. Defects play a critical role in determining the TA kinetics, optical gain, and laser performance of halide perovskite semiconductors. The nature of defects—whether shallow or deep—governs the relaxation pathways and rates of hot carriers. Specifically, hot carriers can be directly captured by trap states without first relaxing to the band edge (Nat. Commun. 11, 2712 (2020)). More critically, deep-level defects accelerate hot-carrier cooling by providing efficient energy dissipation channels, thereby weakening both the hot-phonon bottleneck and Auger reheating effects that otherwise slow energy loss (Nat. Commun. 15, 8120 (2024); Adv. Funct. Mater. 2025, e17023).

Optical gain requires population inversion, which depends on maximizing the number of excited carriers at the conduction band edge for stimulated emission. However, defect states—particularly deep-level traps—act as non-radiative recombination centers, diverting carriers from radiative processes and dissipating energy as heat. This parasitic pathway depletes the carrier population available for optical amplification, reducing the optical gain coefficient (Nat. Commun. 15, 5484 (2024)). Consequently, a higher excitation density is required to achieve population inversion, leading to an

increased laser threshold. Furthermore, trap states facilitate Auger recombination—a process that would otherwise require three-particle interactions—thereby shortening the gain lifetime under high-injection conditions. These defects also introduce energy barriers that increase optical waveguide losses (reducing the cavity quality factor), both of which impede efficient photon amplification (Nat. Commun. 15, 5484 (2024); Adv. Mater. 35, 2306102 (2023)).

In this work, the lamination process (120 °C, 100 MPa for 5 min) can markedly alter the defect density (Nat. Commun. 11, 5402 (2020)). Given that the fabricated hot-carrier laser devices achieve a threshold as low as 1.4 μJ/cm² under nanosecond pulsed excitation—five times lower than that of individual perovskite layers—a careful discussion of defect in the laminated samples is essential. Such analysis would ensure that the observed hot-carrier dynamics and performance enhancement is primarily attributed to directional hot-carrier transport rather than defect-mediated effects.

Our Response: We agree that a thorough discussion of defect contributions is essential to attribute the observed threshold reduction to hot-carrier transfer rather than to defect-mediated processes. We are grateful that this important point has been raised independently by multiple reviewers (Reviewer 2 comments 3). In recognition of this shared concern, we provide the same detailed response here for completeness.

In the revised manuscript, we have substantially expanded our defect analysis, incorporating both the literature cited by the reviewer and a comprehensive set of control experiments.

We further ***performed*** control experiments to rule out the possible contributions from defect states. Notably, we find that the PLQE values for the laminated structure does not decrease below the single layer values (revised Fig. S7), excluding an impact from potential defects at the interface to the light emission process.

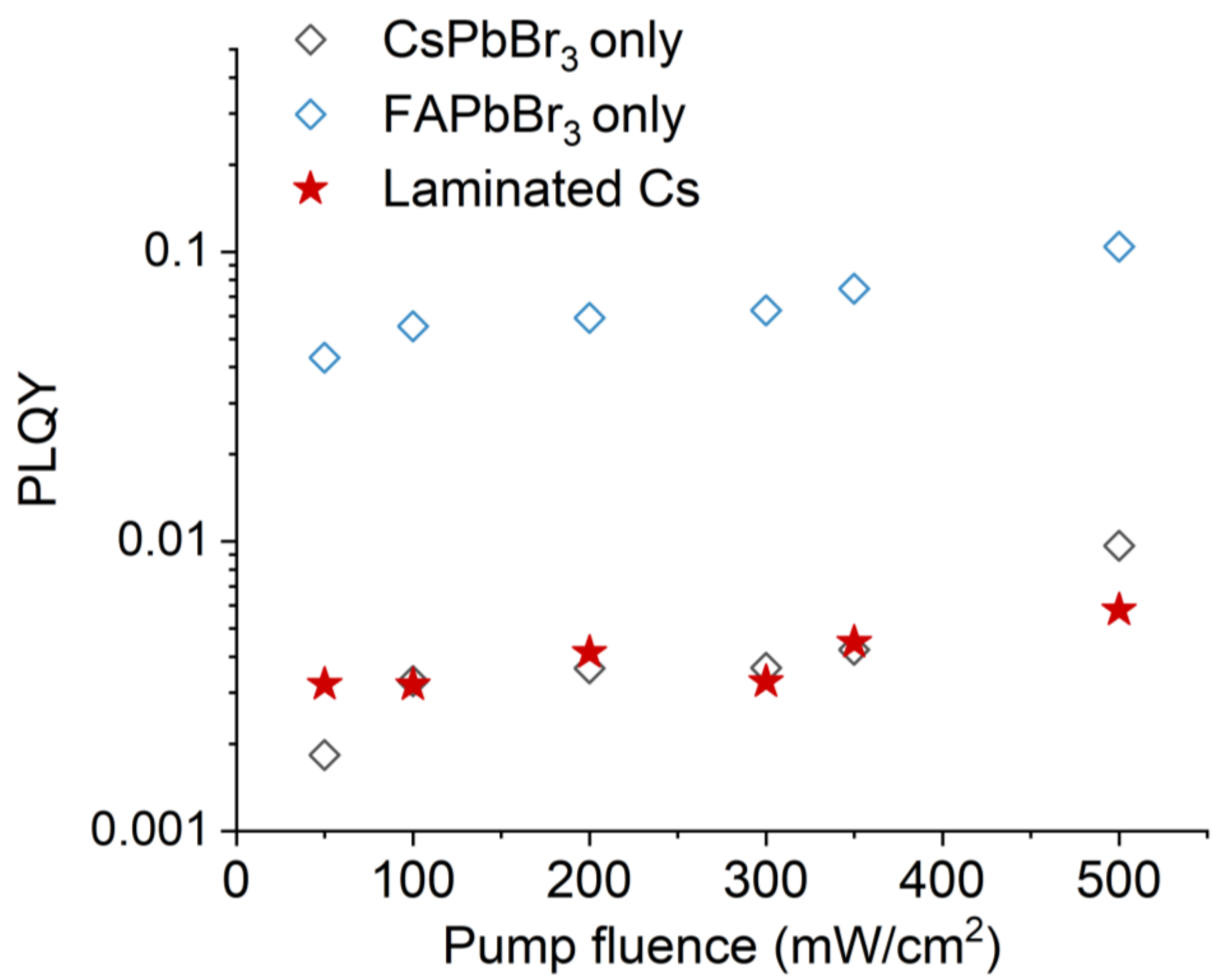

Figure S7. Comparison of photoluminescence quantum yield (PLQY) results before and after lamination process ~~$CsPbBr_3$ and $FAPbBr_3$ films~~. The samples were excited with a 400 nm continuous wave laser, and a ND filter wheel was used to control the excitation fluences. $FAPbBr_3$ films exhibit a PLQY that is at least ten times higher than that of $CsPbBr_3$ films at all pump powers. Importantly, after the lamination process, the PLQY of the $CsPbBr_3$ films remains almost unchanged compared to their pre-lamination values, further confirming that the defect density is not altered by lamination.

We have ***added*** the discussion on the Page 12 of revised manuscript, that "Recent studies have suggested that defect states could also form a three-level system, which might facilitate population inversion *(45-47)*. In our work, however, the heterojunction itself inherently provides a multi-level structure. The three-level laser picture we propose is grounded in the band alignment and hot carrier transfer in the $CsPbBr_3/FAPbBr_3$ heterojunction, not in the presence of defect states. Consequently, defects are not required to explain the observed population inversion or the reduction in lasing threshold. To further rule out possible

contributions from defect states, we compared the excited-state carrier dynamics before and after the lamination process. In both TA and time-resolved photoluminescence (TRPL) spectra, we observed blue-shifted signals on the high-energy tail at early times after lamination (Fig. S9 and S10), indicating the contribution of hot carriers transferred into the $CsPbBr_3$ side *(48-50)*. At later times (beyond 1 ns), both TA and TRPL spectra overlapped well on the low-energy tail, confirming that no additional trap states were introduced and that existing trap states were not strongly passivated by lamination.

Notably, under equivalent low carrier generation densities (Fig. S9c), the $CsPbBr_3$ ground-state bleach (GSB) signals before and after lamination exhibited similarly rapid decays over the first few picoseconds – a feature typically attributed to trap-mediated recombination *(51, 52)* – indicating comparable trap densities. Moreover, at both low and high carrier generation densities, the $CsPbBr_3$ GSB decay kinetics remained nearly identical before and after lamination, further demonstrating that the defect density is essentially unchanged by the lamination process. Consistently, after lamination, the $CsPbBr_3$ PL decay showed a prolonged lifetime during the initial 4 ns (Fig. 10c and f), which we attribute to fast hot-carrier transfer from the $FAPbBr_3$ layer. Beyond 4 ns, the decay kinetics before and after lamination became almost overlapped, confirming that the density of long-lived trap states remains unaffected *(53)*. Further, the PLQY of $CsPbBr_3$ films remained nearly unchanged after lamination compared to their pre-lamination values (Fig. S7). Taking together, all these results demonstrate that the lamination process does not significantly alter the defect density in $CsPbBr_3$. Therefore, the dominant mechanism responsible for reducing the ASE threshold in the laminated heterojunctions is hot-carrier transfer driven by cooling-rate gradients, not defect-mediated processes."

45. Wu, C. S. J., Wong, E. L., Li, H., Jiménez-López, J., Lin, C. K., Hsu, H. C., & Petrozza, A. Impact of Nano-Scale Defects on the Macroscopic Amplified Spontaneous Emission in Polycrystalline Perovskite Thin-Films. Advanced Materials, 38(24), e16903 (2026).

46. Zhang, L., Wang, Y., Chu, A. *et al.* Facet-selective growth of halide perovskite/2D semiconductor van der Waals heterostructures for improved optical gain and lasing. *Nat Commun* **15**, 5484 (2024).

47. S. Huang, Z. Shen, Y. Liao, Z. Liu, Z. Hu, Q. Li, Z. Zhang, S. Dong, J. Luo, J. Du, J. Tang, Y. Leng, Water-Resistant Subwavelength Perovskite Lasing from Transparent Silica-Based Nanocavity. Adv. Mater., 35, 2306102 (2023).

48. Righetto, M., Lim, S.S., Giovanni, D. *et al.* Hot carriers perspective on the nature of traps in perovskites. *Nat Commun* **11**, 2712 (2020).

49. Ye, J., Mondal, N., Carwithen, B.P. *et al.* Extending the defect tolerance of halide perovskite nanocrystals to hot carrier cooling dynamics. *Nat Commun* **15**, 8120 (2024).

50 L. Zhang, W. Xi, H. Zhang, et al. Efficient Hot Hole Extraction in Halide Perovskite/2D Semiconductor van der Waals Heterostructures. *Adv. Funct. Mater.*36, 22, e17023 (2026).

51. Fan, H., Li, F., Wang, P. *et al.* Methylamine-assisted growth of uniaxial-oriented perovskite thin films with millimeter-sized grains. *Nat Commun* **11**, 5402 (2020).

52. Wang, X., Jin, L., Sergeev, A., Liu, W., Gu, S., Li, N., Fan, K., Chen, S.C., Wong, K.S., Sun, X. and Zhao, N., Quasi-2D Dion-Jacobson phase perovskites as a promising material platform for stable and high-performance lasers. *Science Advances*, 9(43), eadj3476 (2023).

53. Fang, HH., Adjokatse, S., Shao, S. et al. Long-lived hot-carrier light emission and large blue shift in formamidinium tin triiodide perovskites. Nat Commun 9, 243 (2018).

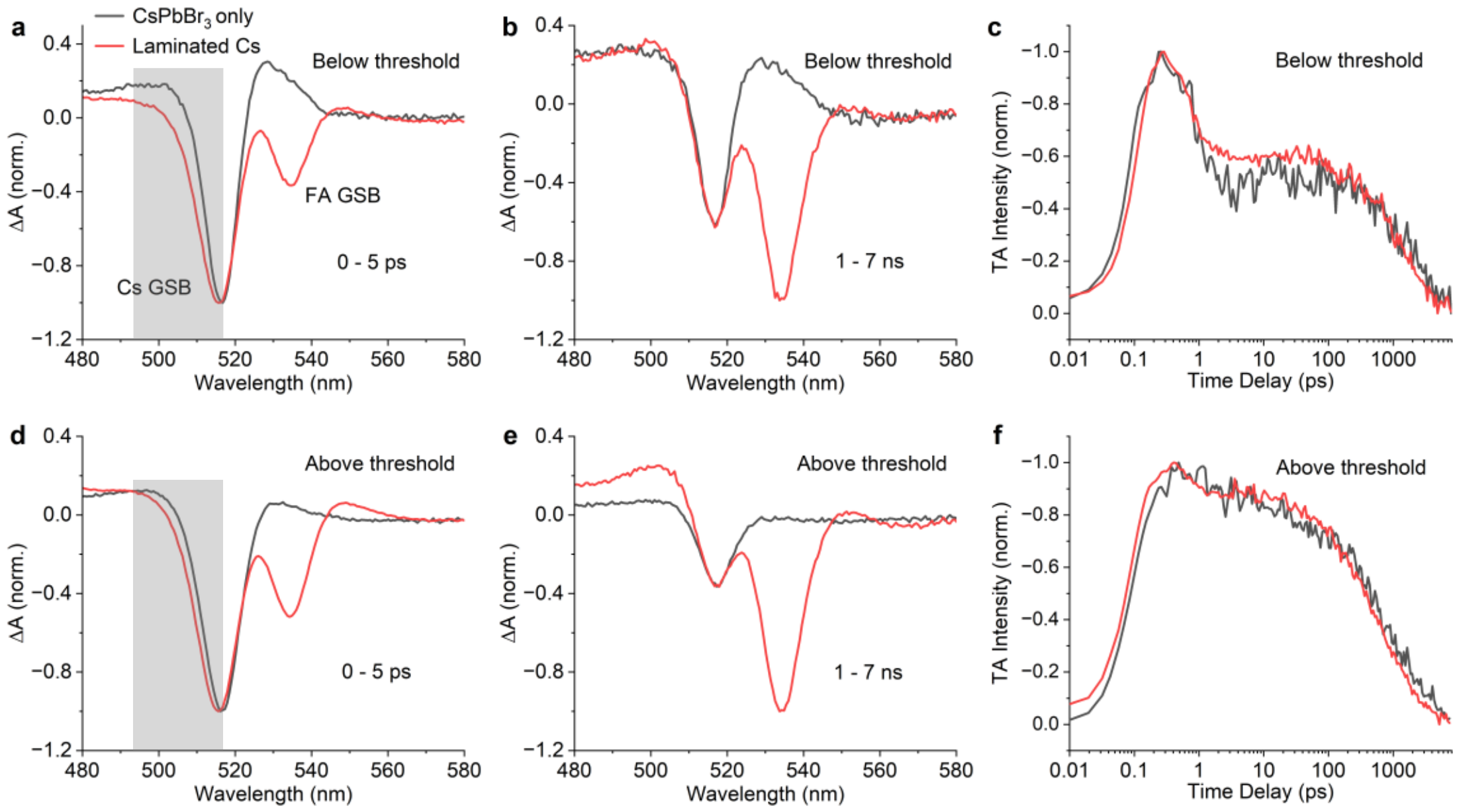

Figure S9. Comparison of transient absorption spectra before and after lamination process. a-c, Low carrier density generation regime (below ASE threshold), TA spectra (a, 0-5 ps; b, 1-7 ns) and kinetics of $CsPbBr_3$ GSB (c) before and after lamination. d-f, High carrier density generation regime (above ASE threshold), TA spectra (a, 0-5 ps; b, 1-7 ns) and kinetics of $CsPbBr_3$ (c) before and after lamination. Notably, at equivalent carrier generation densities, the $CsPbBr_3$ GSB decay kinetics exhibit nearly identical behavior before and after lamination, indicating that the defect density remains essentially unchanged by the lamination process.

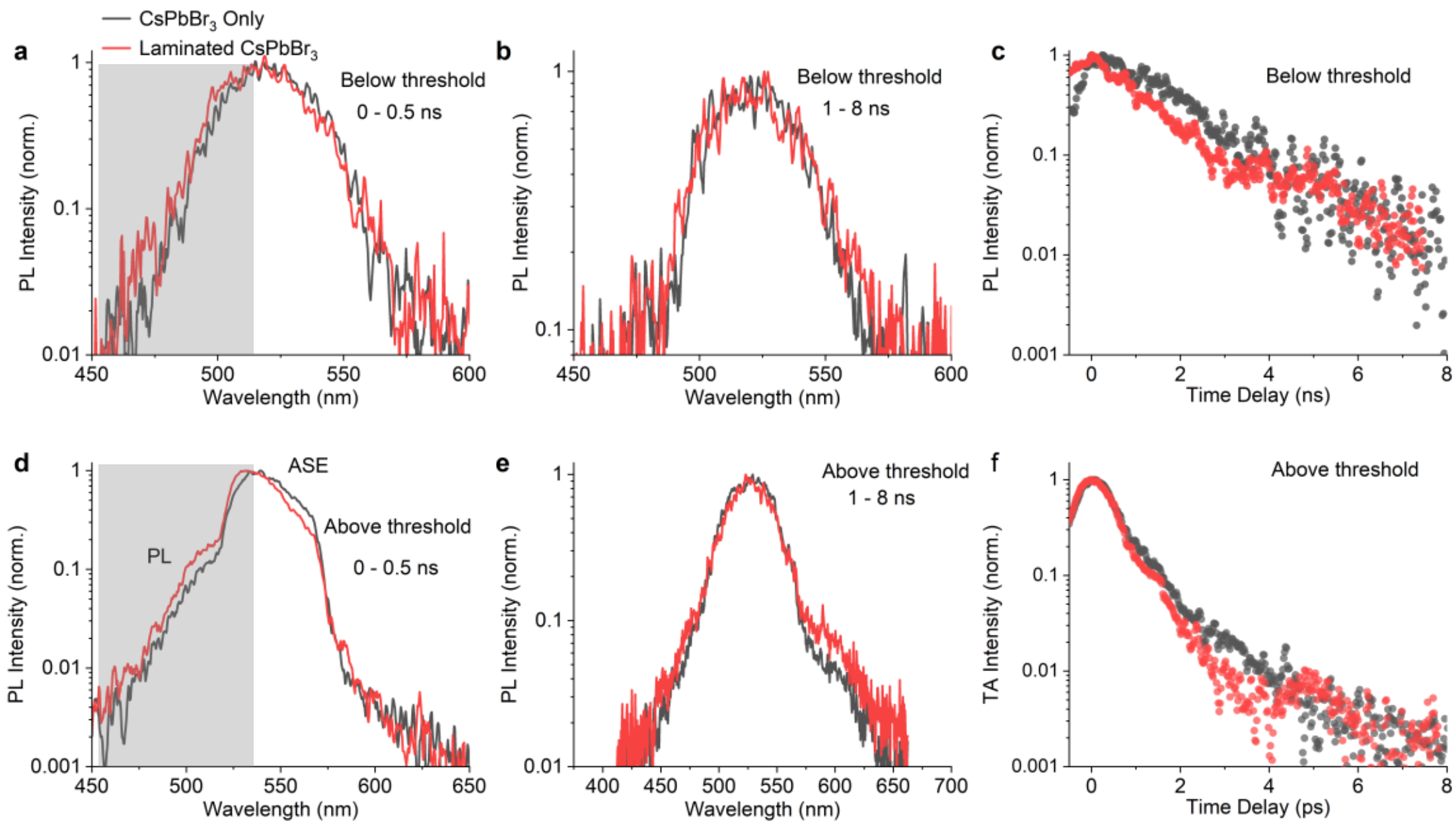

Figure S10. Comparison of time-resolved PL spectra before and after lamination process. a-c, With low excitation fluence (below ASE threshold), PL spectra (a, 0-5 ns; b, 1-8 ns) and decay kinetics of $CsPbBr_3$ (c) before and after lamination. d-f, With high excitation fluence (above ASE threshold), PL spectra (a, 0-5 ns; b, 1-8 ns) and decay kinetics of $CsPbBr_3$ (c) before and after lamination. Time resolved PL spectra were obtained by streak camera. After lamination, the $CsPbBr_3$ PL decay exhibits a prolonged lifetime during the initial 4 ns, which we attribute to fast hot-carrier transfer from the $FAPbBr_3$ layer. Beyond 4 ns, the decay kinetics before and after lamination become nearly overlapped, confirming that the lamination process does not alter the density of long-lived trap states.

We further **compared** the ASE thresholds of laminated $CsPbBr_3/FAPbBr_3$ heterojunction and a control sample with a PMMA interlayer inserted between the two perovskite layers. Both samples underwent the identical lamination process; however, the PMMA layer blocks hot-carrier transfer, thereby disabling the transfer pathway.

We have ***plotted*** below and in revised manuscript Fig. S12 all ASE threshold results with error bars indicated.

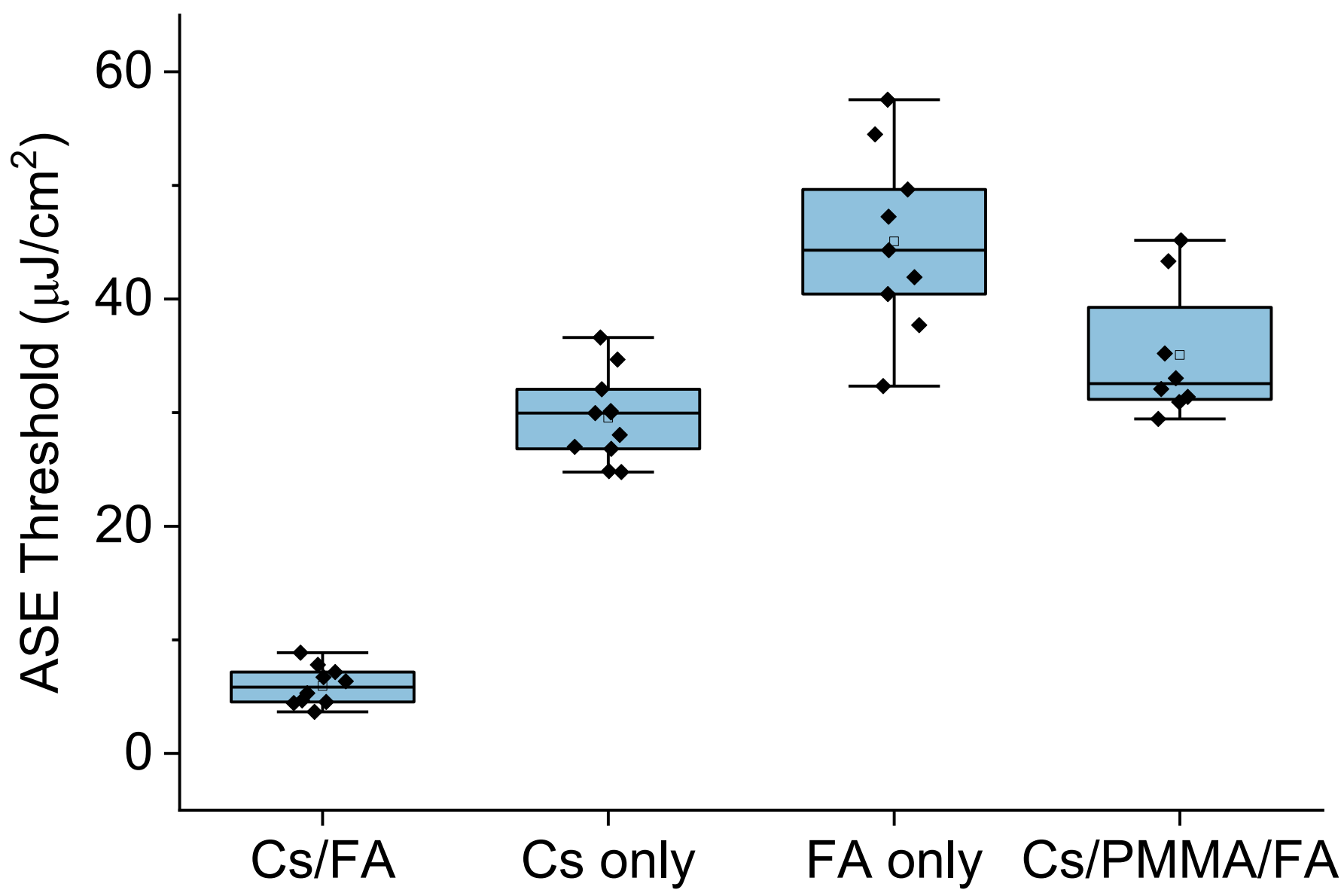

Figure S12. Comparison of amplified spontaneous emission (ASE) thresholds. ASE thresholds were measured for the laminated $CsPbBr_3/FAPbBr_3$ heterojunction,

single-layer $CsPbBr_3$, single-layer $FAPbBr_3$, and a control sample with a PMMA interlayer inserted between the two perovskite layers during the lamination process. Each condition was measured on at least five devices (individual data points shown as solid squares). A 355 nm nanosecond pulsed laser was used for excitation.

We have ***added*** on the Page 15 of revised manuscript, that "Using 355 nm nanosecond pulsed excitation (1 ns pulse width, 1 kHz repetition rate) laminated $CsPbBr_3$ samples exhibit a substantially lower ASE threshold (~4 μJ/cm²) compared to individual samples (Fig. 4a). Statistical analysis across multiple devices confirms the reproducibility of this threshold reduction (Fig. S12). We further compared the ASE thresholds of laminated CsPbBr3/FAPbBr3 heterojunction and a control sample with a PMMA interlayer inserted between the two perovskite layers. Both samples underwent the identical lamination process; however, the PMMA layer blocks hot-carrier transfer, thereby disabling the hot transfer pathway. Consequently, the control sample exhibited no ASE threshold reduction relative to single-layer $CsPbBr_3$ (Fig. S12), confirming that the threshold reduction in the laminated heterojunction is primarily attributable to hot-carrier transfer."

4. Given that steady-state absorption, transient absorption, and laser performance measurements are critically dependent on the thickness of the perovskite photoactive layer, because film thickness significantly affects carrier transport and optical confinement effects. a detailed specification of the sample thickness is essential. Such information is crucial for ensuring the validity and comparability of the experimental results.

Our Response: We thank the reviewer raises this valid point.

We have ***added*** in the Method part of revised manuscript, that "Field emission top-view and cross-section SEM images of perovskite films were taken with a ZEISS Supra60 VP scanning electron microscope. SEM cross-sections show thicknesses of ~90 nm for single-layer $CsPbBr_3$, ~200 nm for single-layer $FAPbBr_3$, and ~250 nm for the laminated bilayer.".

5. The proposition that hot carrier transfer constructs an ideal three-level system in the heterojunction requires rigorous quantitative support. While this concept is central to the work, the current manuscript lacks essential quantitative data to substantiate it. Specifically, it does not delineate the energy level spacing of the proposed three-level system, nor does it determine the critical carrier density necessary for achieving population inversion. Furthermore, the optical gain coefficient—a key parameter linking the three-level system to laser performance—has not been extracted from the experimental data. To address this, it is recommended that the authors provide a detailed energy level diagram with labeled energy values and calculate the optical gain using the PL or ASE or TA results. Such an analysis would quantitatively establish the contribution of the three-level system to the observed reduction in laser threshold.

Our Response: We thank the reviewer for these suggestions.

We have **modified** the scheme in Fig 1 to quantitatively show the energy level diagram between $CsPbBr_3$ and $FAPbBr_3$ CBM and VBM.

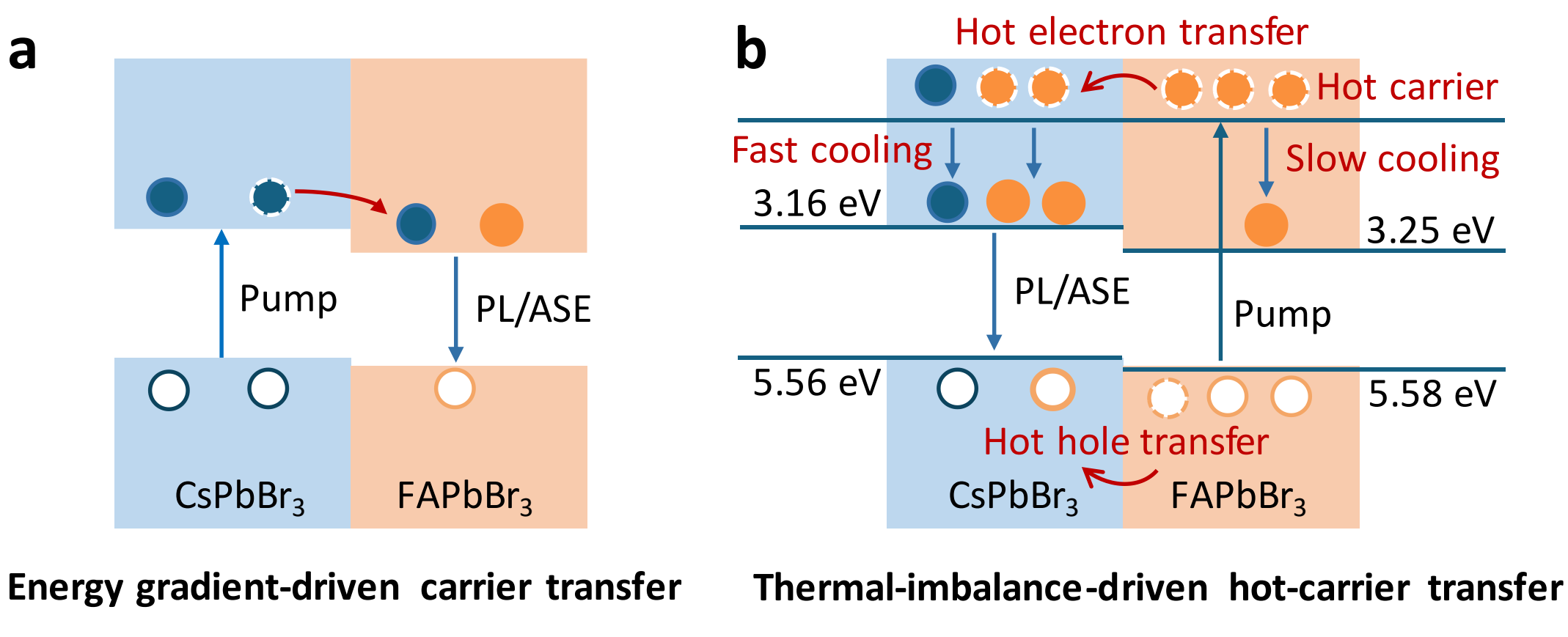

Figure 1. Carrier transfer mechanisms in semiconductor heterojunctions. a, Schematic illustration of conventional carrier transfer between high and low band gap semiconductors. Red arrows highlight the flow of cooled carriers across the interface driven by band energy gradient. **b**~~, c~~ Hot carrier transfer from a low band

gap ($FAPbBr_3$) semiconductor to a high band gap semiconductor ($CsPbBr_3$). Under high-energy pumping conditions (b), hot carriers are generated in both high and low band gap semiconductors, while different hot carrier cooling rates influence the transfer dynamics, directing hot carriers into materials with faster cooling pathways. With resonant pumping at low band gap semiconductor (c), hot carriers with excess energy above the band minimum (generated by scattering, Auger recombination or phase space filling) can move across the interface to the high band gap semiconductor.

We have ***added*** on the Page 10 of revised manuscript, that "We next extend our investigation to laminated heterojunctions to elucidate how interfacial carrier transfer processes influence hot carrier dynamics in such architectures. From UPS measurements, we know the energy barrier for electron transfer from $FAPbBr_3$ to $CsPbBr_3$ is 0.09 eV, while almost no barrier for hole transfer from $FAPbBr_3$ to $CsPbBr_3$ (Fig. 1b). Thus, for hot carriers generated under high-energy excitation (e.g., 400 nm, 3.10 eV), the excess energy (~0.77 eV above the $FAPbBr_3$ CBM) is sufficient to overcome the 0.09 eV electron transfer barrier, enabling efficient directional hot-carrier transfer."

We ***added*** on the Page 11 of revised manuscript, that "Based on the hot carrier transfer mechanism discussed above, the planar $FAPbBr_3$/$CsPbBr_3$ heterojunctions are expected to mimic an ideal three-level system,. High-energy photoexcitation generates hot carriers in $FAPbBr_3$ (level 3), which exhibits a relatively slow cooling rate. These hot carriers are then rapidly funneled into the $CsPbBr_3$ side within a few picoseconds, and subsequently cool to the band-edge excited state of $CsPbBr_3$ (level 2) on a sub-picosecond timescale. Finally, the carriers radiatively relax to the $CsPbBr_3$ ground state (level 1), emitting light (Fig. 1b). Such a three-level configuration would leading to an enhancement of optical gain in $CsPbBr_3$."

We next extend our investigation to laminated heterojunctions to elucidate how interfacial carrier transfer processes influence hot carrier dynamics in such architectures. From UPS measurements, we know the energy barrier for electron

transfer from $FAPbBr_3$ to $CsPbBr_3$ is 0.09 eV, while almost no barrier for hole transfer from $FAPbBr_3$ to $CsPbBr_3$ (Fig. 1b). Thus, for hot carriers generated under high-energy excitation (e.g., 400 nm, 3.10 eV), the excess energy (~0.77 eV above the $FAPbBr_3$ CBM) is sufficient to overcome the 0.09 eV electron transfer barrier, enabling efficient directional hot-carrier transfer."

In the revised manuscript, we have now ***extracted*** the net modal gain coefficient directly from our transient absorption, steady-state absorption, and fluence-dependent PL data using the well-established relationship between the ground-state bleach (GSB) amplitude and the carrier density at threshold.

We have ***added*** on the Method part of revised manuscript, that "The optical gain coefficient *g* is a core parameter for evaluating the performance of gain media, which quantifies the increase in light intensity per unit length as light waves propagate in the medium. Variable strip length (VSL) or variable pump intensity (VPI) methods are commonly used to extract the optical gain coefficient *(1-2)*. Here, we quantitatively extracted the net optical gain coefficients directly from our transient absorption data using the well-established relationship:

$$g_{th} = \frac{10^{-(A_0+\Delta A)} - 1}{d}$$

where d is the thickness of the film, A_0 is the steady state absorbance, ΔA is the TA signals at the lasing threshold. The threshold carrier density (N_{th}) was determined from the fluence-dependent PL data. The calculated threshold carrier density and optical gain coefficient values were listed in Table. S1.

1. S. Milanese, M. L. De Giorgi, M. Anni, M. I. Bodnarchuk, L. Cerdán, Rationalizing the Amplified Spontaneous Emission Mechanism in CsPbBr3 Perovskite Nanocrystals Films by means of Optical Gain Measurements. Adv. Optical Mater., 12, 2401078 (2024).
2. Cerdán, L. Simultaneous retrieval of optical gains, losses, and threshold in active waveguides. Optics & Laser Technology, 121, 105814 (2020).

Table S1. Optical gain coefficient values for $CsPbBr_3$ films before and after lamination process.

Samples	Threshold carrier density N_{th} (cm^{-3})	Optical gain coefficient g_{th} (cm^{-1})
$CsPbBr_3$ Only	~2.6×10^{18}	**~255**
Laminated $CsPbBr_3/FAPbBr_3$ heterojunction	~7.3×10^{17}	**~712**

We ***added*** on the Page 11 of revised manuscript, that "These findings further confirm the occurrence of hot carrier transfer from $FAPbBr_3$ to $CsPbBr_3$ within the laminated structures, which markedly enhances the carrier density at $CsPbBr_3$ excited states and facilitates their population inversion and optical gain, thereby contributing to the observed large reduction in ASE threshold. We further extracted the net optical gain coefficients from our transient absorption data (see Methods and Table S1), which is a core parameter for evaluating the performance of gain media. The result clearly show that the laminated heterojunction achieves a higher net gain coefficient (~712 cm^{-1}) despite requiring a much lower threshold carrier density (7.3×10^{17} cm^{-3}) compared to pure $CsPbBr_3$ (~255 cm^{-1}, 2.6×10^{18} cm^{-3}). These results quantitatively confirm that hot-carrier transfer creates a more efficient multi-level system, enabling population inversion at lower carrier density with higher optical gain."

6. The comparison of laser thresholds with existing perovskite devices is insufficiently comprehensive. While the paper reports that the fabricated VCSEL exhibits a threshold of 1.4 μJ/cm²—five times lower than that of a single perovskite layer—this benchmark is limited to an internal reference. A broader contextualization is needed against state-of-the-art values reported for other perovskite VCSELs and planar heterojunction lasers. Moreover, the work would benefit from a critical analysis of its advantages and limitations relative to other material systems, such as colloidal

nanocrystal and quasi-2D perovskite lasers. Therefore, it is recommended that the authors include an up-to-date comparison table of threshold values from representative perovskite laser devices. This addition would effectively highlight the progress and innovation of the present work within the field.

Our Response: We thank the reviewer for this important suggestion. In the revised manuscript, we have now compiled a comprehensive comparison of lasing thresholds for representative perovskite laser devices reported in the literature. The table below summarizes state-of-the-art values across different material systems and resonator configurations, with a focus on nanosecond optically pumped devices for fair comparison.

We ***added*** on the Page 16 of revised manuscript, that "Importantly, the lasing threshold of the laminated heterojunctions (~1.4 μJ/cm^2) is considerably lower than that of pure $CsPbBr_3$ (~5 μJ/cm^2) and $FAPbBr_3$ VCSELs (~4 μJ/cm^2, Fig. S15), consistent with the observed trends in ASE thresholds. With a lasing threshold of 1.4 μJ/cm^2 under nanosecond pumping, our laminated heterojunction VCSEL achieves one of the lowest values reported for nanosecond-pumped perovskite VCSELs (Table S3, Supporting Information). However, continuous-wave operation, essential for practical applications, remains to be demonstrated. This limitation may be addressed by future work on thermal management or low-temperature operation."

Table S3. Summary of the recent reported state-of-the-art lasers

Materials	Cavity	Pump (nm)	Emission (nm)	Pulse	Threshold	T (K)	Reference
$MAPbI_3$ film	DFB	445	790	20 ns	5 KW cm^{-2}	160	*Nano Lett.* 16, 7, 4624 (2026)

$CsPbBr_3$ QD film	VCSEL	400	505	5 ns	900 μJ cm^{-2}	RT	*Adv. Funct. Mater.* 27, 1605088 (2017)
$CsPbBr_3$ QD film	VCSEL	355	523	5 ns	98 μJ cm^{-2}	RT	*ACS Photonics* 4, 2281 (2017)
$CsPbBr_3$ film	DFB	355	539	0.3 ns	7.2 μJ cm^{-2}	RT	*Adv. Mater.* 31, 1903717 (2019)
N2F8/P2F8 quasi 2D film	DFB	488<br>337	555/553<br>559/52	CW<br>3 ns	0.059/0.045 KW cm^{-2}<br>4.7/32.8 μJ cm^{-2}	RT	*Nature* 585, 53 (2020)
$CsPbBr_3$ microcrystals	plasmonic lasers	480	540	4 ns	0.2 mJ cm^{-2}	RT	*Sci. Adv.*, 7, abf3362 (2021)
Quasi-2D/3D heterostructure	VCSEL	355	538	1.1 ns	46 μJ cm^{-2}	RT	*Adv. Funct. Mater.*, 32, 2200772 (2022)
Dion–Jacobson quasi 2D film	DFB	532	532-535	0.5 ns	5.5/8.9 μJ cm^{-2}	RT	*Sci. Adv.*, 9, eadj3476 (2023)
$MAPbI_3$ film	DFB	532	784	3 ns	100 mJ cm^{-2}	RT	*ACS Photonics*, 10, 1591 (2023)
$NMA_2FA_8Pb_8Br_{26}$ quasi-2D film	DFB	337	537	0.8 ns	1 μJ cm^{-2}	RT	*Adv. Funct. Mater.*, 33, 2301794 (2023)
Quasi-2D double heterostructure film	VCSEL	355	531	8 ns	53.7 μJ cm^{-2}	RT	*Nano Lett.*, 24, 6010 (2024)
$(FAPbI_3)_{0.95}$ $(MAPbBr_3)_{0.05}$ film	DFB	532	787–830	2.3 ns	130 μJ cm^{-2}	RT	*Adv. Opt. Mater.*, 12, 2302496 (2024)
$Cs_{0.1}FA_{0.9}PbI_{2.855}Br_{0.145}$ LED	ASE	447	790	1000 ns	3.8 KW cm^{-2}	70	*Nat. Photon., 18, 132 (2024)*
$FA_{0.1}MA_{0.9}PbBr_3$ single-crystals	VCSEL	405	551	CW	0.4 W cm^{-2}	RT	*Sci. Adv.*, 11, eadr8826 (2025).
$CsPbBr_3$ nanocrystals	Fabry-Pérot cavity	355	530	7 ns	155 mJ cm^{-2}	RT	*Sci. Adv.*, 11, eadq9002(2025).
$CsPb(Cl_{1-x}Br_x)_3$	In-glass	405	511	CW	57.2 mW cm^{-2}	83	*Sci. Adv.*, 12,

nanocrystals	structures						eadz8460 (2026).
$CsPbBr_3/FAPbBr_3$ film	VCSEL	355	553	1 ns	1.4 μJ cm^{-2}	RT	**This work**

7. Light is fundamentally a wave characterized by both amplitude and phase. Laser light, by definition, is coherent—meaning it exhibits well-defined phase relationships in both time and space (Svelto, O. Principles of Lasers 475-504 (Springer US, 2010).; Nat. Photonics 3, 546–549 (2009).). To conclusively demonstrate that a sharp emission peak originates from lasing rather than amplified spontaneous emission (ASE) or other incoherent processes, evidence beyond intensity-dependent measurements is required. While the current work shows how the peak amplitude evolves with pump fluence—a necessary condition for identifying a lasing threshold—it does not constitute sufficient proof. The critical missing piece is phase information. Therefore, it is recommended that the authors provide direct evidence of coherence, such as measurements of temporal coherence (e.g., via interferometry to determine coherence length) or spatial coherence (e.g., via Young's double-slit experiment). Such data would unambiguously confirm the coherent nature of the emission and solidify the claim of achieving lasing.

Our Response: We thank the reviewer for this rigorous and important comment. We fully agree that intensity-dependent measurements alone, while necessary, are insufficient to definitively prove lasing. The reviewer is correct that direct evidence of coherence — both temporal and spatial — is required to unambiguously distinguish lasing from amplified spontaneous emission (ASE) or other incoherent processes.

To address this essential requirement, we have ***performed*** Michelson interferometry to directly measure the temporal coherence of the emission from our laminated heterojunction VCSEL. The results, presented below and in the revised manuscript (new Supplementary Fig. S12), provide conclusive evidence of laser operation.

We ***added*** on the Page 16 of revised manuscript, that "Further tuning of the laser emission was achieved by adjusting the MgF_2 spacer thickness within the cavity, resulting in color-tunable laser output from 545 to 565 nm (Fig. 4d). The laser nature of the emission is conclusively confirmed by direct coherence measurements. Michelson interferometer reveals a coherence time of ~2.7 ps above threshold (Fig. S14). Together with super-linear power dependence, spectral narrowing to 0.2 nm, thickness dependent lasing output, and a well-collimated beam with a converged angular distribution, these data unambiguously demonstrate that the laminated heterojunction VCSEL operates as a true laser *(54, 53)*."

54. Samuel, I., Namdas, E. & Turnbull, G. How to recognize lasing. *Nature Photon* 3, 546–549 (2009).

55. J.Song, Q.Shang, X.Deng, Y.Liang, C.Li, X.Liu, Q.Xiong, Q.Zhang, Continuous-Wave Pumped Perovskite Lasers with Device Area Below 1 μm2. *Adv. Mater.*, 35, 2302170 (2023).

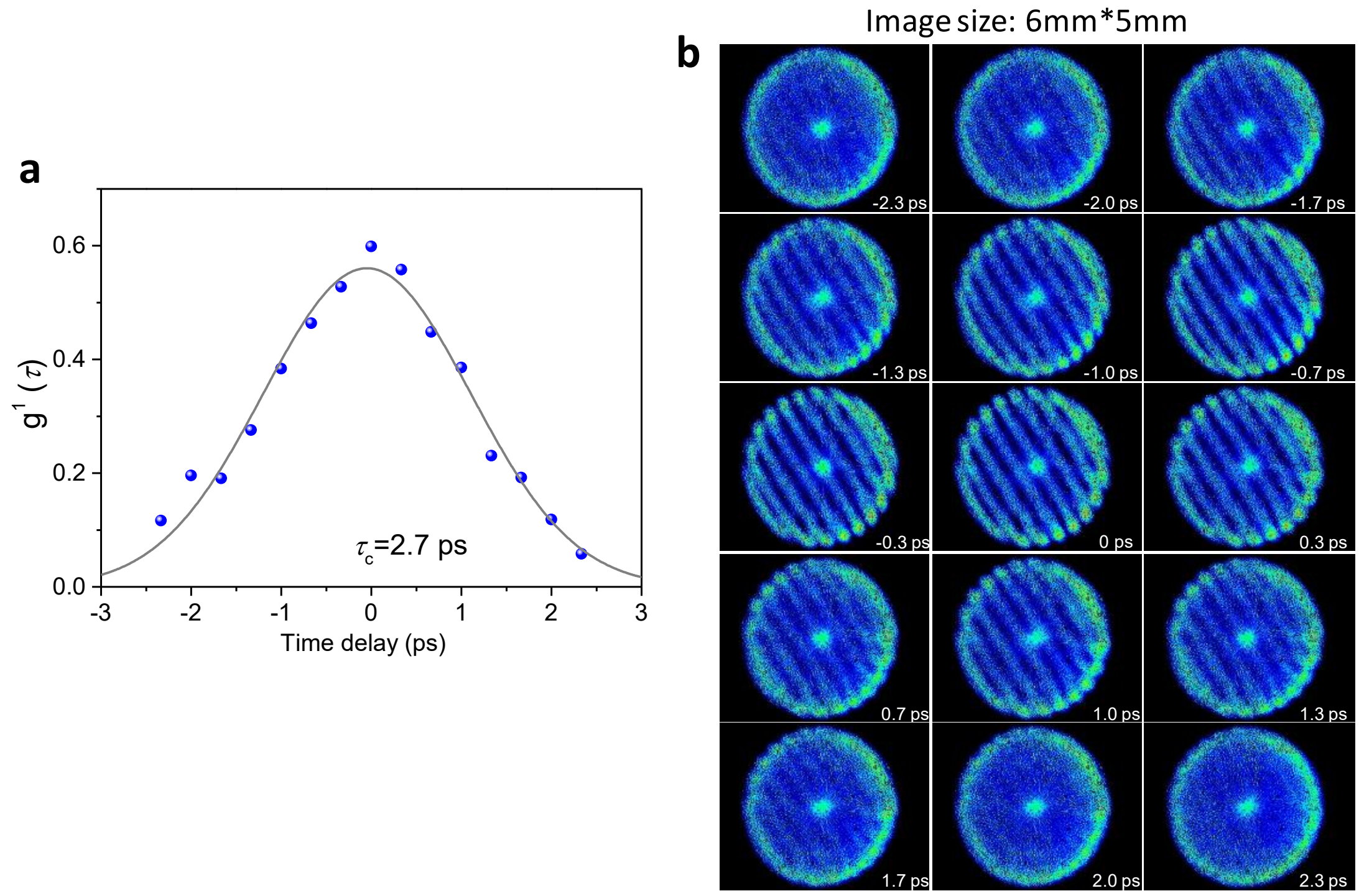

Figure S14. Temporal coherence of the laser emission from the laminated heterojunction VCSEL. a, First-order coherence function $g^1(\tau)$ measured above the

lasing threshold using a Michelson interferometer. The data points are obtained from the envelope of interference fringes as a function of time delay. The solid line represents a Gaussian fit to the coherence envelope, yielding a coherence time of 2.7 ps. The solid line represents a Gaussian fit to the data. b, Representative Michelson interference pattern at time delay from -2.3 ps to 2.3 ps. The high-contrast fringes directly demonstrate the temporal coherence of the laser emission.

Reviewer #4 (Remarks to the Author):

This manuscript reports laminated CsPbBr3/FAPbBr3 bilayer heterojunctions and proposes that a dynamic population gradient, arising from different hot-carrier cooling pathways, drives ultrafast directional hot-carrier transfer from FAPbBr3 to CsPbBr3. The authors further claim that this process increases the carrier population in CsPbBr3 and thereby reduces the ASE and lasing thresholds. **The idea is interesting and potentially important. In particular, using interfacial hot-carrier dynamics to improve optical gain is an appealing concept.**

Our Response: We thank the reviewer for recognizing the potential significance of our work.

However, in my view, the current experimental evidence is still insufficient to support the central mechanistic claims of the paper. Several key observations remain open to alternative interpretations. As a result, I do not believe the manuscript, in its present form, meets the standard for publication in Nature Communications. Below are several issues

Our Response: We take this concern seriously. Below we address each specific issue raised.

1. The authors argue that hot-carrier transfer from FAPbBr3 to CsPbBr3 increases the carrier density in CsPbBr3 and promotes population inversion. However, for lasing or ASE, the relevant population is the band-edge carrier population rather than the

transient hot-carrier population. Under the type-II alignment proposed in the manuscript, band-edge electrons in CsPbBr3 should energetically favor transfer to FAPbBr3, which would deplete, rather than enhance, the steady-state band-edge population in CsPbBr3. The manuscript does not sufficiently explain how the proposed hot-carrier injection competes with the energetically favorable band-edge charge separation. A quantitative discussion of these competing channels is essential. Without such an analysis, the link between ultrafast hot-carrier transfer and lower lasing threshold remains speculative.

Our Response: We thank the reviewer for this rigorous analysis. We fully agree that a quantitative discussion of the competing timescales is essential.

We ***added*** on the Page 11 of revised manuscript, that "Although $CsPbBr_3$ has a high CBM compared to that of $FAPbBr_3$, we did not observe the conventional carrier transfer from $CsPbBr_3$ to $FAPbBr_3$~~, likely due to fast carrier recombination in $CsPbBr_3$ (<100 ps) before carrier reaching interface.~~ Global fitting of the hot-carrier transfer dynamics reveals that both carrier cooling and hot-carrier injection into $CsPbBr_3$ are extremely fast (the transfer time constant is ~9.8 ps, while initial cooling occurs in <0.2 ps in $CsPbBr_3$). This enables efficient carrier accumulation in the $CsPbBr_3$ phase before any significant decay can take place. The TA decay kinetics further show that the carrier lifetime in $CsPbBr_3$ is only tens of ps (Table S2), which is much shorter than the hundreds of picoseconds observed in $FAPbBr_3$. Moreover, most conventional carrier transport across the bilayer heterostructure is reported to occur on timescales longer than 100 ps *(39-41)*. Any carriers that reach the conduction band edge of $CsPbBr_3$ recombine radiatively to contribute to optical gain before they can transfer back to $FAPbBr_3$."

39. Fu, Y., Zheng, W., Wang, X., Hautzinger, M.P., Pan, D., Dang, L., Wright, J.C., Pan, A. and Jin, S., Multicolor heterostructures of two-dimensional layered halide perovskites that show interlayer energy transfer. *Journal of the American Chemical Society*, 140(46), 15675-15683 (2018).

40. Li, W., Hu, L., Ma, J., Jiang, C., Zhang, S., Chen, Y., Hu, J., Liu, X., Wu, T. and Li,

D., Enhancing self-trapped exciton emission via energy transfer in two-dimensional/quantum dot perovskite heterostructures. *ACS Photonics*, 9(6), 2008-2014, (2022).

41. C. Guo, J. Guo, W. Song, S. Peng, M. Sun, G. Xing, L. Yu, Atomic heterointerface engineering and nonequilibrium carrier dynamics for enhanced photocatalysis in halide perovskite/MoS_2 systems. *Angew. Chem. Int. Ed.*, 64, e202506436 (2025).

We have now ***performed*** a quantitative kinetic analysis of our TA data to extract the hot-carrier transfer kinetics to confirm that hot-carrier transfer is the primary source of population in the $CsPbBr_3$. The details are provided below and in the updated Supplementary Information.

We have ***added*** on Method part of revised manuscript, that "Room-temperature TA measurements were performed by commercial TA setup (Ultrafast Systems, HELIOS). We globally fitted the decay kinetic traces of $CsPbBr_3$ and $FAPbBr_3$ (data in Fig. 3c) to extract hot carrier transfer components ($CsPbBr_3$: hot carrier gain/bleach, $FAPbBr_3$: hot carrier transfer out). We use a comprehensive kinetic model that includes hot-carrier cooling, hot carrier transfer ($\tau_{transfer}$), and biexponential decay components (τ_{fast}, τ_{slow}) for both phases with corresponding branching ratios. The model also accounts for an instrument response function (IRF, deconvolved during fitting). All parameters were shared globally across both traces, and the fit was performed by least-squares minimization with regularization on the fast components. The fitted hot carrier transfer constant and carrier decay constant were listed in Table S2."

Table S2. Fitting parameters of the hot carrier transfer in $CsPbBr_3$/$FAPbBr_3$ laminated heterojunctions.

Phases	$\tau_{transfer}$	τ_{fast}	τ_{slow}

Laminated $FAPbBr_3$	**1.2 ± 0.7 ps**	258 ± 1 ps (Amplitude 0.14)	~984 ± 1 ps (Amplitude 0.86)
Laminated $CsPbBr_3$	**9.8 ± 0.6 ps**	6.3 ± 0.6 ps (Amplitude 0.94)	~4890 ± 5 ps (Amplitude 0.06)

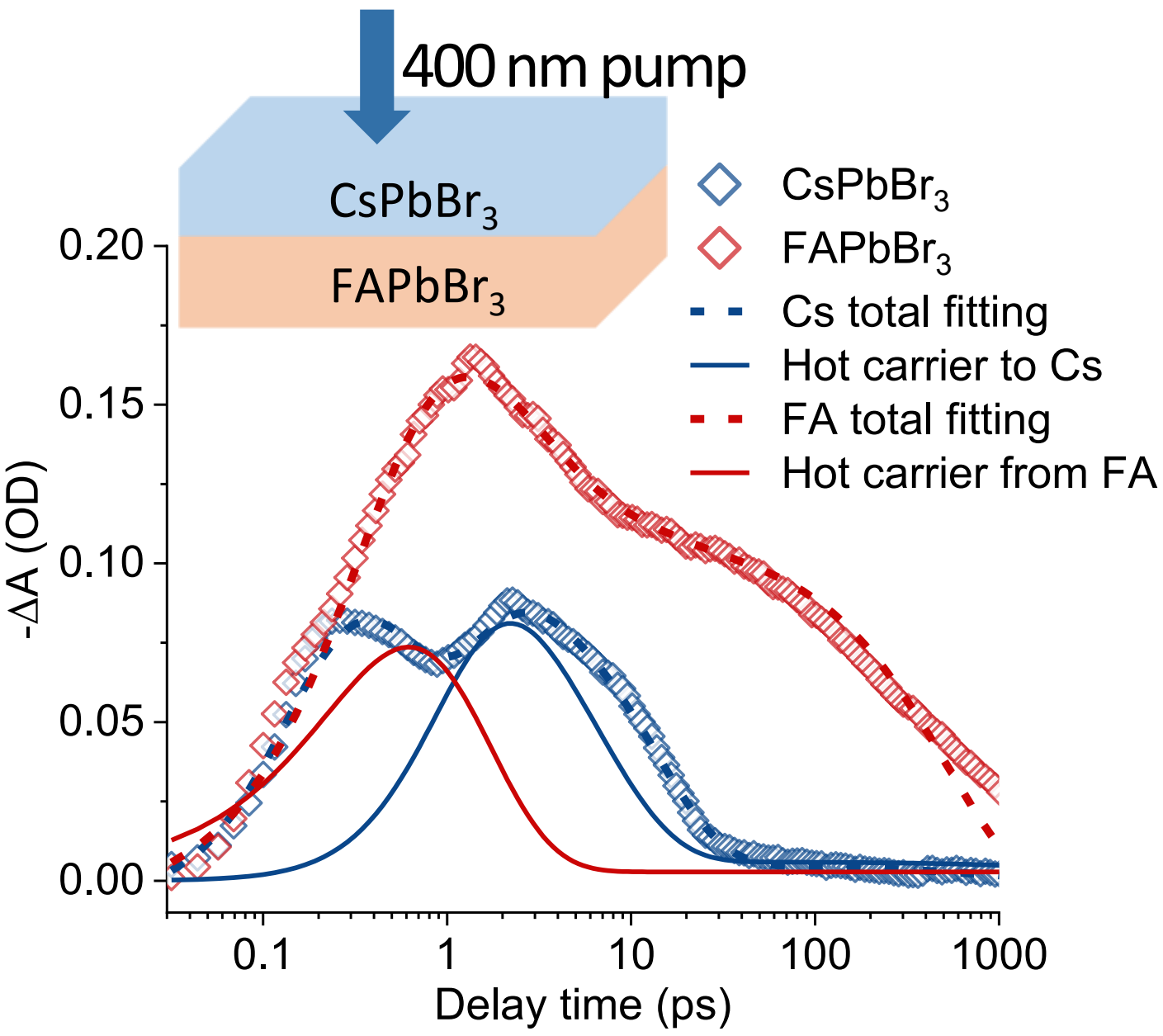

Fig. 3c, TA kinetics of laminated bilayer thin films. The pump laser (400 nm) was directed at $CsPbBr_3$ side. Global fitting was used to extract hot carrier transfer process in total TA signals (see details in Methods part). The dash line and solid line in (c) represent the fitting of total TA signals and the hot carrier transfer process, respectively.

We have ***added*** on the Page 10 of revised manuscript, that "As proposed in our earlier discussion of the hot carrier transfer mechanism (Fig. 1), when hot carriers are generated on both sides of the heterojunction, the differing cooling rates drive hot carriers toward the material with the faster cooling rate, such as $CsPbBr_3$. We globally fitted the TA kinetics at the $FAPbBr_3$ and $CsPbBr_3$ GSB positions using a sequential kinetic model to extract the hot-carrier transfer dynamics (see Methods for details). In Fig. 3c, the red and blue solid lines represent hot carriers transferred out of $FAPbBr_3$ and those injected into $CsPbBr_3$, respectively. Critically, the blue component constitutes the dominant fraction of the total carriers present in

$CsPbBr_3$, confirming that hot-carrier transfer is the primary source of population in the $CsPbBr_3$. At early times, hot carriers initially accumulate in the $FAPbBr_3$ phase due to the hot-phonon bottleneck effect, reaching a maximum at ~0.6 ps, whereas hot carriers in $CsPbBr_3$ cool very rapidly (<0.2 ps). This population imbalance drives directional hot-carrier transfer from $FAPbBr_3$ to $CsPbBr_3$, resulting in a pronounced hot-carrier population in $CsPbB_3$ that persists until ~2 ps and subsequently decays over approximately 10 ps. The fitted hot-carrier transfer time constant and carrier decay rates are summarized in Table S2. Notably, the hot-carrier population remains detectable in $CsPbBr_3$ for ~9.8 ps, which is considerably longer than the transfer out of $FAPbBr_3$ (~1.2 ps). This difference may be attributed to the finite transport time of hot carriers across the heterointerface."

We have ***added*** on the Page 11 of revised manuscript, that "After inserting the PMMA block layer, the GSB kinetics for both $CsPbBr_3$ and $FAPbBr_3$ (Fig. 3e, f) layers are close to those of the individual layers shown in Fig. 3a. These results further confirm that hot carrier transfer occurs within the laminated bilayers, and that the transfer is hindered when an intervening layer prevents direct interfacial interaction. Importantly, the hot-carrier transfer process persists for approximately 10 ps, which is fully consistent with our global fitting results (Fig. 3c)."

2. The manuscript assumes that under 530 nm resonant excitation, only FAPbBr3 is initially excited, and at low fluence no CsPbBr3 signal is observed, which the authors regard as consistent with the type-II alignment. However, if the interface indeed supports efficient interfacial transfer, then the hole-transfer channel from FAPbBr3 to CsPbBr3 should also be considered explicitly. In that case, one may also expect signatures associated with CsPbBr3 even under lower-power excitation. The current discussion focuses selectively on the proposed hot-electron pathway but does not rigorously analyze why other interfacial processes expected for a type-II junction are absent or negligible.

Our Response: We thank the reviewer for this important comment. A rigorous analysis of all potential interfacial processes is essential to substantiate our

mechanistic claim. Below we provide a systematic, timescale-based analysis of why the hot-carrier transfer pathway dominates while other processes (hole transfer from $FAPbBr_3$ to $CsPbBr_3$ and electron transfer from $CsPbBr_3$ to $FAPbBr_3$) are negligible under our experimental conditions.

The reviewer correctly notes that a conventional type-II junction typically drives both electrons and holes toward opposite layers. Our $CsPbBr_3/FAPbBr_3$ heterojunction indeed exhibits type-II band alignment; however, the conduction band offset (ΔCBM = 0.09 eV) is substantially larger than the valence band offset (ΔVBM = 0.02 eV). Similar perovskite heterostructures have been identified, through both theoretical calculations and experimental measurements, as possessing a quasi-type-II alignment (*JACS*, 140(46), 15675-15683 (2018).; *ACS Photonics*, 9(6), 2008-2014, (2022).; *Angew. Chem. Int. Ed.*, 64, e202506436 (2025).). A quasi-type-II alignment is characterized by partial spatial separation between electron and hole wavefunctions, meaning that significant wavefunction overlap exists. Consequently, electron transfer across the interface is much more favourable than hole transfer.

We ***added*** on the Page 11 of revised manuscript, that "Besides, as depicted in Fig. 1b, due to type II band alignment, in principle, the hole generated in $FAPbBr_3$ ~~would~~ may also jump to $CsPbBr_3$ VB maximum further increasing their carrier density. However, the conduction band offset (ΔCBM = 0.09 eV) in our $CsPbBr_3/FAPbBr_3$ heterojunction is substantially larger than the valence band offset (ΔVBM = 0.02 eV). Similar perovskite heterostructures have been identified, through both theoretical calculations and experimental measurements, as possessing a quasi-type-II alignment *(36-38)*. A quasi-type-II alignment is characterized by partial spatial separation between electron and hole wavefunctions, meaning that significant wavefunction overlap exists. Consequently, electron transfer across the interface is much more favourable than hole transfer. Although $CsPbBr_3$ has a high CBM compared to that of $FAPbBr_3$, we did not observe the conventional carrier transfer from $CsPbBr_3$ to $FAPbBr_3$~~, likely due to fast carrier recombination in $CsPbBr_3$ (<100 ps) before carrier reaching interface.~~ Global fitting of the hot-carrier transfer

dynamics reveals that both carrier cooling and hot-carrier injection into $CsPbBr_3$ are extremely fast (the transfer time constant is ~9.8 ps, while initial cooling occurs in <0.2 ps in $CsPbBr_3$). This enables efficient carrier accumulation in the $CsPbBr_3$ phase before any significant decay can take place. The TA decay kinetics further show that the carrier lifetime in $CsPbBr_3$ is only tens of ps (Table S2), which is much shorter than the hundreds of picoseconds observed in $FAPbBr_3$. Moreover, most conventional carrier transport across the bilayer heterostructure is reported to occur on timescales longer than 100 ps *(39-41)*. Any carriers that reach the conduction band edge of $CsPbBr_3$ recombine radiatively to contribute to optical gain before they can transfer back to $FAPbBr_3$. Thus, the hot-carrier (both electron and hole) transfer from slower-cooling $FAPbBr_3$ to fast-cooling $CsPbBr_3$ dominates while other processes (hole transfer from $FAPbBr_3$ to $CsPbBr_3$ and electron transfer from $CsPbBr_3$ to $FAPbBr_3$) are negligible under our experimental conditions (Fig. 1b)."

36 Zhang, X., Guo, Z., Li, R., Yu, J., Yuan, B., Chen, B., He, T. and Chen, R., Quasi-type II core–shell perovskite nanocrystals for improved structural stability and optical gain. *ACS Applied Materials & Interfaces*, 13(48), pp.58170-58178 (2021).

37. Imran, M., Peng, L., Pianetti, A., Pinchetti, V., Ramade, J., Zito, J., Di Stasio, F., Buha, J., Toso, S., Song, J. and Infante, I., Halide perovskite–lead chalcohalide nanocrystal heterostructures. *Journal of the American Chemical Society,* 143(3), pp.1435-1446 (2021).

38. Fang, Q., Shang, Q., Zhao, L., Wang, R., Zhang, Z., Yang, P., Sui, X., Qiu, X., Liu, X., Zhang, Q. and Zhang, Y., Ultrafast charge transfer in perovskite nanowire/2D transition metal dichalcogenide heterostructures. *The journal of physical chemistry letters*, 9(7), 1655-1662 (2018).

39. Fu, Y., Zheng, W., Wang, X., Hautzinger, M.P., Pan, D., Dang, L., Wright, J.C., Pan, A. and Jin, S., Multicolor heterostructures of two-dimensional layered halide perovskites that show interlayer energy transfer. *Journal of the American Chemical Society*, 140(46), 15675-15683 (2018).

40. Li, W., Hu, L., Ma, J., Jiang, C., Zhang, S., Chen, Y., Hu, J., Liu, X., Wu, T. and Li, D., Enhancing self-trapped exciton emission via energy transfer in two-

dimensional/quantum dot perovskite heterostructures. *ACS Photonics*, 9(6), 2008-2014, (2022).

41. C. Guo, J. Guo, W. Song, S. Peng, M. Sun, G. Xing, L. Yu, Atomic heterointerface engineering and nonequilibrium carrier dynamics for enhanced photocatalysis in halide perovskite/MoS_2 systems. *Angew. Chem. Int. Ed.*, 64, e202506436 (2025).

We have ***modified*** our schematic illustration in Fig. 1 to also include hot hole transfer in our $CsPbBr_3/FAPbBr_3$ heterojunction.

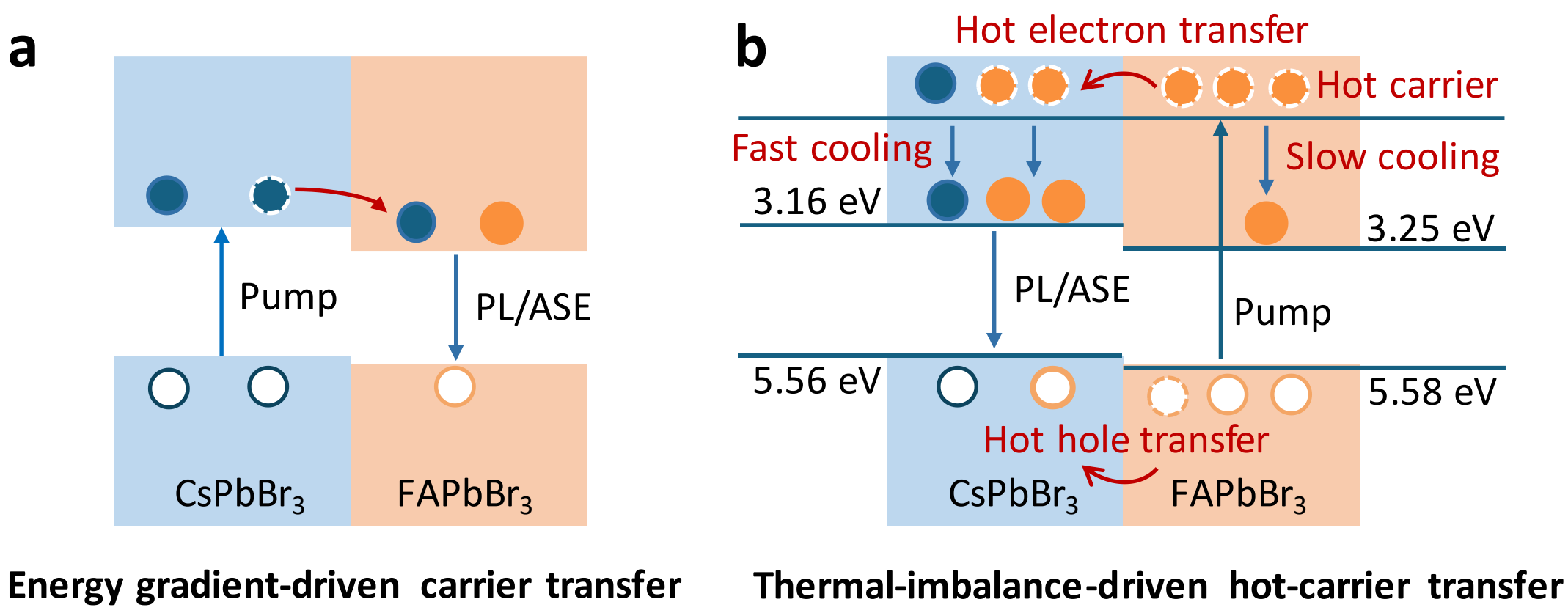

Figure 2. Carrier transfer mechanisms in semiconductor heterojunctions. a, Schematic illustration of conventional carrier transfer between high and low band gap semiconductors. Red arrows highlight the flow of cooled carriers across the interface driven by band energy gradient. **b**~~, c~~ Hot carrier transfer from a low band gap ($FAPbBr_3$) semiconductor to a high band gap semiconductor ($CsPbBr_3$). Under high-energy pumping conditions ~~(b)~~, hot carriers are generated in both high and low band gap semiconductors, while different hot carrier cooling rates influence the transfer dynamics, directing hot carriers into materials with faster cooling pathways. ~~With resonant pumping at low band gap semiconductor (c), hot carriers with excess energy above the band minimum (generated by scattering, Auger recombination or phase space filling) can move across the interface to the high band gap semiconductor.~~

We have ***added*** on the Page 17 of revised manuscript, that "At a relatively low

excitation fluence (~5 μJ/cm²), we only observe GSB signals at 533 nm (Fig. 5a, b), which we attribute to the band transitions of $FAPbBr_3$. No signals from $CsPbBr_3$ are detected~~, consistent with the Type II band alignment~~. Under the quasi-type-II band alignment, a small number of holes may transfer to the $CsPbBr_3$ side; however, in the absence of hot-electron transfer, these holes cannot recombine with electrons in $CsPbBr_3$ and therefore do not produce a detectable GSB signal."

3. Because the bandgaps of FAPbBr3 and CsPbBr3 are quite close, excitation nominally resonant with one component may still partially excite the other, especially under high fluence. In particular, under strong pumping, contributions from spectral tail absorption, two-photon absorption, phonon-assisted upconversion, or other nonlinear effects cannot be ruled out. This issue is especially important for the interpretation of the 530 nm resonant-pump TA data and the assignment of the delayed Cs-related signal to hot-carrier transfer induced by Auger heating. At present, the manuscript does not provide sufficient control experiments to exclude these competing possibilities. Fluence-dependent controls on the individual single-component films, together with excitation-wavelength-dependent measurements and nonlinear absorption controls, are needed.

Our Response: We thank the reviewer for this rigorous and constructive comment. We fully agree that a careful exclusion of direct excitation and nonlinear artefacts is essential for correctly assigning the $CsPbBr_3$ GSB signal observed under high fluence 530 nm pumping to hot-carrier transfer from $FAPbBr_3$. In the revised manuscript, we have performed the requested control experiments, as detailed below.

We have **performed** both wavelength-dependent and fluence-dependent TA measurements on control $CsPbBr_3$ single layer films.

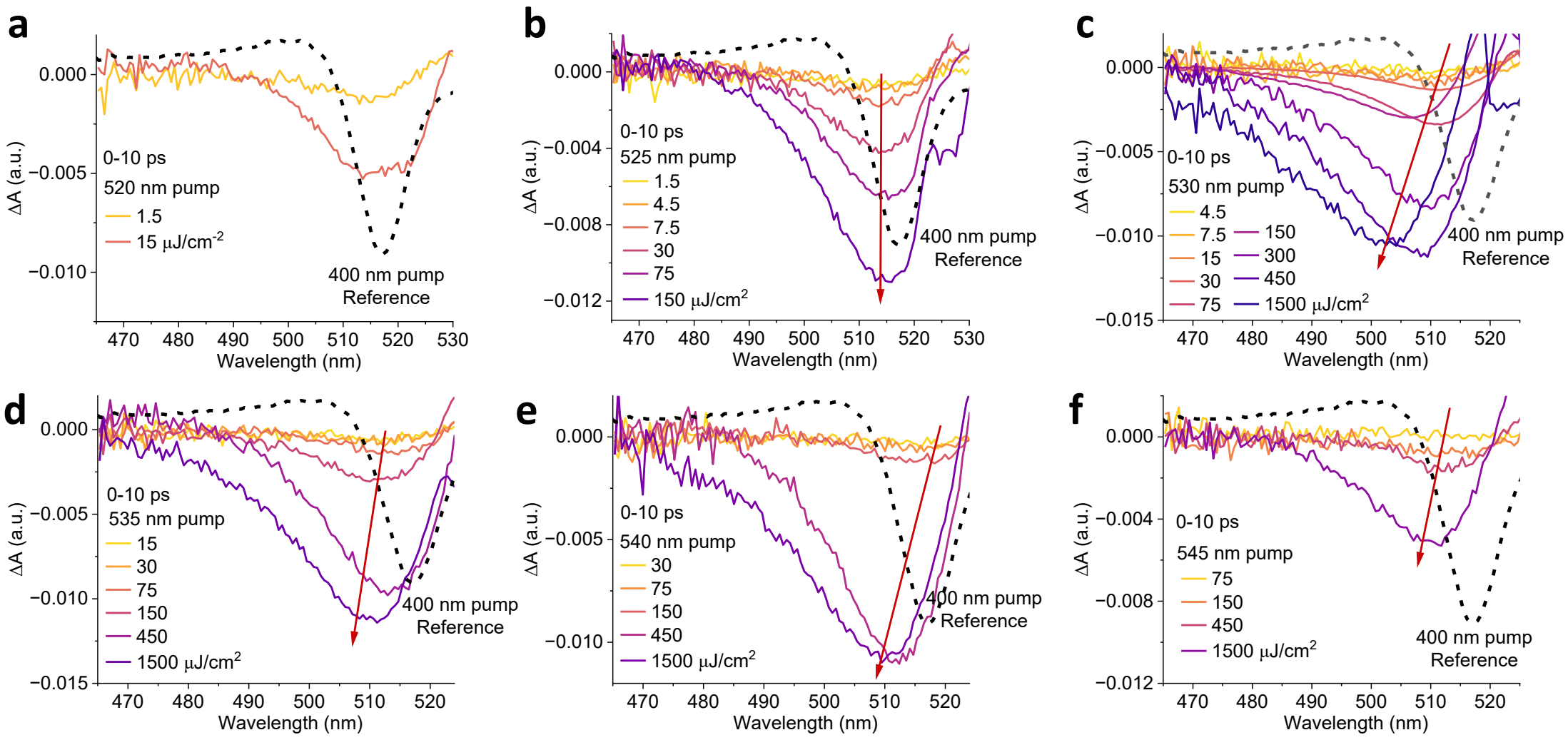

Figure S16. TA spectra of single-layer $CsPbBr_3$ as a function of pump fluence and wavelength. a-f, TA spectra (0–10 ps) at pump fluences ranging from 1.5 μJ/cm^2 to 1500 μJ/cm^2 and pump wavelengths from near-resonant (520 nm) to below-bandgap (525–545 nm).

We have ***added*** on the Page 18 of revised manuscript, that "Given that $CsPbB_3$ exhibits a higher bandgap than $FAPbBr_3$ (Fig. S1), and no GSB signal of $CsPbBr_3$ is observed under low-fluence excitation, the emergence of $CsPbBr_3$ signals at high excitation fluences provides strong evidence for hot carrier transfer from $FAPbBr_3$ to $CsPbBr_3$~~, resembling Scenario II (Fig. 1c)~~. To exclude possible contributions from nonlinear effects such as up-conversion or two-photon absorption, we performed wavelength- and fluence-dependent transient absorption (TA) measurements on control single-layer $CsPbBr_3$ films (Fig. S16; full mapping data are provided in Supporting Information Figs. S17–S22). When the pump wavelength was increased from 525 nm to 545 nm, the TA GSB signals exhibited a gradual blue shift and the threshold fluence for the appearance of a detectable GSB increased from 7.5 μJ/cm^2 to 150 μJ/cm^2. This behaviour is characteristic of an anti-Stokes up-conversion process. Critically, even at an extremely high pump fluence of 1500 μJ/cm^2 on the single-layer $CsPbBr_3$ film at 530 nm, the GSB signal remained below 0.012 – an order of magnitude lower than the GSB intensity observed in the laminated heterojunction under much lower excitation (Fig. 5d, e). Further, the

blue-shifted signals in the control experiment confirm the anti-Stokes nature of the up-conversion process, whereas in the heterojunction (Fig. 5d, e) the $CsPbBr_3$ GSB appears at its expected spectral position (~520 nm) without any blue shift. This unambiguously demonstrates that the $CsPbBr_3$ GSB in the heterojunction originates from carrier transfer from the $FAPbBr_3$ layer, and that the carrier density transferred into $CsPbBr_3$ is much higher than the carrier density achievable by direct excitation of $CsPbBr_3$ alone.”

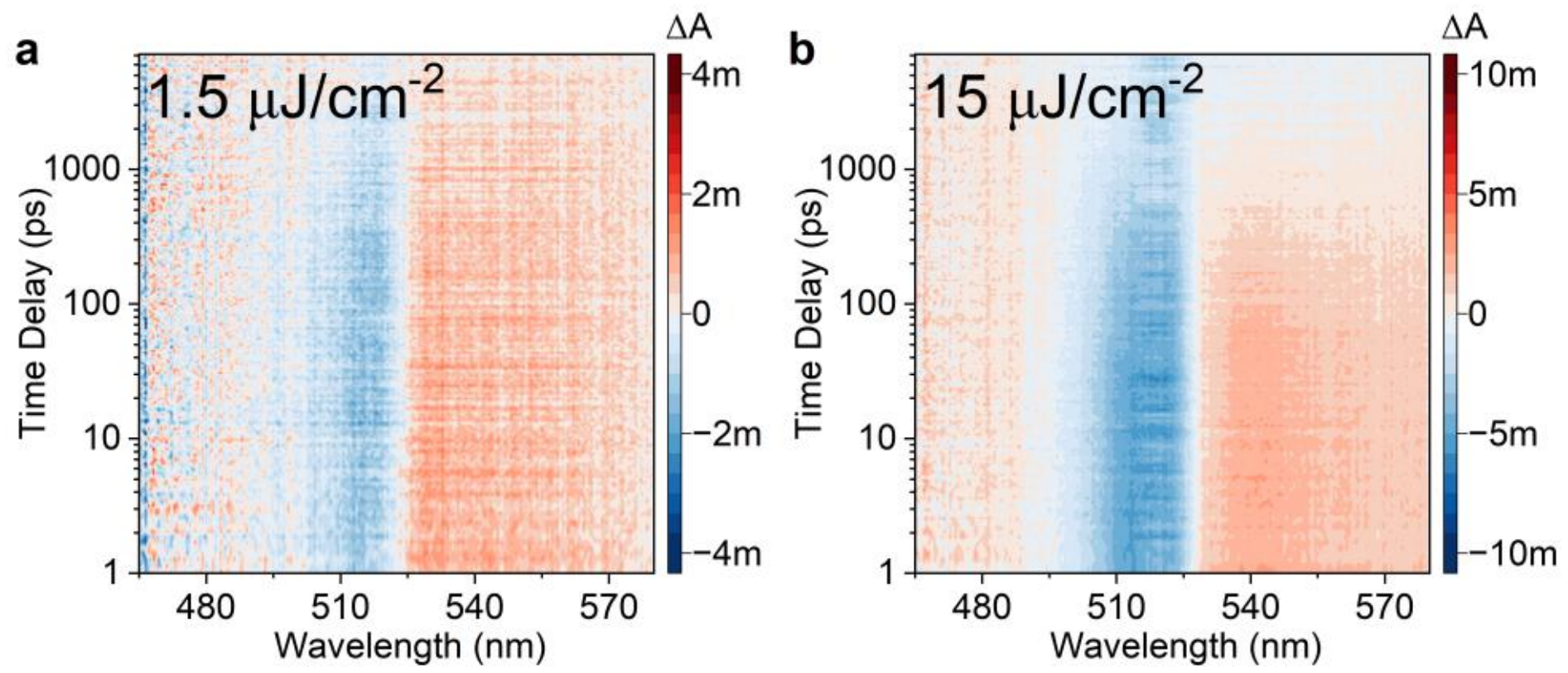

Figure S17. Fluence dependent transient absorption (TA) spectra of single layer $CsPbBr_3$ films. a-i, TA maps obtained from single layer $CsPbBr_3$ films. The pump fluence was increased from low value 1.5 to ultrahigh value 15 μJ/cm^2. A 520 nm pulsed laser was used here to excite the samples

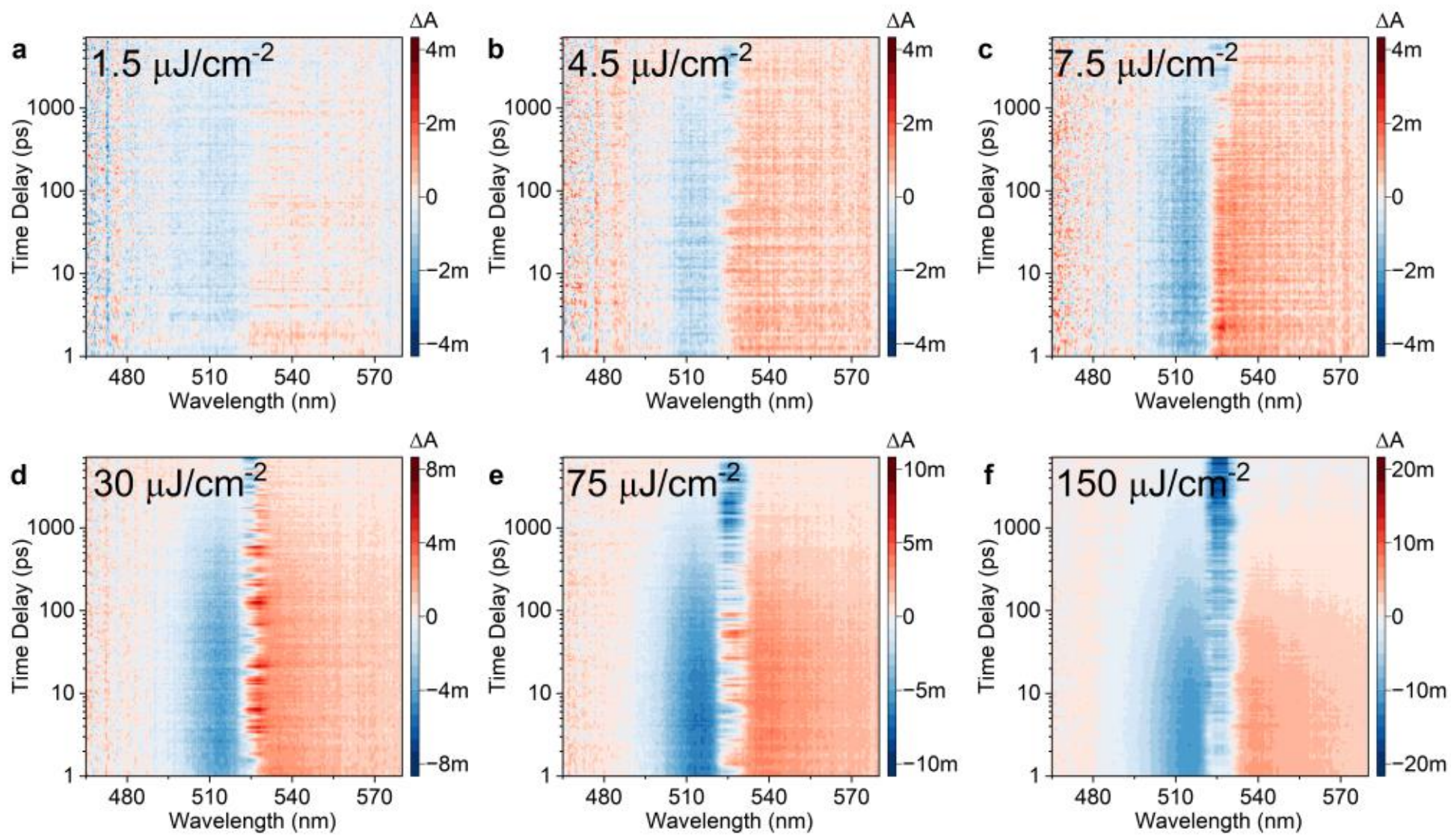

Figure S18. Fluence dependent transient absorption (TA) spectra of single layer $CsPbBr_3$ films. a-i, TA maps obtained from single layer $CsPbBr_3$ films. The pump fluence was increased from low value 1.5 to ultrahigh value 150 μJ/cm^2. A 525 nm

pulsed laser was used here to excite the samples.

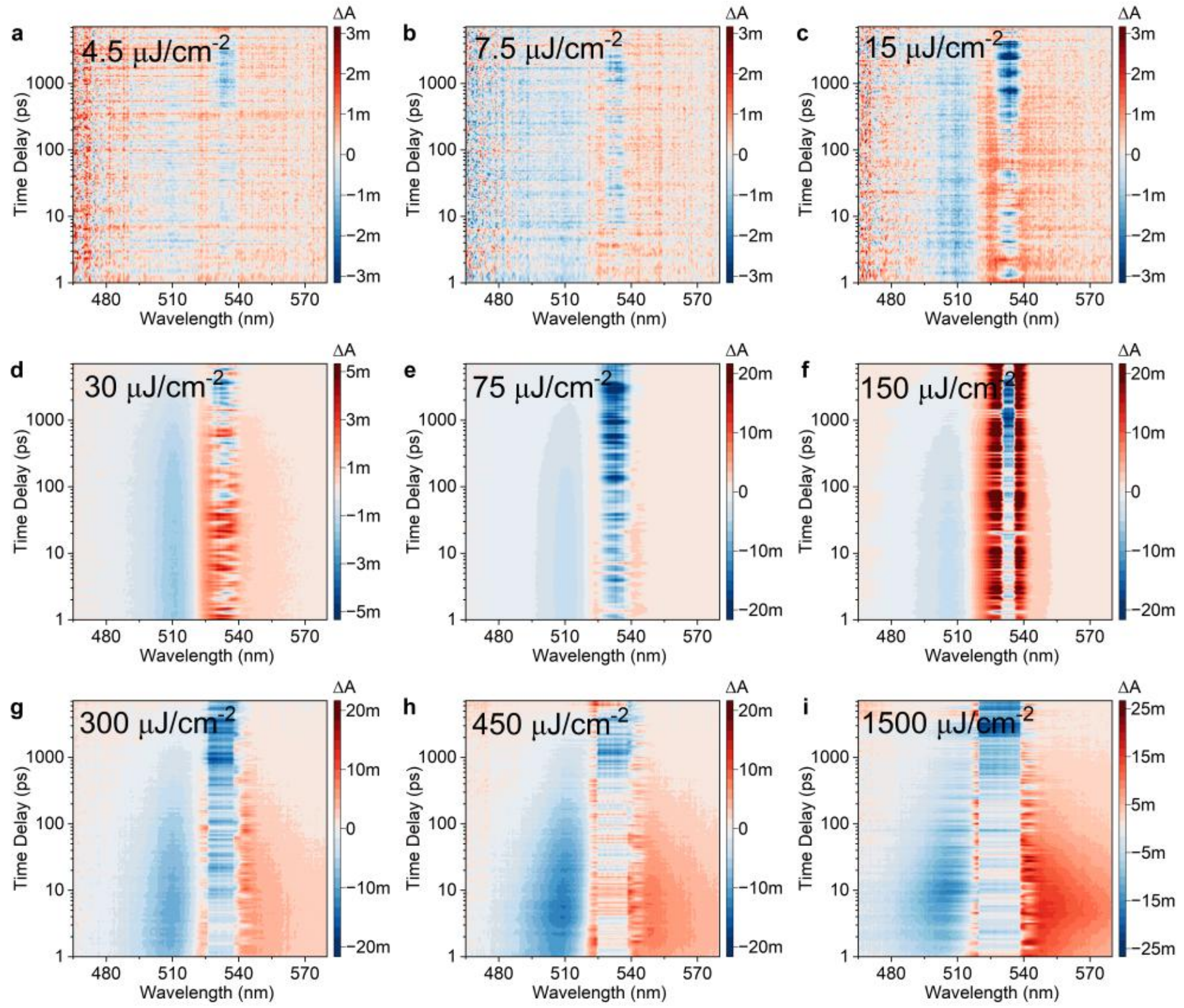

Figure S19. Fluence dependent transient absorption (TA) spectra of single layer $CsPbBr_3$ films. a-i, TA maps obtained from single layer $CsPbBr_3$ films. The pump fluence was increased from low value 4.5 to ultrahigh value 1500 μJ/cm^2. A 530 nm pulsed laser was used here to excite the samples.

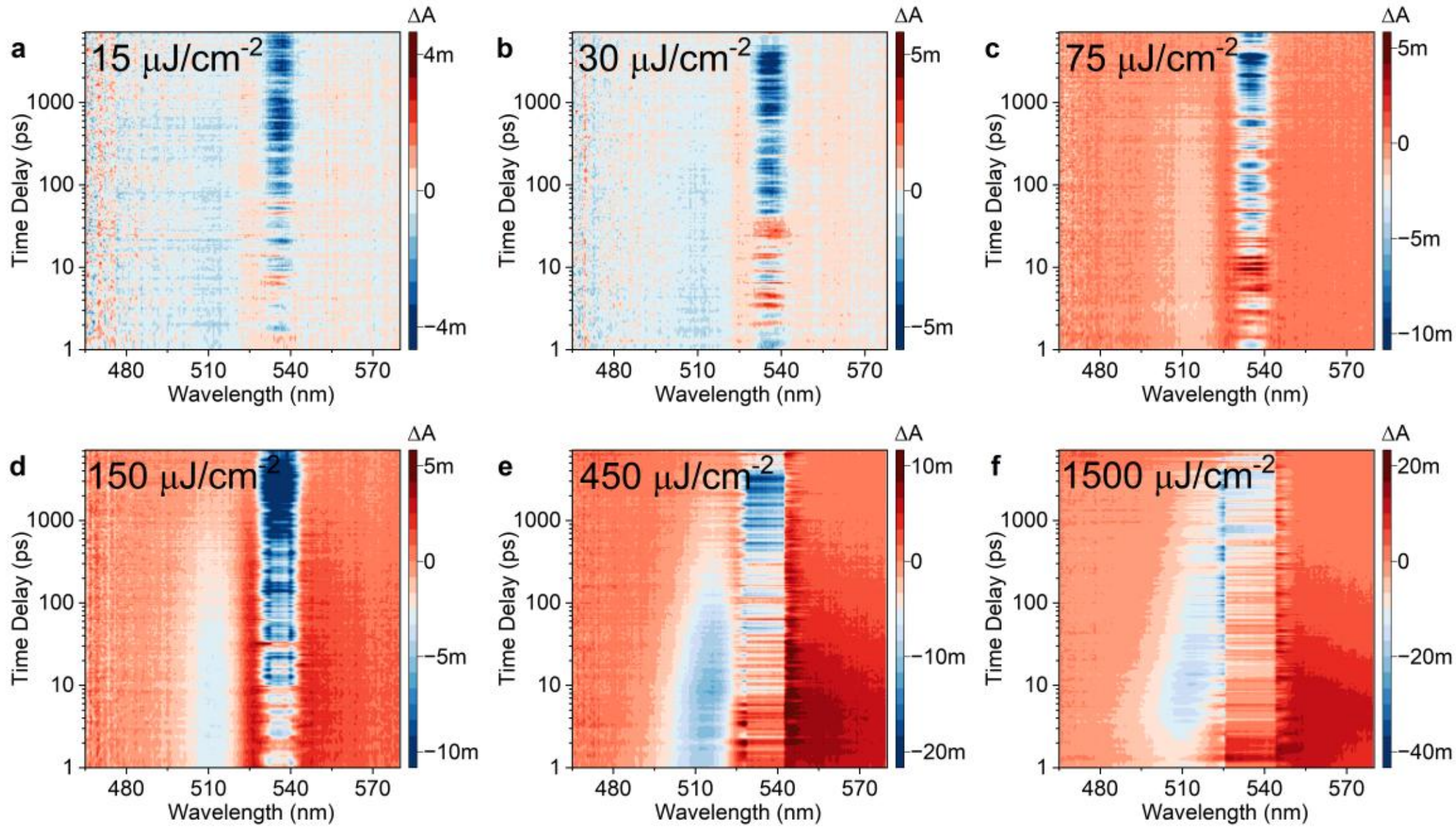

Figure S20. Fluence dependent transient absorption (TA) spectra of single layer $CsPbBr_3$ films. a-i, TA maps obtained from single layer $CsPbBr_3$ films. The pump fluence was increased from low value 15 to ultrahigh value 1500 μJ/cm^2. A 535 nm pulsed laser was used here to excite the samples.

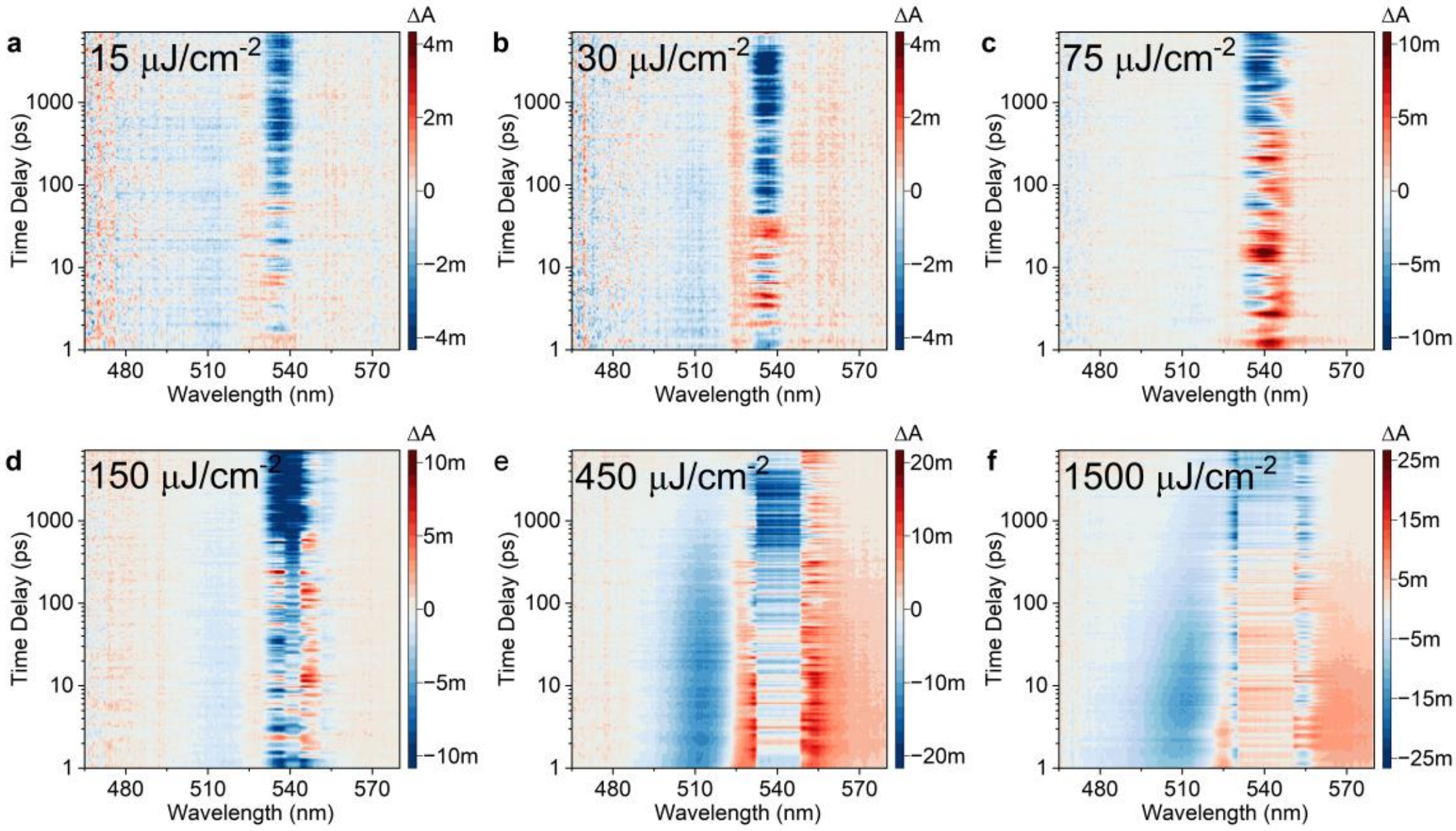

Figure S21. Fluence dependent transient absorption (TA) spectra of single layer $CsPbBr_3$ films. a-i, TA maps obtained from single layer $CsPbBr_3$ films. The pump fluence was increased from low value 15 to ultrahigh value 1500 μJ/cm^2. A 540 nm pulsed laser was used here to excite the samples.

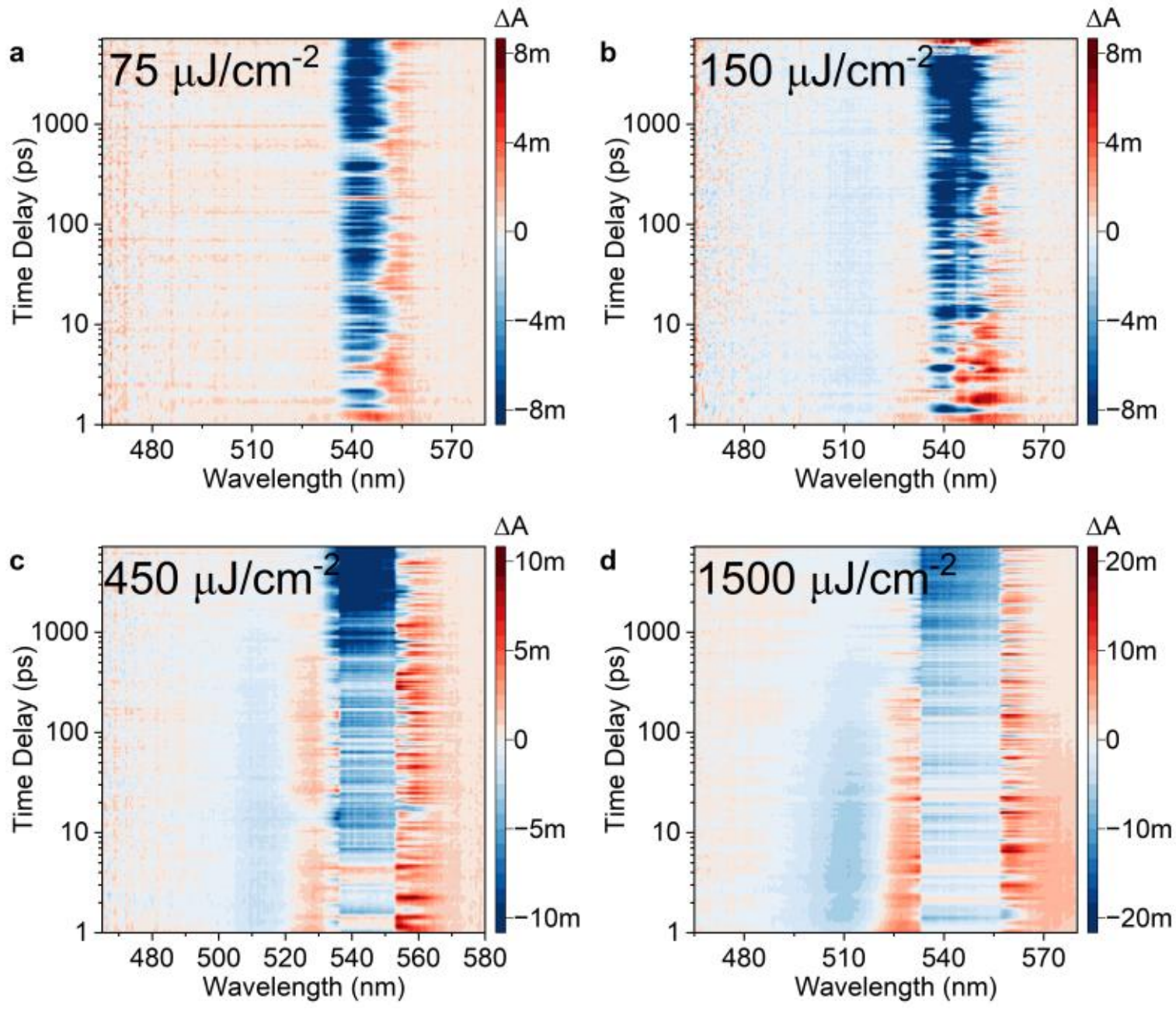

Figure S22. Fluence dependent transient absorption (TA) spectra of single layer $CsPbBr_3$ films. a-i, TA maps obtained from single layer $CsPbBr_3$ films. The pump fluence was increased from low value 75 to ultrahigh value 1500 μJ/cm^2. A 545 nm pulsed laser was used here to excite the samples.

4. The manuscript attributes the lower ASE and lasing thresholds primarily to hot-carrier transfer. However, ASE and lasing thresholds are also highly sensitive to film morphology, roughness, cavity quality, thickness uniformity, interface flatness, and optical confinement. In fact, the authors themselves show that lamination dramatically changes the film morphology and makes the bilayer much flatter and denser. Therefore, it is difficult to separate the effect of carrier-transfer physics from improvements in cavity or scattering loss. This concern is particularly serious because the threshold differences between the pristine films and heterostructures are not supported by any statistical analysis across multiple devices or batches. The manuscript should provide device-to-device statistics, error bars, and sample numbers before drawing a strong conclusion that the reduced threshold originates predominantly from hot-carrier transfer.

Our Response: We are thankful for this insightful comment, which aligns with a question from another reviewer (Reviewer 3 Comments 3). To ensure each reviewer receives the same complete answer, we reproduce our full response here.

We have now ***performed*** statistical analysis on more than 5 devices per condition.

We further **compared** the ASE thresholds of laminated $CsPbBr_3$/$FAPbBr_3$ heterojunction and a control sample with a PMMA interlayer inserted between the two perovskite layers. Both samples underwent the identical lamination process; however, the PMMA layer blocks hot-carrier transfer, thereby disabling the transfer pathway.

We have ***plotted*** below and in revised manuscript Fig. S12 all ASE threshold results with error bars indicated.

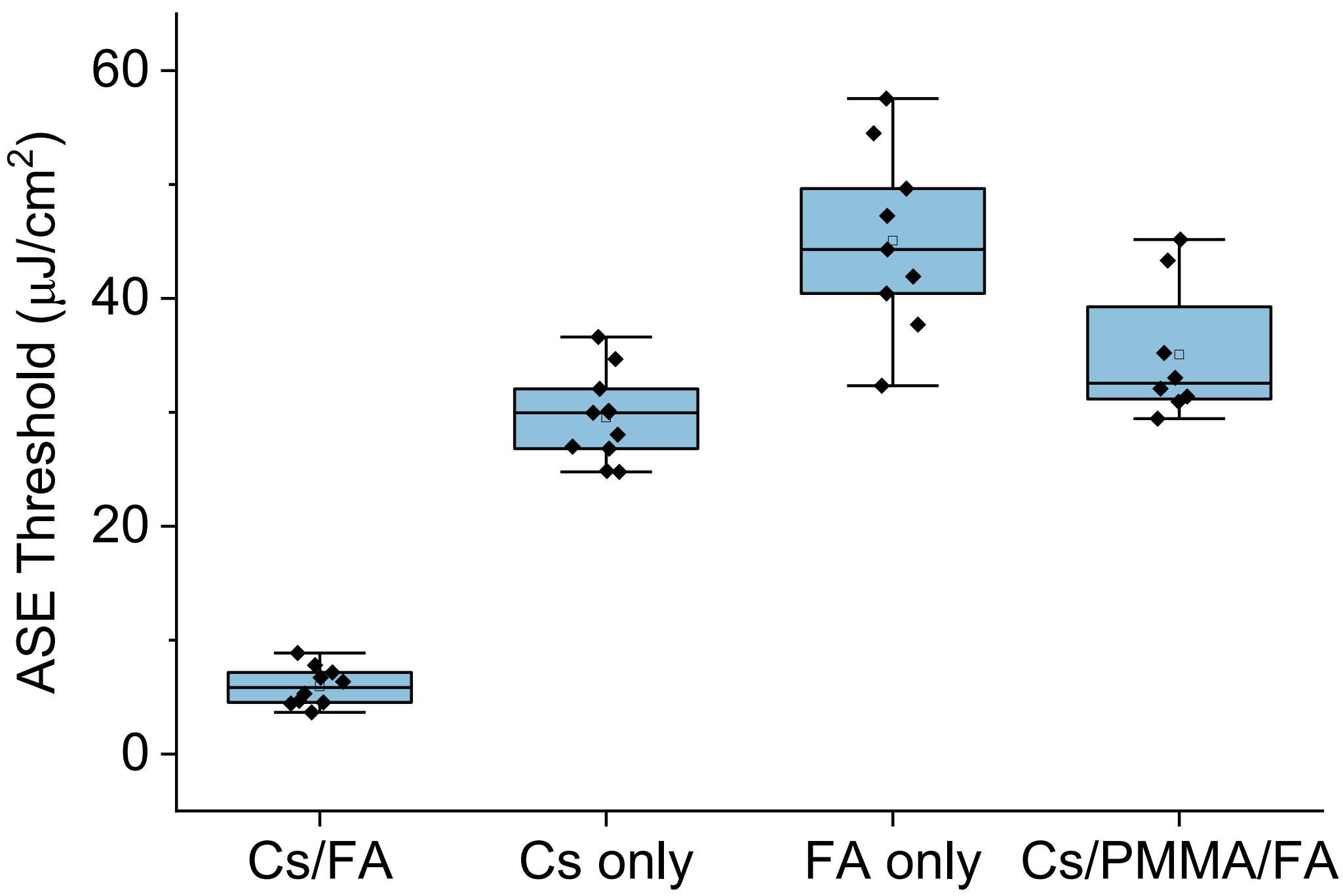

Figure S12. Comparison of amplified spontaneous emission (ASE) thresholds. ASE thresholds were measured for the laminated $CsPbBr_3/FAPbBr_3$ heterojunction (Cs/FA), single-layer $CsPbBr_3$ (Cs only), single-layer $FAPbBr_3$ (FA only), and a control sample with a PMMA interlayer inserted between the two perovskite layers during the lamination process (Cs/PMMA/FA). Each condition was measured on at least five devices (individual data points shown as solid squares). A 355 nm nanosecond pulsed laser was used for excitation.

We have ***added*** on the Page 15 of revised manuscript, that "Using 355 nm nanosecond pulsed excitation (1 ns pulse width, 1 kHz repetition rate) laminated $CsPbBr_3$ samples exhibit a substantially lower ASE threshold (~4 μJ/cm²) compared to individual samples (Fig. 4a). Statistical analysis across multiple devices confirms the reproducibility of this threshold reduction (Fig. S12). We further compared the ASE thresholds of laminated $CsPbBr_3/FAPbBr_3$ heterojunction and a control sample with a PMMA interlayer inserted between the two perovskite layers. Both samples underwent the identical lamination process; however, the PMMA layer blocks

hot-carrier transfer, thereby disabling the hot transfer pathway. Consequently, the control sample exhibited no ASE threshold reduction relative to single-layer $CsPbBr_3$ (Fig. S12), confirming that the threshold reduction in the laminated heterojunction is primarily attributable to hot-carrier transfer."

The observed reduction in ASE threshold is not attributable to differences in cavity quality. However, changes in defect states could also lead to a significant decrease in ASE thresholds after lamination. To exclude this possibility, we further ***performed*** control experiments to rule out the possible contributions from defect states. Notably, we find that the PLQE values for the laminated structure does not decrease below the single layer values (revised Fig. S7), excluding an impact from potential defects at the interface to the light emission process.

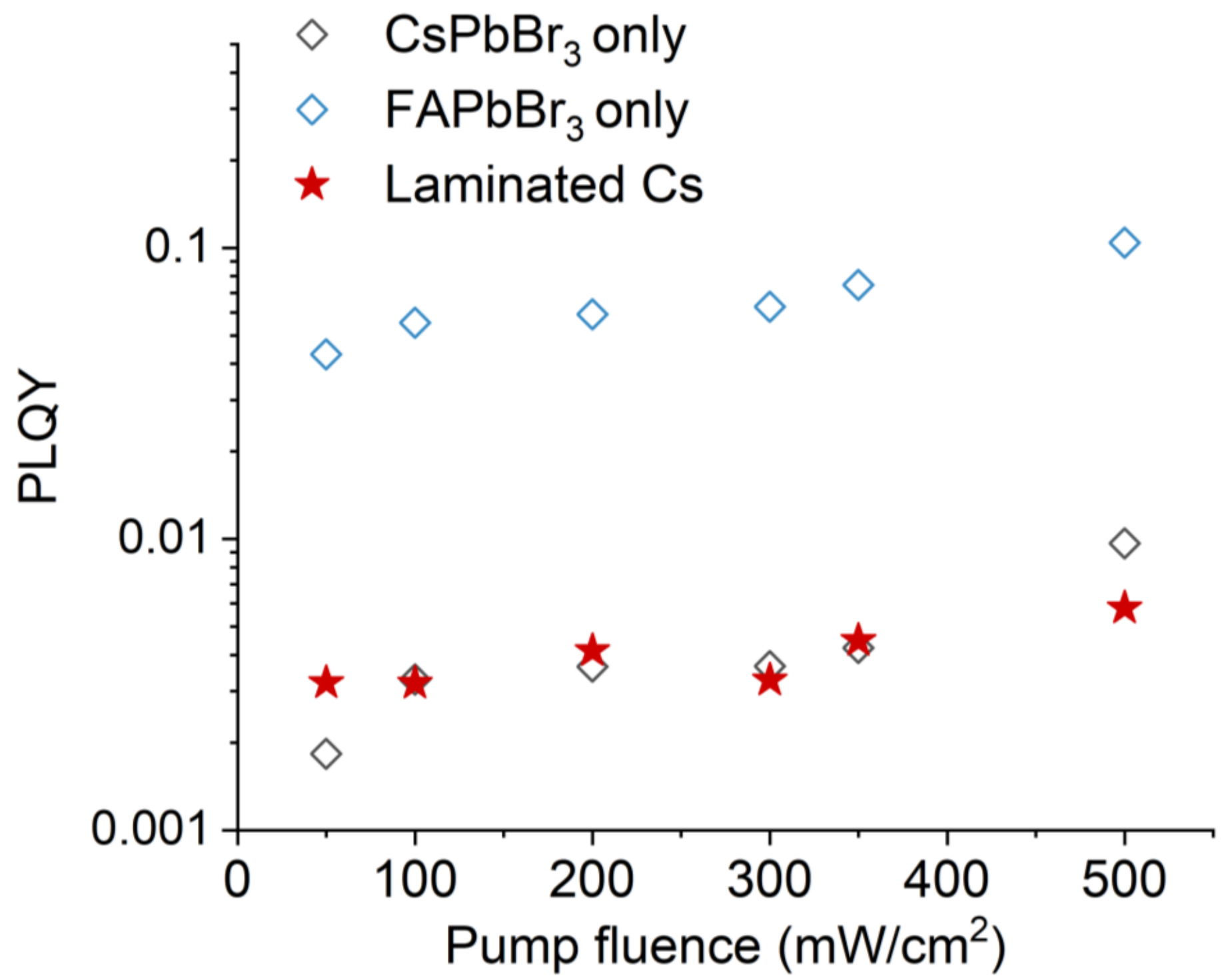

Figure S7. Comparison of photoluminescence quantum yield (PLQY) results before and after lamination process ~~$CsPbBr_3$ and $FAPbBr_3$ films~~. The samples were excited with a 400 nm continuous wave laser, and a ND filter wheel was used to control the excitation fluences. $FAPbBr_3$ films exhibit a PLQY that is at least ten

times higher than that of $CsPbBr_3$ films at all pump powers. Importantly, after the lamination process, the PLQY of the $CsPbBr_3$ films remains almost unchanged compared to their pre-lamination values, further confirming that the defect density is not altered by lamination.

We have ***added*** the discussion on the Page 12 of revised manuscript, that "Recent studies have suggested that defect states could also form a three-level system, which might facilitate population inversion *(45-47)*. In our work, however, the heterojunction itself inherently provides a multi-level structure. The three-level laser picture we propose is grounded in the band alignment and hot carrier transfer in the $CsPbBr_3/FAPbBr_3$ heterojunction, not in the presence of defect states. Consequently, defects are not required to explain the observed population inversion or the reduction in lasing threshold. To further rule out possible contributions from defect states, we compared the excited-state carrier dynamics before and after the lamination process. In both TA and time-resolved photoluminescence (TRPL) spectra, we observed blue-shifted signals on the high-energy tail at early times after lamination (Fig. S9 and S10), indicating the contribution of hot carriers transferred into the $CsPbBr_3$ side *(48-50)*. At later times (beyond 1 ns), both TA and TRPL spectra overlapped well on the low-energy tail, confirming that no additional trap states were introduced and that existing trap states were not strongly passivated by lamination.

Notably, under equivalent low carrier generation densities (Fig. S9c), the $CsPbBr_3$ ground-state bleach (GSB) signals before and after lamination exhibited similarly rapid decays over the first few picoseconds – a feature typically attributed to trap-mediated recombination *(51, 52)* – indicating comparable trap densities. Moreover, at both low and high carrier generation densities, the $CsPbBr_3$ GSB decay kinetics remained nearly identical before and after lamination, further demonstrating that the defect density is essentially unchanged by the lamination process. Consistently, after lamination, the $CsPbBr_3$ PL decay showed a prolonged lifetime during the initial 4 ns (Fig. 10c and f), which we attribute to fast hot-carrier transfer from the $FAPbBr_3$ layer. Beyond 4 ns, the decay kinetics before and after

lamination became almost overlapped, confirming that the density of long-lived trap states remains unaffected *(53)*. Further, the PLQY of $CsPbBr_3$ films remained nearly unchanged after lamination compared to their pre-lamination values (Fig. S7). Taking together, all these results demonstrate that the lamination process does not significantly alter the defect density in $CsPbBr_3$. Therefore, the dominant mechanism responsible for reducing the ASE threshold in the laminated heterojunctions is hot-carrier transfer driven by cooling-rate gradients, not defect-mediated processes."

45. Wu, C. S. J., Wong, E. L., Li, H., Jiménez-López, J., Lin, C. K., Hsu, H. C., & Petrozza, A. Impact of Nano-Scale Defects on the Macroscopic Amplified Spontaneous Emission in Polycrystalline Perovskite Thin-Films. Advanced Materials, 38(24), e16903 (2026).
46. Zhang, L., Wang, Y., Chu, A. *et al.* Facet-selective growth of halide perovskite/2D semiconductor van der Waals heterostructures for improved optical gain and lasing. *Nat Commun* **15**, 5484 (2024).
47. S. Huang, Z. Shen, Y. Liao, Z. Liu, Z. Hu, Q. Li, Z. Zhang, S. Dong, J. Luo, J. Du, J. Tang, Y. Leng, Water-Resistant Subwavelength Perovskite Lasing from Transparent Silica-Based Nanocavity. Adv. Mater., 35, 2306102 (2023).
48. Righetto, M., Lim, S.S., Giovanni, D. *et al.* Hot carriers perspective on the nature of traps in perovskites. *Nat Commun* **11**, 2712 (2020).
49. Ye, J., Mondal, N., Carwithen, B.P. *et al.* Extending the defect tolerance of halide perovskite nanocrystals to hot carrier cooling dynamics. *Nat Commun* **15**, 8120 (2024).
50 L. Zhang, W. Xi, H. Zhang, et al. Efficient Hot Hole Extraction in Halide Perovskite/2D Semiconductor van der Waals Heterostructures. *Adv. Funct. Mater.*36, 22, e17023 (2026).
51. Fan, H., Li, F., Wang, P. *et al.* Methylamine-assisted growth of uniaxial-oriented perovskite thin films with millimeter-sized grains. *Nat Commun* **11**, 5402 (2020).
52. Wang, X., Jin, L., Sergeev, A., Liu, W., Gu, S., Li, N., Fan, K., Chen, S.C., Wong, K.S., Sun, X. and Zhao, N., Quasi-2D Dion-Jacobson phase perovskites as a

promising material platform for stable and high-performance lasers. *Science Advances*, 9(43), eadj3476 (2023).

53. Fang, HH., Adjokatse, S., Shao, S. et al. Long-lived hot-carrier light emission and large blue shift in formamidinium tin triiodide perovskites. Nat Commun 9, 243 (2018).

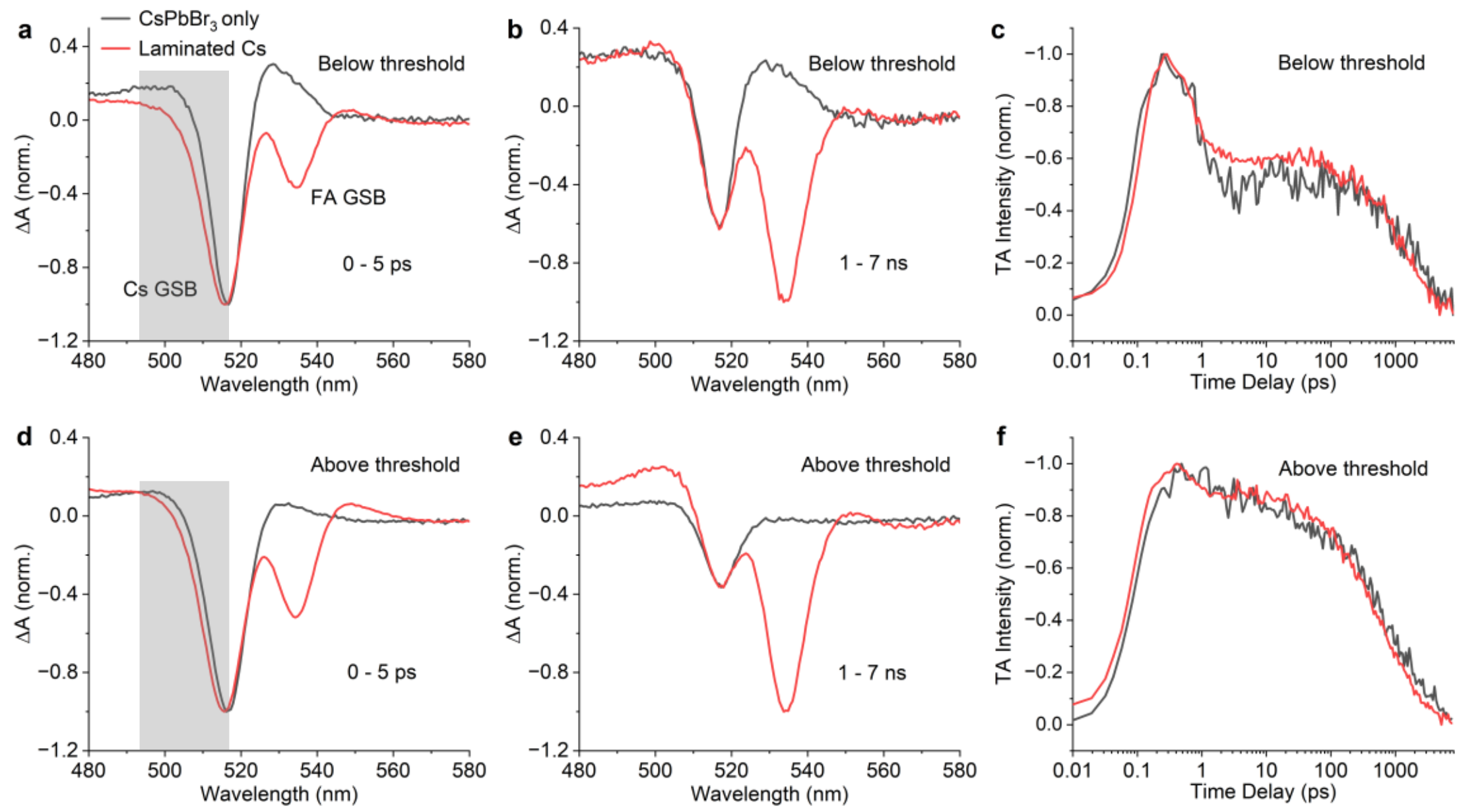

Figure S9. Comparison of transient absorption spectra before and after lamination process. a-c, Low carrier density generation regime (below ASE threshold), TA spectra (a, 0-5 ps; b, 1-7 ns) and kinetics of $CsPbBr_3$ GSB (c) before and after lamination. d-f, High carrier density generation regime (above ASE threshold), TA spectra (a, 0-5 ps; b, 1-7 ns) and kinetics of $CsPbBr_3$ (c) before and after lamination. Notably, at equivalent carrier generation densities, the $CsPbBr_3$ GSB decay kinetics exhibit nearly identical behavior before and after lamination, indicating that the defect density remains essentially unchanged by the lamination process.

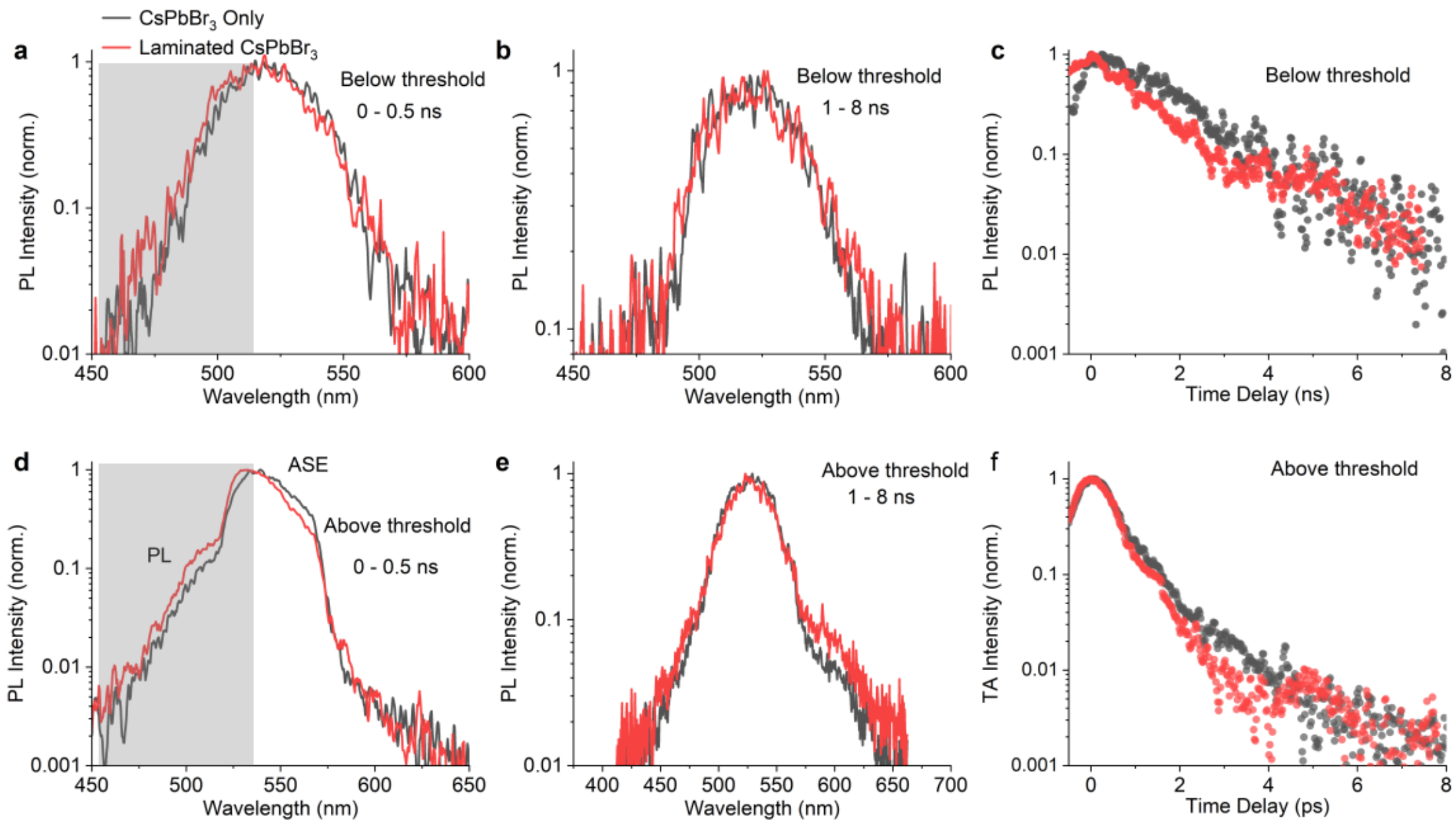

Figure S10. Comparison of time-resolved PL spectra before and after lamination process. a-c, With low excitation fluence (below ASE threshold), PL spectra (a, 0-5 ns; b, 1-8 ns) and decay kinetics of $CsPbBr_3$ (c) before and after lamination. d-f, With high excitation fluence (above ASE threshold), PL spectra (a, 0-5 ns; b, 1-8 ns) and decay kinetics of $CsPbBr_3$ (c) before and after lamination. Time resolved PL spectra were obtained by streak camera. After lamination, the $CsPbBr_3$ PL decay exhibits a prolonged lifetime during the initial 4 ns, which we attribute to fast hot-carrier transfer from the $FAPbBr_3$ layer. Beyond 4 ns, the decay kinetics before and after lamination become nearly overlapped, confirming that the lamination process does not alter the density of long-lived trap states.

5. The cross-sectional SEM images show that the pristine CsPbBr3 and FAPbBr3 films are presented on ITO substrates, whereas the laminated bilayer is shown on glass. At minimum, the manuscript should explain clearly why the substrate changes between these panels. In addition, comparing Fig. 2e and 2f, the morphology of the FAPbBr3 layer undergoes a dramatic transformation, becoming extremely dense and flat after lamination. What causes such a profound morphological change through a simple solid-state pressing process?

Our response: We greatly appreciate this comment, which converges with a point

made by another reviewer (Reviewer 3 comments 1). Accordingly, our answer is repeated below verbatim, and we thank you for prompting this important clarification.

For SEM measurements using the ITO and glass substrates, we thank the reviewer for pointing out this inconsistency, and we apologize for not providing a clear explanation in the original manuscript. The choice of substrate was dictated by practical considerations for cross-sectional SEM imaging of the delicate laminated bilayer, not by any intended difference in film growth.

In the lamination process, a $CsPbBr_3$ film and a $FAPbBr_3$ film are pressed face-to-face. After lamination, the two substrates must be separated to expose the bilayer interface for cross-sectional imaging. We found that the perovskite films adhere more strongly to glass than to ITO. Therefore, when we laminate a $CsPbBr_3$ film on glass with a $FAPbBr_3$ film on ITO (or vice versa), and then separate the two substrates, the bilayer structure remains intact on the glass side, while the ITO substrate detaches cleanly. This allowed us to obtain a clean cross-section of the intact $CsPbBr_3$/$FAPbBr_3$ bilayer for SEM imaging (Fig. 2f). In contrast, if both substrates were ITO, the bilayer often tore or delaminated during separation, preventing reliable cross-sectional imaging.

We acknowledge that the growth morphology of each individual perovskite layer may differ slightly between glass and ITO. However, for the purpose of Figure 2f, the key information is the existence of a continuous bilayer with a well-defined interface, not the absolute grain size or roughness of the individual layers.

We have ***added*** on the Method section of revised manuscript, that "Field emission top-view and cross-section SEM images of perovskite films were taken with a ZEISS Supra60 VP scanning electron microscope. For cross-sectional SEM imaging of the laminated bilayer, the $CsPbBr_3$ and $FAPbBr_3$ films were deposited on glass and ITO, respectively. After lamination, the two substrates were separated; the bilayer adhered preferentially to the glass side, enabling clean cross-sectional imaging."

The reviewer rightly notes that the top-view morphology of $CsPbBr_3$ changes dramatically from a rough, partially covered film (Fig. 2g) to a smooth, dense, ultra-small-grain film (Fig. 2i) after lamination. We have now provided a clear explanation and control experiments for this evolution.

We have ***added*** on the Page 7 of revised manuscript, that "Notably, prior to lamination, both $CsPbBr_3$ and $FAPbBr_3$ exhibit only partial coating of the substrate~~, while after lamination, additional heat and pressure drive the recrystallization of perovskite films, resulting in flat and uniform bilayer films. The same trends were observed in top-view SEM images (Fig. 2g-i). After lamination, $CsPbBr_3$ films become very uniform and smooth, with grain size below 10 nm resolution and improved surface roughness~~. The lamination process applies elevated temperature (120 °C) and high pressure (100 MPa) for 5 min. Under these conditions, the perovskite layer undergoes pressure-induced recrystallization. The combined heat and pressure promote atomic rearrangement, grain rotation, and coalescence, resulting in a much smoother, denser film with grain sizes below resolution (Fig. 2g-i). Similar recrystallization phenomena have been reported in laminated perovskite films by several groups *(27–30)*. Control experiments reveal that hot pressing a $CsPbBr_3$ film against a silicon wafer (i.e., without a second perovskite layer) improves its surface roughness, but the smoothing effect is substantially weaker than that observed after lamination with a $FAPbBr_3$ layer (Fig. S2). Additionally, lamination of thinner films produces a less pronounced morphological change. These observations indicate that the dramatic transformation is driven by recrystallization at the interface when two thick perovskite films are brought into intimate contact under heat and pressure. Importantly, this recrystallization does not degrade their structure and optical properties; as shown in Fig. 2b and 2c, the absorption spectrum and XRD of perovskite films are unchanged after lamination."

27. R. Schmager, J. Roger, J. A. Schwenzer, F. Schackmar, T. Abzieher, M. Malekshahi Byranvand, B. Abdollahi Nejand, M. Worgull, B. S. Richards, U. W. Paetzold, Laminated perovskite photovoltaics: enabling novel layer

combinations and device architectures. *Adv. Funct. Mater.* 30, 1907481 (2020).

28. J. Roger, L. K. Schorn, M. Heydarian, A. Farag, T. Feeney, D. Baumann, H. Hu, F. Laufer, W. Duan, K. Ding, A. Lambertz, P. Fassl, M. Worgull, U. W. Paetzold, Laminated monolithic perovskite/silicon tandem photovoltaics. *Adv. Energy Mater.* 12, 2200961 (2022).
29. Y. Li, J. Roger, I. Allegro, J. C. Fischer, Q. Jin, U. Lemmer, I. A. Howard, U. W. Paetzold, Lasing from laminated quasi-2D/3D perovskite planar heterostructures. *Adv. Funct. Mater.* 32, 2200772 (2022).
30. S. P. Dunfield, D. T. Moore, T. R. Klein, D. M. Fabian, J. A. Christians, A. G. Dixon, B. Dou, S. Ardo, M. C. Beard, S. E. Shaheen, J. J. Berry, M. F. A. M. Van Hest, Curtailing perovskite processing limitations via lamination at the perovskite/perovskite interface. *ACS Energy Lett.* 3, 1192–1197 (2018)

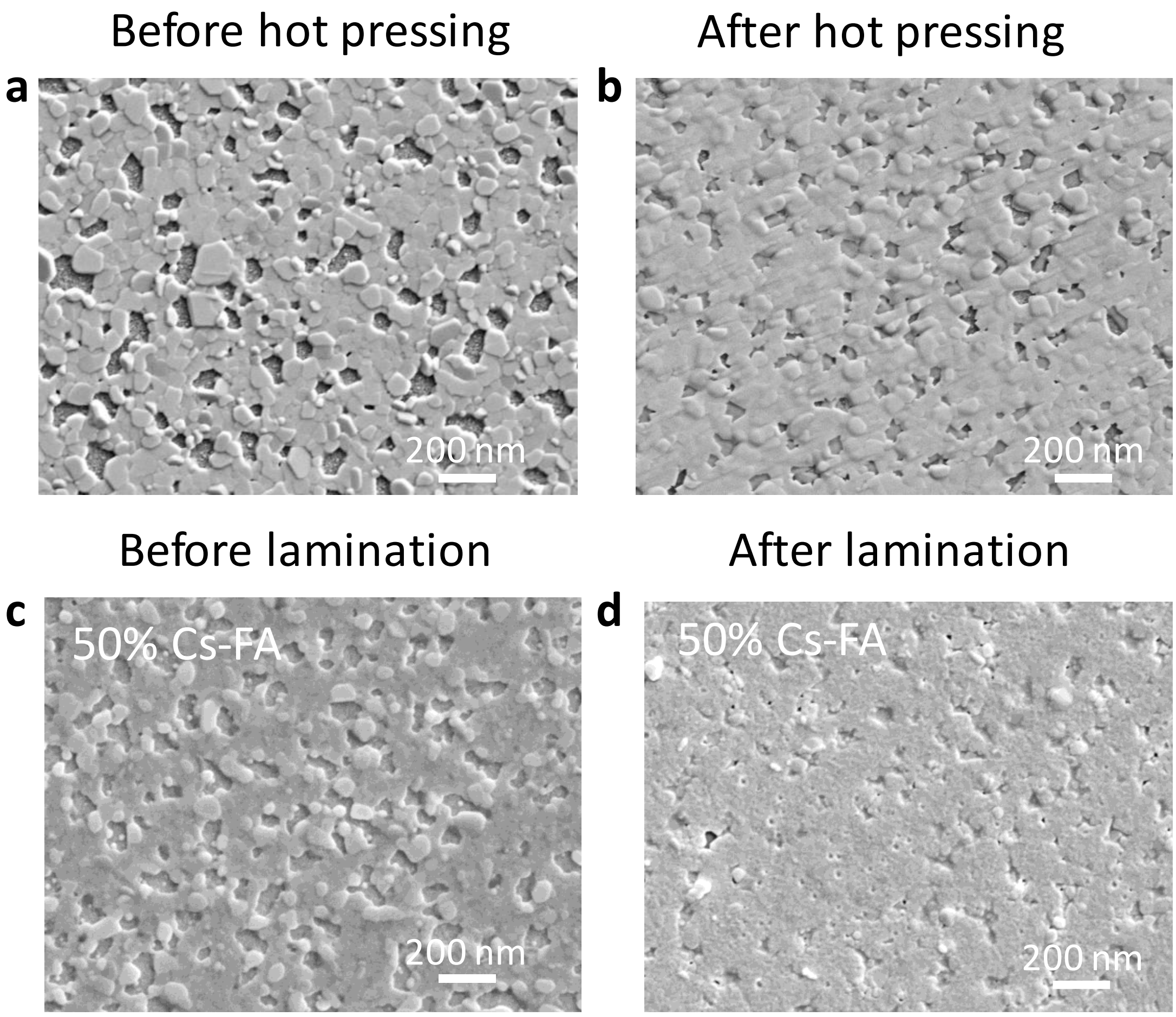

Figure S2. Top-view SEM images of $CsPbBr_3$ films under different processing

conditions. a, b, $CsPbBr_3$ before and after hot pressing process. A clean silicon wafer was used to press on top of $CsPbBr_3$ films under the same pressure and temperature conditions as lamination (i.e., without a second perovskite layer). Hot pressing reduces surface roughness, but the improvement is moderate. c, d, $CsPbBr_3$ film with reduced thickness (50% of the original spin-coating solution concentration) before after lamination with a $FAPbBr_3$ (reduced thickness, 50% of the original spin-coating solution concentration). The morphological change is less pronounced compared to the standard-thickness bilayer, indicating that film thickness plays a critical role in the recrystallization process.

6. In Fig. 3g, the ASE from the bilayer is dominated by a peak near 540 nm, which also appears closer to emission from the FA component. The manuscript does not discuss this sufficiently. This seems difficult to reconcile with the core claim that hot carriers are injected into the CsPbBr3 layer and that the gain enhancement is mainly realized there. The possibility of reabsorption, cavity filtering, or mixed contributions from both layers should be carefully evaluated.

Our response: We thank the reviewer for this insightful comment and have investigated the origin of the ASE signal carefully. Our analysis confirms that the observed gain enhancement is primarily due to hot-carrier transfer into $CsPbBr_3$, not to spectral artefacts or emission from the $FAPbBr_3$ layer.

To further clarify this point, we collected data under high pump fluence conditions (Supplementary Fig. S11a), which reveal two distinct ASE signals – one originating from $CsPbBr_3$ (~540 nm) and another from $FAPbBr_3$ (~556 nm). The $FAPbBr_3$ gain peak at ~556 nm is well separated from the $CsPbBr_3$ gain region, and importantly, the absorption edge of $FAPbBr_3$ is located at ~530 nm. Therefore, the $FAPbBr_3$ layer exhibits only weak absorption at 540 nm, the wavelength where the ASE signal from the laminated heterojunction is observed.

We further compared the fluence-dependent increase in PL intensity at 540 nm for the laminated $CsPbBr_3$/$FAPbBr_3$ heterojunction and for a single-layer $FAPbBr_3$ film.

The results (Supplementary Fig. S11b) show that the single-layer $FAPbBr_3$ film exhibits a well-fitted linear increase in signal intensity up to 100 μJ/cm², with no threshold behaviour. In contrast, even after subtracting the contribution of the $FAPbBr_3$ layer to the 540 nm signal, the heterojunction displays a much lower threshold and a steeper, superlinear rise in emission intensity at 540 nm. This confirms that the ASE signal originates from carriers transferred into $CsPbBr_3$, rather than from direct emission or optical gain from the $FAPbBr_3$ layer.

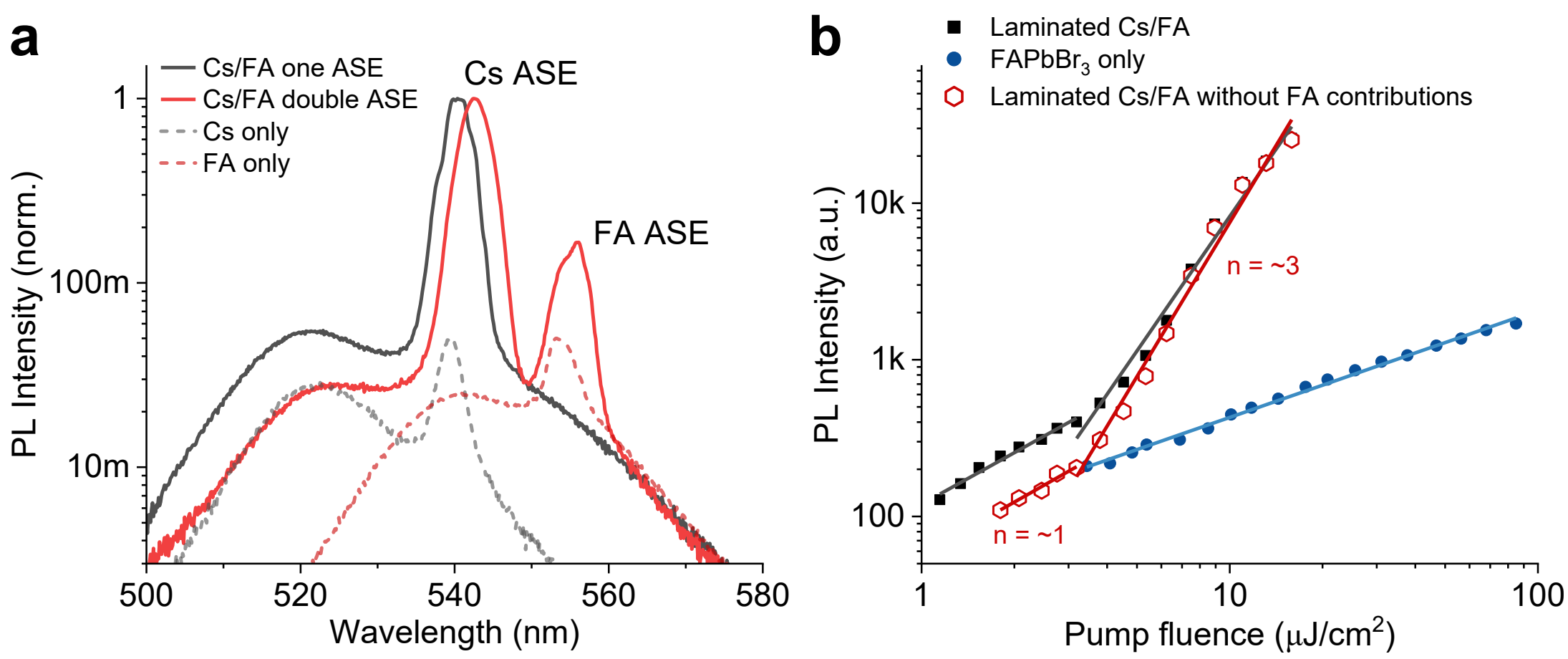

Figure S11. Comparison of ASE signals before and after lamination process. a, PL spectra of the laminated bilayer, pure $CsPbBr_3$, and pure $FAPbBr_3$ films under high-fluence excitation (355 nm, 1 ns pulse width, 1 kHz repetition rate). The laminated heterojunction exhibits two distinct ASE peaks, corresponding to ASE from $CsPbBr_3$ (~540 nm) and $FAPbBr_3$ (~556 nm). b, Fluence-dependent PL intensity at 540 nm for the laminated heterojunction (black squares) and a pure $FAPbBr_3$ film (blue dots). Red hollow dots represent the heterojunction signal after subtracting the contribution of the $FAPbBr_3$ layer at 540 nm. After removing the $FAPbBr_3$ background, the laminated heterojunction also shows a transition from a linear increase (below threshold) to a superlinear increase (above threshold) with a superlinear slope factor of ~3, confirming the onset of ASE originating from carriers transferred into the $CsPbBr_3$ layer. The threshold fluence remains unchanged compared to the original (unsubtracted) data.

We have ***added*** on the Page 14 of revised manuscript, that "We further investigated the origin of the ASE signal to rule out contributions from reabsorption or mixed emission from the $FAPbBr_3$ layer. Under high-fluence excitation (Supplementary Fig. S11a), the laminated heterojunction exhibits two distinct ASE peaks: one at ~540 nm originating from $CsPbBr_3$ and another at ~556 nm from $FAPbBr_3$. The $FAPbBr_3$ gain peak is well separated from the $CsPbBr_3$ gain region, and the absorption edge of $FAPbBr_3$ lies at ~530 nm. Hence, the $FAPbBr_3$ layer absorbs only weakly at 540 nm – the wavelength at which the heterojunction ASE is observed. To quantify the contribution of each layer, we compared the fluence-dependent PL intensity at 540 nm for the laminated heterojunction and a pure $FAPbBr_3$ film (Supplementary Fig. S11b). The single-layer $FAPbBr_3$ film shows a linear increase in signal intensity up to 100 μJ/cm^2, with no threshold behaviour. After subtracting the $FAPbBr_3$ contribution at 540 nm from the heterojunction signal, the remaining intensity exhibits a clear transition from linear to superlinear growth (slope factor ∼3) at a threshold fluence identical to that of the original data. This confirms that the ASE at 540 nm arises from carriers transferred into $CsPbBr_3$, not from direct emission or optical gain from the $FAPbBr_3$ layer."

7. In Fig. 3c, the GSB of the Cs layer in the heterojunction decays to a very low level after 20 ps. However, in Fig. 3e, the GSB of the Cs layer in the two control samples remains highly significant after 20 ps. What accounts for this substantial difference in decay behavior? Are these data from different batches of samples, or are there differences in measurement conditions or probe wavelengths? Similarly, when comparing Fig. 3c and 3f, clear differences in the decay behavior of the FA layer are also observed.

Our Response: We thank the reviewer for pointing out this difference. We clarify that the data shown in Fig. 3c and Fig. 3e, 3f were acquired under different pump fluences.

Fig. 3c (laminated bilayer): Data were taken at a high pump fluence (∼75 μJ/cm^2). Under this condition, a large number of hot carriers are generated in $FAPbBr_3$ and

rapidly transferred to $CsPbBr_3$. The transferred carriers quickly cool and recombine, leading to a fast decay of the $CsPbBr_3$ GSB to a low level within ~20 ps.

Fig. 3e and 3f (PMMA block layer control): These data were acquired at a low pump fluence (~25 μJ/cm^2). Also a PMMA layer was inserted between two perovskite layers during lamination process to prevent any carrier transfer between $FAPbBr_3$ and $CsPbBr_3$. Consequently, each layer behaves independently, and the $CsPbBr_3$ GSB decays only via its intrinsic slower recombination pathways (tens of picoseconds). Because the fluence is lower, the initial GSB amplitude is also smaller, and the decay is less rapid, leaving a significant signal at 20 ps.

Thus, the different decay behaviors are not an inconsistency but a direct consequence of the different excitation fluences and the presence or absence of hot-carrier transfer. At high fluence (Fig. 3c), fast hot-carrier transfer and subsequent recombination dominate, causing rapid decay. At low fluence with the PMMA block (Fig. 3e, 3f), no transfer occurs, and the $CsPbBr_3$ GSB decays more slowly.

We have revised the figure captions to explicitly state the pump fluence used for each measurement (now added to the captions of Fig. 3c, 3e, 3f, see details below). We apologize for the lack of clarity in the original figure labelling and thank the reviewer for the opportunity to correct this.

Figure 3. Hot carrier transfer driven by cooling rate gradients. a, Comparison of TA kinetics between single layers of $CsPbBr_3$ and $FAPbBr_3$ (400 nm pump, ~25 μJ/cm^2, signals were collected at their GSB positions). **b-d,** TA color maps (b), kinetics (c), and spectra (d) of laminated bilayer thin films. The pump laser (400 nm, ~75 μJ/cm^2) was directed at $CsPbBr_3$ side. Global fitting was used to extract hot carrier transfer process in total TA signals (see details in Methods part). The dash line and solid line in (c) represent the fitting of total TA signals and the hot carrier transfer process, respectively. **e, f,** Comparison of TA kinetics between laminated bilayer thin films and films with the same lamination conditions but with a PMMA block layer, which

is designed to act as a blocking layer for the carrier transfer between $CsPbBr_3$ and $FAPbBr_3$. The TA kinetics were obtained from $CsPbBr_3$ (e) and $FAPbBr_3$ (f) side with a pump fluence of ~25 μJ/cm^2, respectively. **g,** Evolution of emission spectra of laminated bilayer thin films with different pump fluences. The pump laser was directed at $CsPbBr_3$ side (400 nm, 270 fs pulse, and 5KHz repetition rate). **h,** PL intensity as a function of the pump fluence. The data were collected from laminated bilayer films with laser impinging on $CsPbBr_3$ sides (Laminated Cs), at $FAPbBr_3$ sides (Laminated FA), pure $CsPbBr_3$, and $FAPbBr_3$ films. **i,** Amplified spontaneous emission (ASE) thresholds as a function of the pump wavelength.
